# Synthesis, Characterization, and Molecular Structure of Some Uranyl Complexes Supported by Hybrid Salicylaldimine/Calix[4]arene Ligands

**DOI:** 10.3390/molecules31132357

**Published:** 2026-07-03

**Authors:** André Busching, Christian Zocher, Martin Börner, Marco Wenzel, Jan J. Weigand, Berthold Kersting

**Affiliations:** 1 Institute of Inorganic Chemistry and Crystallography, Leipzig University, Johannisallee 29, 04103 Leipzig, Germany; andre.busching@uni-leipzig.de (A.B.); christian.zocher@uni-leipzig.de (C.Z.); martin.boerner@uni-leipzig.de (M.B.); 2 Faculty of Chemistry and Food Chemistry, Dresden University of Technology, Mommsenstraße 4, 01062 Dresden, Germanyjan.weigand@tu-dresden.de (J.J.W.); 3 Department of Chemistry and Polymer Science, Stellenbosch University, Stellenbosch 7600, South Africa

**Keywords:** calix[4]arene ligands, uranyl complexes, crystal structure, spectroscopic properties

## Abstract

Three new hybrid bis(salicylaldiminato)/calix[4]arene ligands H_4_L1–H_4_L3 have been synthesized and investigated with regard to their coordination behavior toward the UO_2_^2+^ cation. The ligands H_4_L1 and H_4_L2, derived from bis-1,3-amino-ethoxy-functionalized calix[4]arenes and 3-methoxy-2-hydroxy-salicylaldehydes, react readily with uranyl nitrate in the presence of NEt_3_ to support mononuclear neutral complexes with a 1:1 metal:ligand stoichiometry, namely [UO_2_(H_2_L1)] (**6**) and [UO_2_(H_2_L2)] (**7**). Ligand H_4_L3, with an additional alanyl linker connecting the bis(2-amino-ethoxy)-calix[4]arene backbone and the 3-methoxy-2-hydroxy-salicylaldehyde arms, supports a neutral, mixed-ligand dinuclear uranyl complex [(UO_2_)_2_(MeO)_2_(H_2_L3)] (**8**). The ligands H_4_L1 and H_4_L2 act as pentadentate O_4_N ligands for the UO_2_^2+^ ion to produce a distorted pentagonal bipyramidal coordination environment (O_6_N donor set). The ligand H_4_L3 supports a binuclear [UO_2_(μ-OMe)_2_UO_2_]^2+^ core unit, whose terminal coordination sites are occupied by the donors of the two pendant arms of H_4_L3. The spectroscopic properties (NMR, IR, UV-Vis, ESI-MS) suggest that the complexes **6** and **7** retain their integrity in solution state. The structures are further stabilized by intramolecular hydrogen bonding interactions, as implied by computational analyses (NCI plots). These findings provide valuable insight into the influence of spatial flexibility and donor arrangement on the uranyl coordination chemistry of calix[4]arene-based ligand systems.

## 1. Introduction

**Figure 1:**
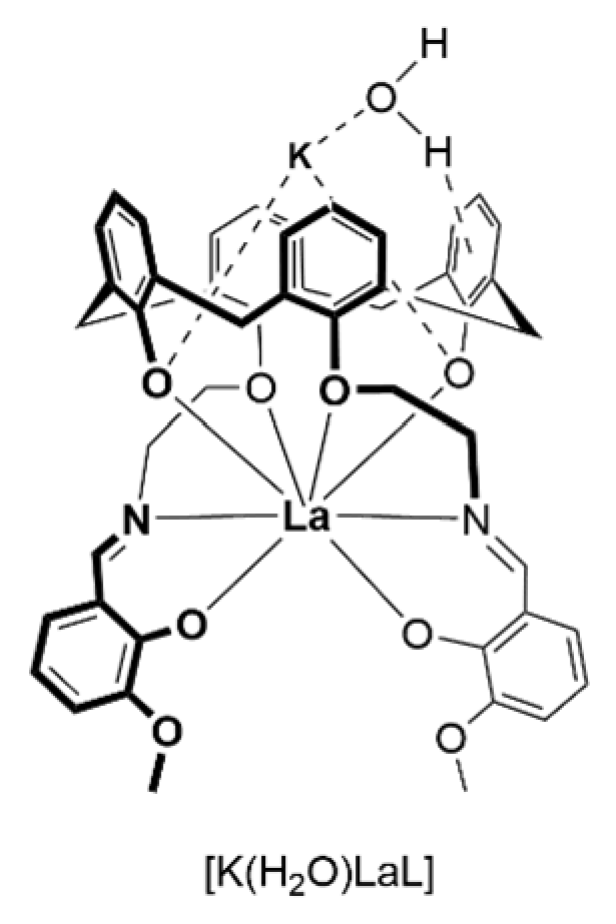
Heterobimetallic potassium/lanthanum complex, supported by the ligand H_4_L.

The coordination chemistry of actinyl ions, particularly the uranyl dication (UO_2_^2+^), is of continued interest due to its importance in nuclear waste remediation [1,2,3,4], environmental monitoring [5], and for the development of selective extraction agents [6,7,8,9,10,11,12,13]. Calix[n]arenes are versatile macrocyclic scaffolds whose preorganized cavities and tunable functionalization make them attractive ligands for uranyl binding [14,15,16,17,18,19,20,21,22,23,24,25,26,27,28,29,30,31,32,33,34]. The linear O=U=O unit, with its rigid geometry and five to six equatorial coordination sites, readily forms stable complexes with calixarenes bearing additional donor groups, such as phosphoryl, amide, or hydroxyquinoline moieties [35,36,37,38]. Some functionalized calix[4]arenes show selectivity for uranyl over lanthanide ions, which is an attractive feature for nuclear waste treatment and rare-earth separations [39]. Calix[4]arenes support diverse uranyl binding modes, ranging from monodentate to multidentate coordination, as well as up to the formation of dimers and capsule like structures. Calix[4]arenes have been functionalized with various ligand functional groups. Of these, Schiff-base ligands have attracted some interest owing to their modular synthesis and adjustable donor environments in classical salen-type and in more elaborate glucosamine derivatives [40]. In addition, Schiff-base ligands have been shown to form complexes with the uranyl ion [41,42,43,44,45,46]. Recently, we reported the synthesis and characterization of lanthanum complexes supported by the pendant calix[4]arene ligand H_4_L. The ligand acts as an octadentate ligand forming a mononuclear complex of the type [KLaL] (Figure 1) [47].

**Scheme 1:**
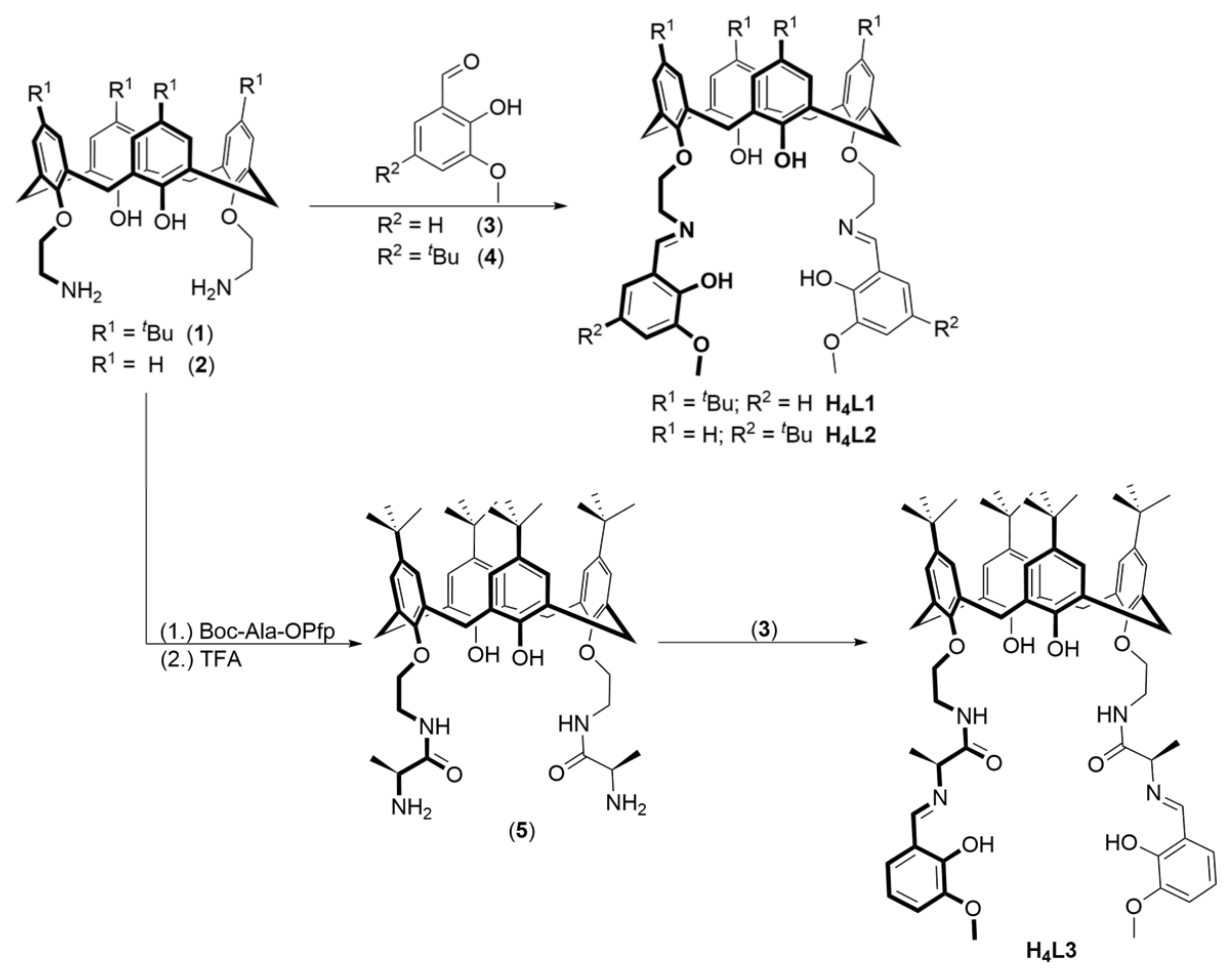
Synthesis of the ligand systems H_4_L1, H_4_L2, and H_4_L3.

In this context, we became interested in the coordination properties of such ligand systems towards the uranyl cation. These ligands were expected to combine the conformationally preorganized calixarene framework with the versatile coordination properties of Schiff-base units, offering the opportunity to study the influence of donor arrangement and spacer design on uranyl complexation. To this end, we have designed and synthesized three such ligands (H_4_L1–H_4_L3, Scheme 1), each incorporating two *ortho*-vanillin-derived salicylaldimine units at the lower rim. As will be shown, subtle variations in ligand architecture determine not only the nuclearity of the resulting complexes but also their spectroscopic and structural features, thus providing new insights into the design principles governing selective uranyl recognition.

## 2. Results and Discussion

### 2.1. Synthesis and Characterization of the Ligands

The ligand H_4_L1 was obtained by an imine condensation reaction between the known calix[4]arene diamine (**1**) and two equiv. of the salicylaldehyde (**3**) in 45% yield (Scheme 1) [48]. The ligand H_4_L2 was obtained in similarly good yields from diamine (**2**) and salicylaldehyde (**4**). Treatment of the calix[4]arene diamine (**1**) with Boc-l-alanine-pentafluorophenyl ester (Boc-D-Ala-OPfp) followed by removal of the carbamate groups furnished with alanine-functionalized bis-amine (**5**), which was subjected to imine condensation with salicylaldehyde (**3**) to give the ligand H_4_L3. In this case, the yield was only 15%.

The new ligands and all intermediate compounds were characterized by elemental analysis; electrospray ionization mass spectrometry (ESI-MS); and IR, UV-vis, ^1^H, and ^13^C NMR spectroscopy. 2D-NMR experiments (COSY, NOESY, HSQC) were used to assign the chemical shifts of hydrogen atoms (see Supplementary Materials for the assignments). Table 1 summarizes the selected analytical data for the ligands.

**Figure 2:**
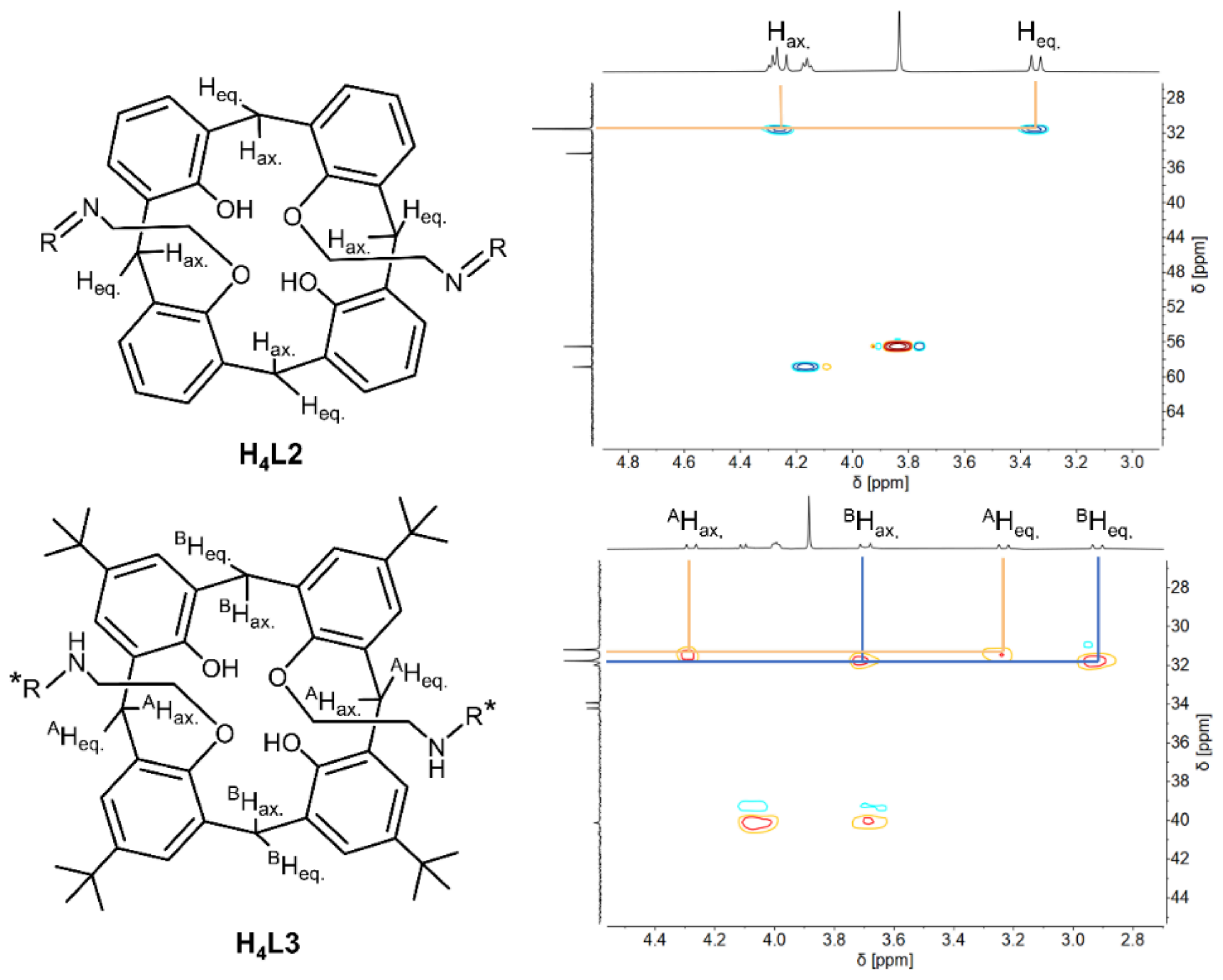
Assignment of the calix[4]arene CH_2_ groups for H_4_L2 (**top**) and H_4_L3 (**bottom**) on the basis of the corresponding ^1^H/^13^C HSQC spectra (Figures S20 and S24). R* represents the introduced chiral substituent in H_4_L3, resulting in 4 AB spin systems. R = salicylaldimine residue.

According to NMR spectroscopic analysis (^1^H, COSY, ^13^C, HSQC; Figures S12–S20, Supplementary Materials) the two ligands H_4_L1and H_4_L2 exist as cone conformers in the liquid state. The ^1^H NMR spectrum for H_4_L1, for example, reveals only two ^1^H NMR signals for the four *tert*-butyl groups (1.25 and 0.98 ppm) and only one signal for the two imine groups (8.65 ppm). Likewise, the methylene protons of the calix[4]arene fragment give rise to two characteristic AB systems centered at 3.40 and 4.40 ppm, the values of which are quite diagnostic for the cone conformation [49]. The ^13^C NMR spectrum for H_4_L1 displays only twelve ^13^C signals for the twenty-four aromatic carbon atoms of the calix[4]arene, also consistent with the cone conformation. The ^1^H NMR spectrum of the ligand H_4_L3 displays four doublets (4 AB spin systems) for the methylene protons of the calix[4]arene unit, while only two such doublets (2 AB systems) are seen for these fragments in the cases of H_4_L1 and H_4_L2 (Figure 2). This spectroscopic difference can be attributed to the presence of a chiral carbon atom located in the alanyl group of the pendant arms, which leads to a lower symmetry of H_4_L3. Thus, the two-fold symmetry in H_4_L1 and H_4_L2 is reduced to *C*_1_ in H_4_L3, thereby producing two distinctly different chemical environments for the Ar-CH_2_-Ar groups. This reduction in symmetry is also reflected in the larger number of observed ^13^C signals in the ^13^C NMR spectrum of H_4_L3 (Figure S22) [50].

Both ligands H_4_L1 and H_4_L2 were also characterized by single-crystal X-ray crystallography (for ORTEP plots, see Supplementary Materials Figures S71 and S72). Suitable crystals for analysis were obtained by recrystallization from ethanol. H_4_L1 crystallizes in the monoclinic space group *C2/c*, whereas H_4_L2 adopts the triclinic space group *P-1*. In both structures, the calix[4]arene backbone adopts a cone conformation, stabilized by intramolecular hydrogen bonds between the unsubstituted and substituted phenolic residues. The salicylaldimine moieties point away from the lower rim of the calix[4]arene, such that the two phenolic oxygen atoms of the calixarene can act as additional coordination sites. This arrangement facilitates the formation of discrete 1:1 metal complexes, in which the calixarene backbone participates in coordination.

### 2.2. Synthesis and Characterization of Uranyl Complexes ***6***–***8***

**Scheme 2:**
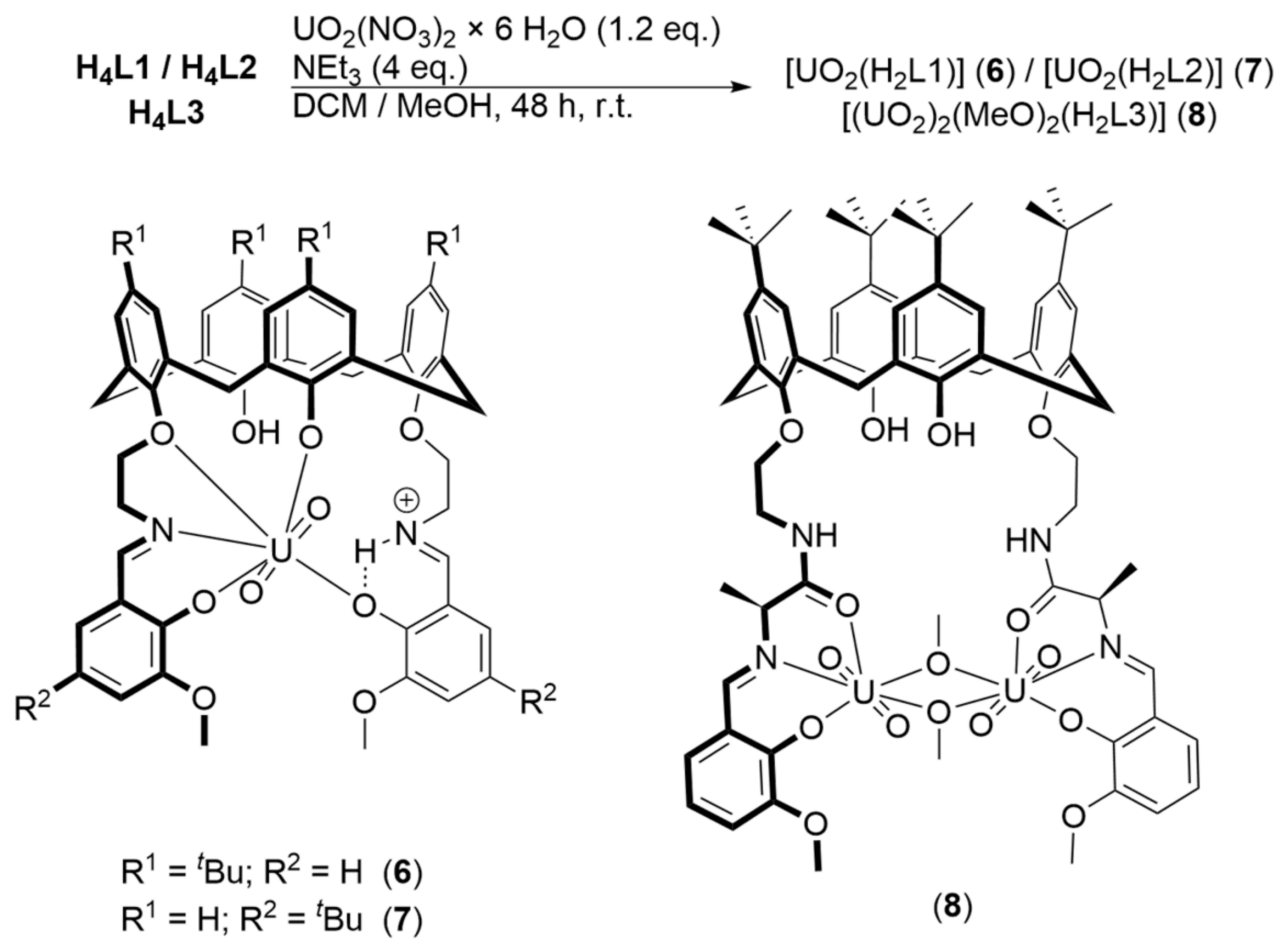
Synthesis of the UO_2_^2+^ complexes **6–8**.

All three ligands were subjected to complexation studies with the UO_2_^2+^ cation in order to investigate their coordination properties. Treatment of a yellow solution of H_4_L1 with UO_2_(NO_3_)_2_^.^6H_2_O and NEt_3_ as a base in a 1:1:4 molar ratio in a mixed CH_2_Cl_2_/MeOH solvent system (Scheme 2) reproducibly resulted in the formation of the orange-to-dark-red-colored, mononuclear, neutral complex [UO_2_(H_2_L1)] (**6**) in 84% yield. The ligand H_4_L2 reacted in the same fashion to produce the orange-to-dark-red-colored neutral complex [UO_2_(H_2_L2)] (**7**) in 89% yield. Under analogous conditions, the reaction between H_4_L3 and uranyl nitrate, followed by slow evaporation of a DCM/MeOH solution, yields a crystalline material comprising [(UO_2_)_2_(MeO)_2_(H_2_L3)] (**8**) in 42% yield. The new uranyl complexes are all air-stable, but become turbid when removed from the mother liquor. The compounds exhibit good-to-moderate solubility in aprotic solvents, such as methylene chloride, chloroform, and THF, but are only sparingly soluble in protic solvents. The identity of all complexes was ascertained by elemental analysis; mass spectrometry; and IR, UV-vis, and NMR spectroscopy, in addition to single-crystal X-ray crystallography.

The presence of a 1:1 metal:ligand stoichiometry in solution is confirmed by ESI(+)-MS spectroscopy for **6** and **7**. Thus, both MS spectra show a molecular ion peak at *m*/*z* = 1271.61 or *m*/*z* = 1159.49 for mononuclear [UO_2_(H_3_L1)]^+^ and [UO_2_(H_3_L2)]^+^ species, respectively, each with the correct isotopic ratio (Figures S59–S61 and S62). The 2:1 metal-to-ligand ratio for dinuclear complex **8** is indicated by a set of molecular ion peaks at *m*/*z* = 841.36, *m*/*z* = 1681.72, and *m*/*z* = 1713.73 for the dinuclear [(UO_2_)_2_(H_2_L3)]^2+^, [(UO_2_)_2_(HL3)]^+^, and [(UO_2_)_2_(H_2_L3)(MeO)]^+^ species, respectively (Table 1, Figures S63–S67). A peak at *m*/*z* = 1413.69 corresponds to a mononuclear species [UO_2_(H_3_L3)]^+^ (Figure S65). However, we have not been able to isolate such a complex.

**Figure 3:**
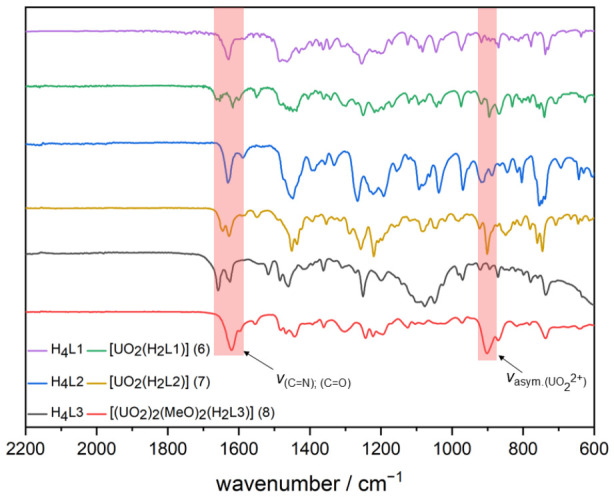
ATR-IR spectra for the reported ligand systems (H_4_L1 (light purple), H_4_L2 (blue), H_4_L3 (black)) and their uranyl complexes **6**–**8**.

Figure 3 displays the obtained ATR-IR spectra for the ligands and complexes. Selected stretching frequencies are listed in Table 1. The most significant feature in the ATR-IR spectrum of the uranyl complex **7** is a band at 901 cm^−1^ for the asymmetric stretching vibration (ν_as_(UO_2_)^2+^) of the uranyl cation. This band is in the usual range for the IR active ν_as_(UO_2_)^2+^ stretching mode [51,52]. For complex **8**, a similar strong band occurs at 901 cm^−1^ also attributed to the ν_as_(UO_2_)^2+^ stretching mode. For **7**, two other prominent bands occur at 1627 cm^−1^ and 1645 cm^−1^. These are tentatively assigned to the ν(C=N) stretches of two different imine groups [53]. All spectral features discussed above are also visible for complex **6**. This situation is in agreement with the solid-state structures of **7** and **6** (vide infra), which reveals two different imine groups, i.e., one imine coordinates to the UO_2_^2+^ group and the other is non-coordinating. The non-coordinating imine is present in a protonated state, resulting in the formation of a H-bonding interaction between the phenolate and the imine appending hydrogen.

The maximum of the band for the ν(CO) stretch in compound **8** peaked at 1620 cm^−1^, while the corresponding band maxima in the ligand is observed at 1658 cm^−1^. Thus, upon complexation of the ligand by UO_2_^2+^, the ν(CO) is red-shifted by ~38 cm^−1^, consistent with coordination of the amide group. Raman spectra recorded for **7** display a strong band at 790 cm^−1^ (Figure S48), which can be correlated to the symmetric UO_2_^2+^ vibration. The shift relative to UO_2_(NO_3_)_2_^.^6H_2_O (ν_symm._ = 869 cm^−1^) is related to the change in the coordination sphere [54].

### 2.3. Electronic Absorption Spectroscopy

**Figure 4:**
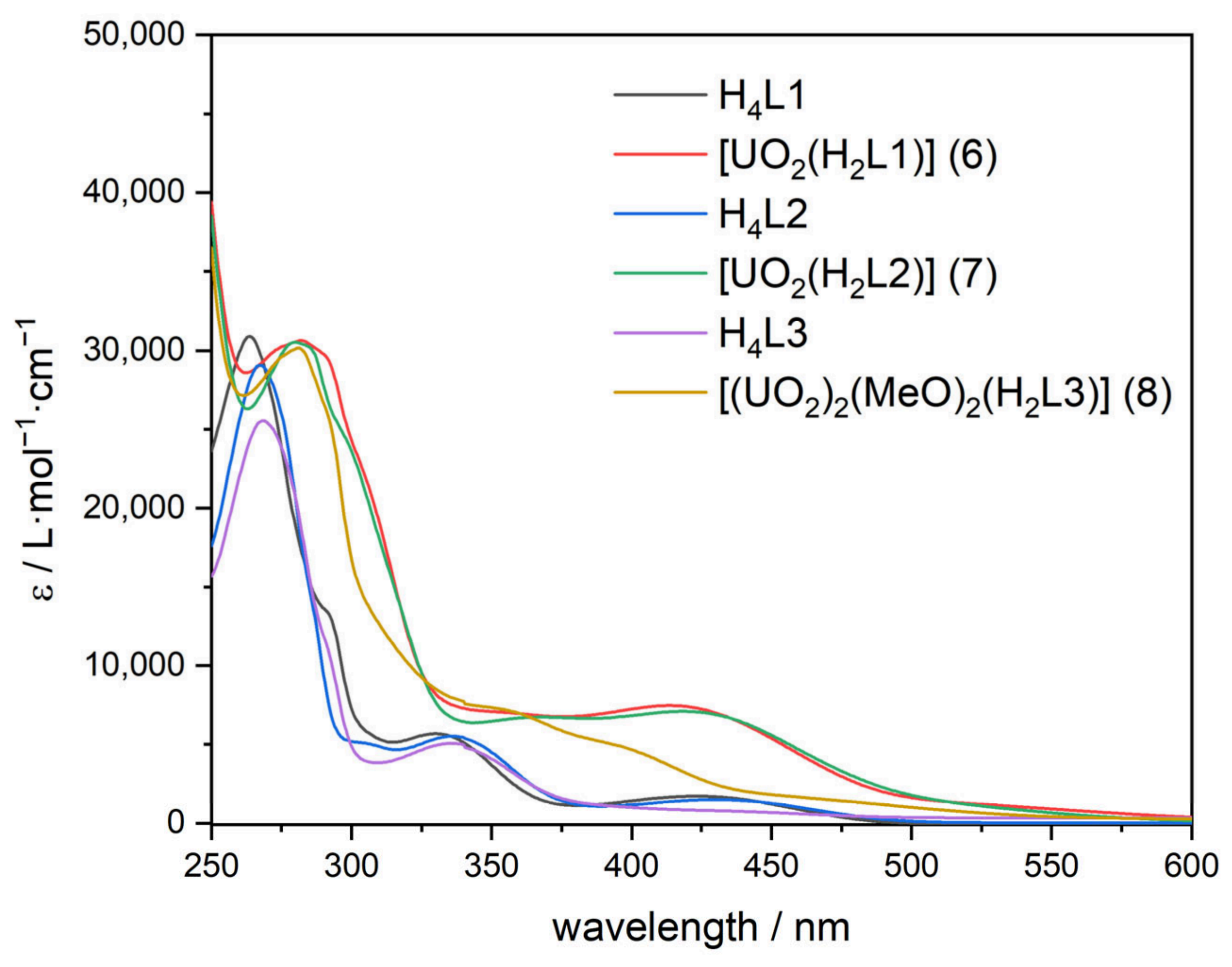
Electronic absorption spectra for the ligands H_4_L1–H_4_L3 and their uranyl complexes **6**–**8** in DCM/MeOH (1:1, *v*:*v*) solution (concentration: 5.0 × 10^–5^ M, 295 K).

Electronic absorption spectra of the ligands and complexes were recorded in a mixed DCM/MeOH solution, in which the complexes had the best solubility. Figure 4 displays the resulting spectra. Solutions of the free ligands exhibit a yellow color. In their UV-vis spectra, there are two intense absorption bands at ~325 nm and 425 nm attributable to transitions into ^1^(π-π*) states of the (iminomethyl)phenol chromophores [55]. The other intense band at higher energies of 275 nm can be attributed to a transition into the ^1^(π-π*) states of the aromatic rings of the calix[4]arene moiety [56]. The complexation of the ligands with UO_2_^2+^ ions is accompanied by a color change from yellow to red–orange (Figure S70). As expected, UV-vis spectra of the UO_2_^2+^ complexes **6**–**8** differ from those of the free ligands. The spectrum of complex **7** is representative. It exhibits two well-resolved absorption bands at ~280 nm and 425 nm, both with higher intensity than the absorptions of H_4_L2. Two shoulders are observed at ~305 nm and ~350 nm. The two high-energy features in the UV region are most likely associated with ^1^(π-π*) states within the aromatic rings of the UO_2_^2+^-bound calix[4]arene units. The broad band at 425 nm, on the other hand, is assigned to an intraligand transition to a ^1^(π-π*) state involving the (iminomethyl)phenolato chromophore. Relative to the corresponding absorption of the free ligand, the intensity is about 5 times higher. The electronic absorption spectral properties of the other complexes are very similar to those of the UO_2_^2+^ complex **7**. Thus, the complexation of the free ligands by the UO_2_^2+^ cations leads to a bathochromic shift in the intraligand π-π* absorptions and is accompanied by a strong hyperchromic effect. The color changes produced upon complexation can be attributed to this hyperchromic effect. Thus, these ligands may be interesting to investigate as chemosensors for the naked eye detection of the UO_2_^2+^ cation in liquid state. Similar intense color changes and shifts as well as the appearance of new bands at ~410 nm are well-reported characteristics for uranyl complexes supported by Schiff-base ligands.

### 2.4. NMR Spectroscopy for ***6***–***8***

**Figure 5:**
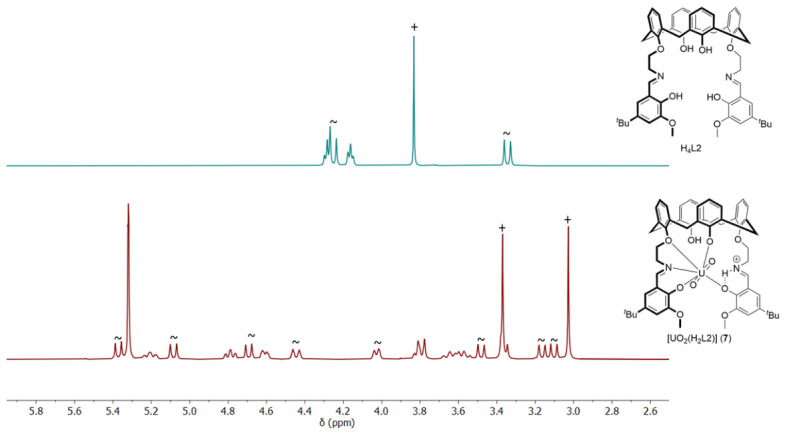
Comparison of the ^1^H-NMR spectra for the ligand H_4_L2 (blue) and its uranyl complex [UO_2_(H_2_L2)] **7** (red) in the 6–2 ppm region. The peaks labeled with + and ~ correspond to OMe- and methylene groups, respectively.

Complexes **6** and **7** were further characterized by ^1^H NMR spectroscopy (Figures S25–S32). The NMR spectrum for **7** in CD_2_Cl_2_ solution is representative (Figure 5) and will be discussed. Only one set of signals is observed. The most diagnostic signals are the two intense singlets at 1.32 ppm and 1.39 ppm, attributed to the *t*-butyl groups of two chemically different *ortho*-vanillin moieties. The imine groups also become inequivalent upon complex formation (9.31 ppm (s), 8.26 ppm (^3^*J*_H,H_ = 14.1 Hz, HC = N^+^H)). The presence of two chemically different methoxy groups (3.03 ppm, 3.37 ppm) should also be mentioned. The splitting of all these signals implies that the two ligand arms in complex **7** are chemically different. This suggests that the solid-state structure of complex **7** (see below) is retained in solution. The same conclusion can be drawn from analysis of the ^1^H NMR spectra for complex **6**. Unfortunately, the ^1^H NMR spectrum for complex **8** (Figure S33) showed only broad signals and could not be analyzed further.

### 2.5. Crystallographic Characterization

#### 2.5.1. Description of the Crystal Structure of [UO_2_(H_2_L1)]·2.75CH_2_Cl_2_·MeOH (6·2.75CH_2_Cl_2_·MeOH)

**Figure 6:**
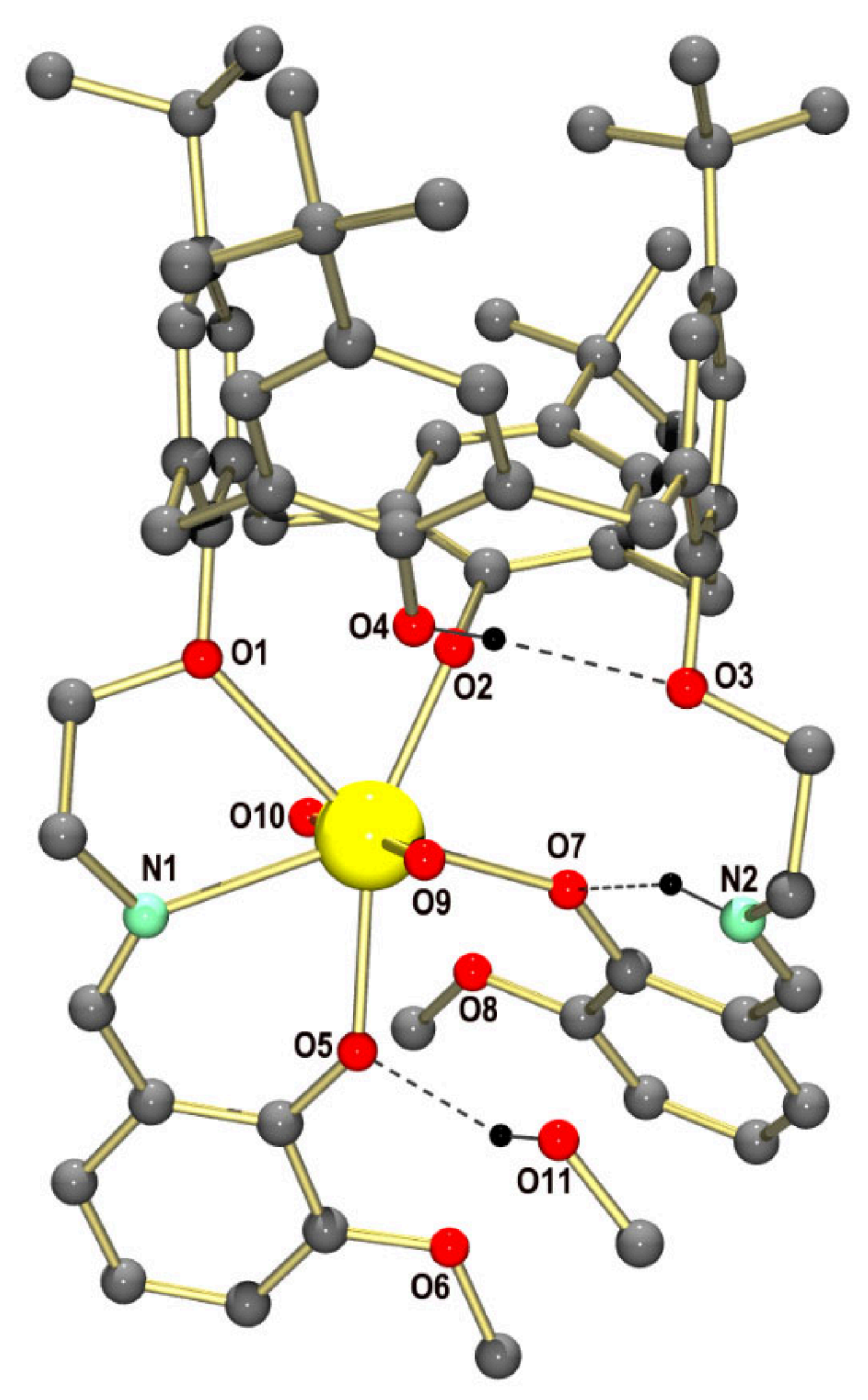
Ball-and-stick representation of the molecular structure of the [UO_2_(H_2_L1)] complex in crystals of [UO_2_(H_2_L1)]^.^2.75CH_2_Cl_2_·MeOH (**6**·2.75CH_2_Cl_2_·MeOH), as determined by single-crystal X-ray diffraction. Solvate molecules and H atoms, except those involved in hydrogen bonding interactions, have been omitted for clarity. Selected bond lengths [Å] and angles [°]: U-O1 2.732(3), U-O2 2.211(3), U-O5 2.324(3), U-O7 2.347(3), U-O9 1.779(4), U-O10 1.777(4), U-N1 2.579(4), and O9-U-O10 176.19(14).

Single crystals of [UO_2_(H_2_L1)]·2.75CH_2_Cl_2_·MeOH suitable for X-ray crystallographic analyses were obtained by slow evaporation from a mixed CH_2_Cl_2_/MeOH solution. The crystal structure revealed the presence of well-separated [UO_2_(H_2_L1)] complexes and co-crystallized H_2_O and CH_2_Cl_2_ solvate molecules. Figure 6 displays the structure of the mononuclear uranyl complex. The uranyl atom is seven-coordinated, where it is coordinated by the two oxo groups, one phenolate O, and one phenolether O atom from the calix[4]arene unit, as well as two phenolate O atoms and one imine N atom from the pendant imine arms. The coordination geometry is best described as distorted pentagonal bipyramidal, which is a typical geometry for UO_2_^2+^ complexes. The O=U=O angle at 176.2° and the U=O bond lengths of 1.779 and 1.777 Å show no unusual features and are normal for UO_2_^2+^ ions (Table 2). The U-O^phenolato^ bonds range from 2.211 Å to 2.347 Å. The U-O phenolato bonds involving salicylaldimine are significantly longer than those involving the phenolato group from the calix[4]arene, which may be indicative of different bonding abilities. The distortion from the ideal pentagonal bipyramidal geometry is manifested by the rather long U-O^phenolether^ (2.732 Å) and U-N^imine^ (2.579 Å) bonds. Charge considerations suggest that the non-coordinated imine function is in a protonated state (assuming a doubly charged UO_2_^2+^ ion and a singly charged CH=NHR^+^ iminium ion, which compensate for the three negatively charged phenolato (ArO^−^) residues). The structure is further stabilized by intramolecular (N2^…^O7 2.649 Å, O3^…^O4 3.009 Å) and intermolecular H bonding interactions (O5^…^O11 2.815 Å Figure 6). The calix[4]arene ligand remains in the cone conformation, but the two-fold symmetry is lost upon coordination of the UO_2_^2+^ ion.

#### 2.5.2. Description of the Crystal Structure of [UO_2_(H_2_L2)]·CH_2_Cl_2_ (7·CH_2_Cl_2_)

**Figure 7:**
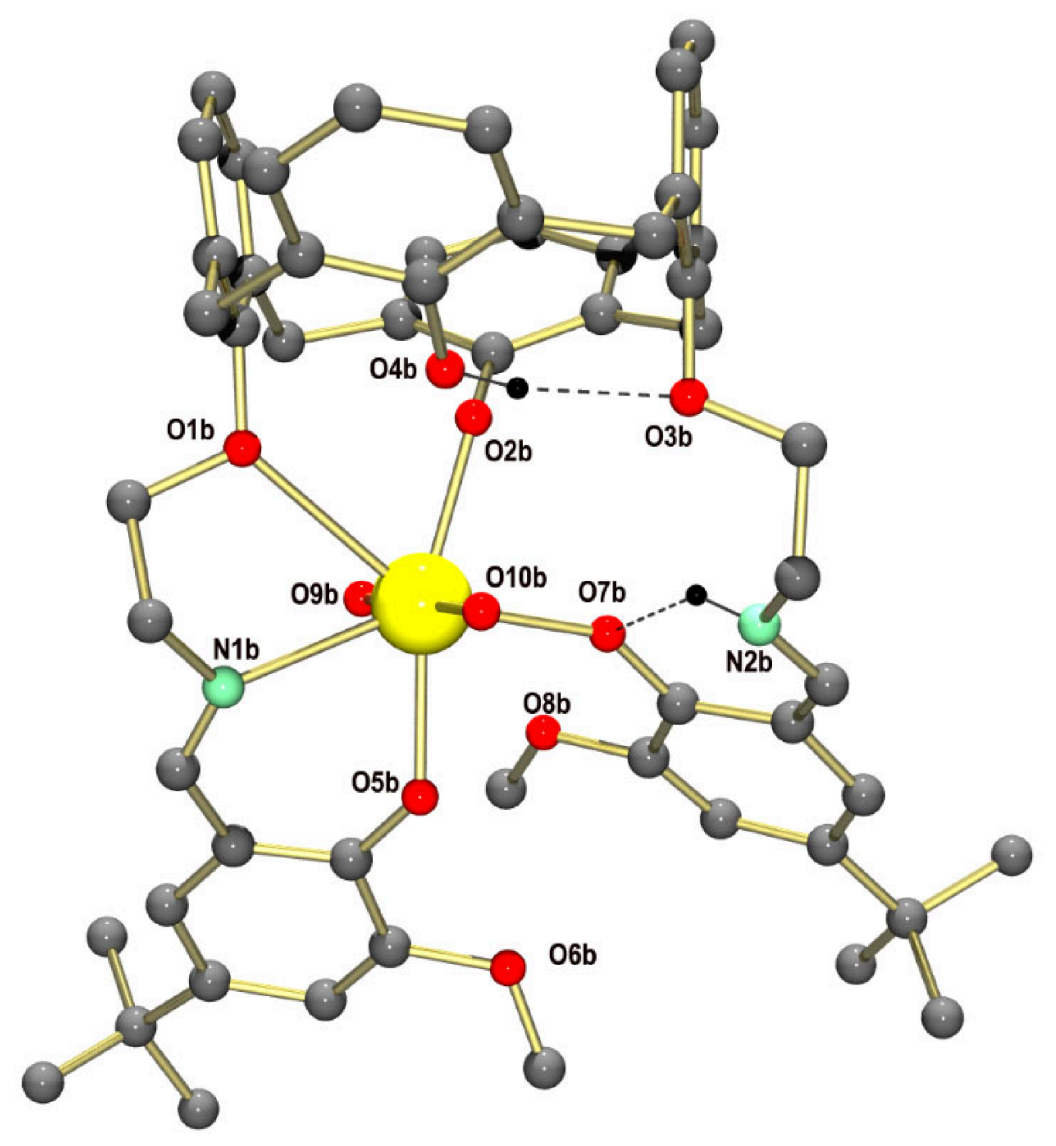
Ball-and-stick representation of the molecular structure of the [UO_2_(H_2_L2)] complex (molecule B) in crystals of [UO_2_(H_2_L2)]^.^CH_2_Cl_2_ (**7**·CH_2_Cl_2_), as determined by single-crystal X-ray diffraction. H atoms, except those involved in hydrogen bonding interactions and CH_2_Cl_2_ solvate molecules, have been omitted for clarity. Selected bond lengths [Å] and angles [°] (values in square bracket correspond to molecule A): U1-O1 2.833(15) [2.871(14)]; U1-O2 2.240(1) [2.253(12)]; U1-O5 2.249(13) [2.237(13)]; U1-O7 2.344(13) [2.348(14)]; U1-O9 1.751(18) [1.746(17)]; U1-O10 2.045(18) [2.021(17)]; U1-N1 2.523(18) [2.593(18)]; and O9-U1-O10 176.2(6) [177.6(6)].

Crystals of [UO_2_(H_2_L2)]·CH_2_Cl_2_ (**7**·CH_2_Cl_2_) obtained from a mixed CH_2_Cl_2_/MeOH solution are of the monoclinic space group *C*2*/c*. The quality of this data set is not as good as that of the previous compound and can only prove the atom connectivity. The asymmetric unit contains two neutral [UO_2_(H_2_L2)] complexes (hereafter denoted as molecules A and B) and several dichloromethane solvate molecules. Most of the CH_2_Cl_2_ molecules were heavily disordered, and so the corresponding electron density was removed with the SQUEEZE routine implemented in Platon. Complex **7** is essentially isostructural with complex **6**, indicating that the *t*-Bu groups exert little structural influence on either the calix[4]arene or the salicylaldimine. Figure 7 shows the structure of molecule B along with selected bond lengths and angles. The atom labeling utilized for **6** was adapted for **7** to aid structural comparisons. The H_2_L2 ligand wraps around the UO_2_^2+^ ion in the same fashion as in **6**. Likewise, the free imine group is in a protonated state and forms an intramolecular H bond with the phenolate atoms of the supporting ligand. Again, four sets of U-L bond distances can be distinguished and ranked as follows: U=O < U-O^phenolate^ < U-N^imine^ < U-O^phenolether^. The corresponding metal–ligand bond lengths in **6** and **7** differ by no more than 0.13 Å; however, given the poor quality of the dataset, this finding should be taken as indicative rather than definitive. The packing of the [UO_2_(H_2_L2)] complexes in **6** and **7** is very different as one might expect in view of the different placement of the *t*-Bu substituents on the ligand.

**Figure 8:**
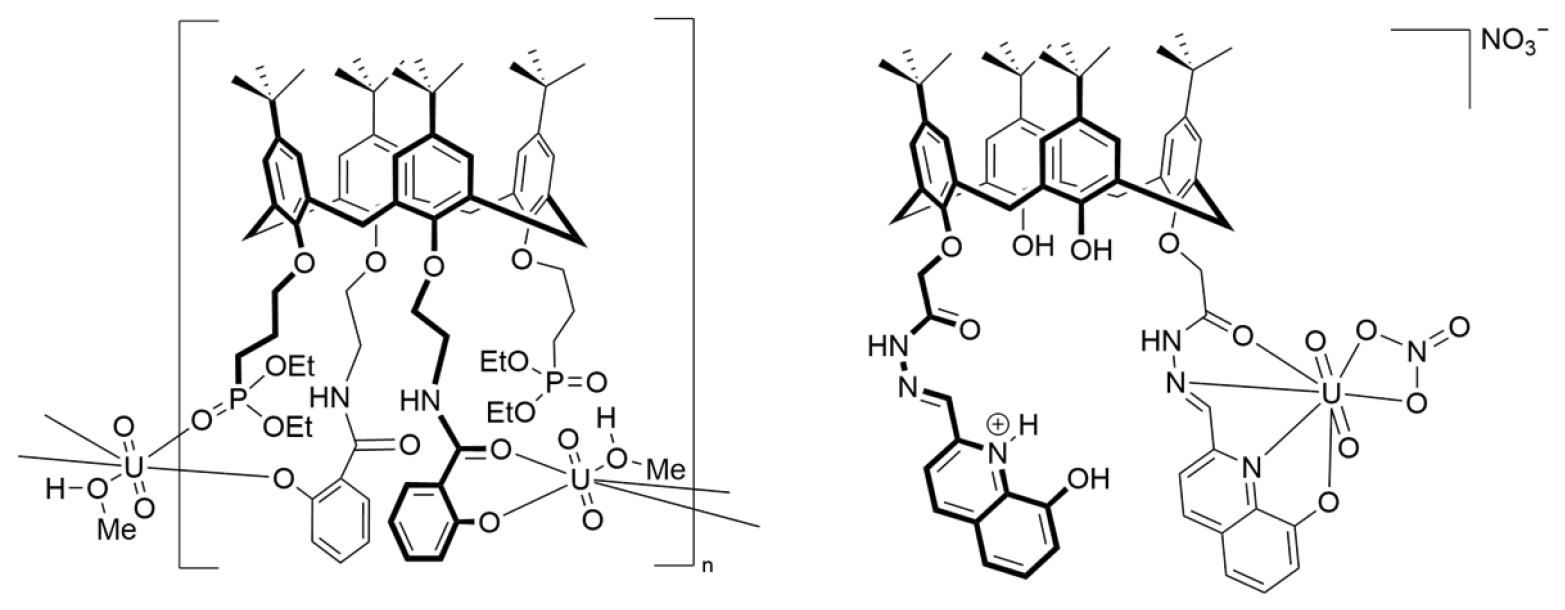
Comparison of coordination modes of pendant calix[4]arenes with the uranyl cation (authenticated by X-ray crystallography) [36,38].

A comparison of the complexation behavior of the ligands H_4_L1 and H_4_L2 is appropriate at this stage. The binding mode of these two ligands, as seen in the uranyl complexes **6** and **7**, differs significantly from those seen in other uranyl complexes supported by pendant calix[4]arene ligands (Figure 8). The synthesis and structure of a relevant UO_2_^2+^ complex, supported by a calix[4]arene ligand containing lower-rim phosphoryl and salicylamide functional groups, were reported [36]. The complexation of the uranyl cation required less donor atoms than those supplied by H_2_L^Phos^. Thus, some potential binding sites of the ligand do not participate in the complexation, and there is no formation of an isolated mononuclear complex, as observed in the case of Ln^3+^ ions. Instead, this ligand was found to form polymeric complexes of the type [UO_2_(MeOH)(L^Phos^)]*_n_*^.^5*n*MeOH. Another relevant example is [UO_2_(H_6_L^Qui^)(NO_3_)](NO_3_) supported by a hybrid calix[4]arene ligand with two appended hydroxyquinoline units [38]. Again, the uranyl ion is coordinated by only one of the two 8-hydroxyquinoline–hydrazone–carbonyl substituent from H_6_L^Qui^. One ligand arm remains non-coordinating. The present structures clearly demonstrate the potential of the reported ligands H_4_L1 and H_4_L2 to form discrete mononuclear species.

#### 2.5.3. Description of the Crystal Structure of [(UO_2_)_2_(MeO)_2_(H_2_L3)]·14(MeOH)

**Figure 9:**
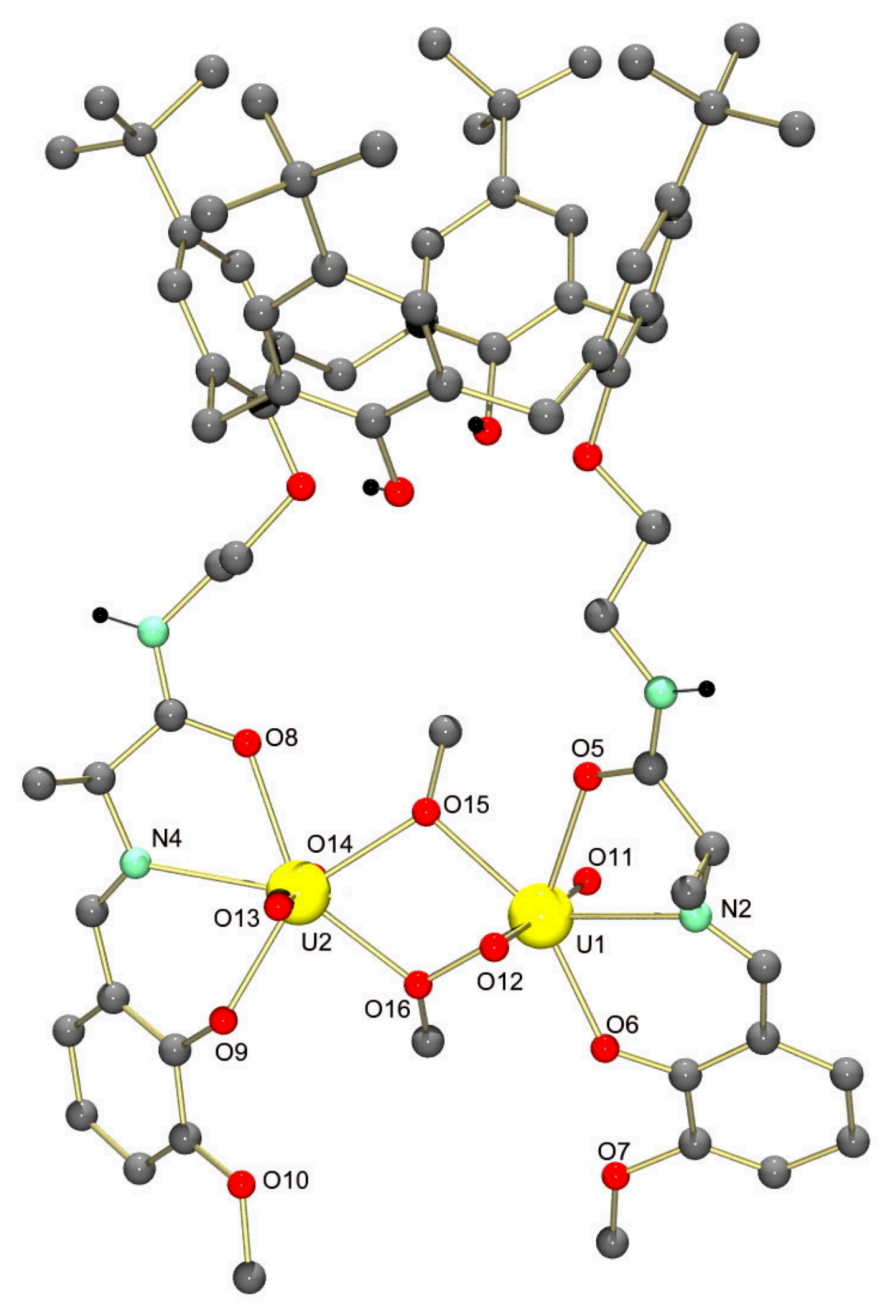
Single-crystal X-ray diffraction structure of the [(UO_2_)_2_(OMe)_2_(H_2_L3)] complex (molecule A) in crystals of [(UO_2_)_2_(OMe)_2_(H_2_L)]·14MeOH (**8**·14MeOH). Ball-and-stick representation. Solvate molecules and H atoms omitted for clarity.

Orange-colored crystals of the uranyl complex (**8**·14MeOH) suitable for X-ray crystallographic analysis were obtained by slow evaporation from a CH_2_Cl_2_/MeOH solution. Crystals of the title compound are of the triclinic space group *P-1*. The asymmetric unit comprises three molecules of a neutral, dinuclear [(UO_2_)_2_(MeO)_2_(H_2_L3)] complex **8** and several (~14) heavily disordered solvate molecules. The latter could not be refined and so were removed from the structure using the SQUEEZE routine implemented in Platon [57]. The molecular structure of one of the three independent molecules (molecule A) is shown in Figure 9. The uranyl complex has idealized *C*_s_ symmetry comprising a central UO_2_(μ-OMe)_2_UO_2_ unit, which is coordinated by the carbonyl O, the imine N, and the phenolato groups of the two pendant Schiff-base arms. Charge considerations imply the presence of a doubly deprotonated form (H_4_L3-2H)^2−^ of the supporting ligand (deprotonated o-hydroxy-aryl groups) and two methanolato ligands. The uranium atoms are thus seven-fold coordinated in a distorted pentagonal bipyramidal coordination geometry. The UO_2_^2+^ ions are almost linear with O-U-O angles of 179.0° and 179.5°. The average U=O bond length at 1.777 Å is normal. The other U-O bond lengths vary significantly from 2.225 to 2.438 Å over all three independent molecules. The U-O(phenolato) bonds are significantly shorter than the U-O bonds involving the bridging methanolato ligands. The amide carbonyl O atoms form the longest U-O bonds (U1-O5 2.429(5), U1-O8 2.415(6) Å).

### 2.6. Non-Covalent Interactions in Complexes ***6*** and ***8***

To gain a deeper understanding of intramolecular interactions NCI plot analysis was performed, based on the calculated molecular wavefunction of the optimized structure for **6** and a calculated single-point energy of the crystal structure for **8** (for more details, see Supplementary Materials Tables S2 and S3). By analyzing the electron density of the molecule and its gradients, interactions like hydrogen bonding, van der Waals forces, and steric repulsions are visualized in real space using color-coded isosurfaces according to their corresponding fingerprint plot. The resulting plots allow spatially resolved qualitative analysis of intramolecular contacts, distinguishing attractive (blue), van der Waals (green), and repulsive interactions (red) [58]. The presence of O-H^…^O hydrogen bonds stabilizes the cone-conformation. QTAIM analysis was performed for compound **6**. This did not reveal a bond critical point for a hydrogen bonding interaction between the UO_2_^2+^ ion and the iminium H. This is in agreement with the poor hydrogen bond acceptor ability of the uranyl oxygens [59,60,61].

## 3. Experimental Section

### 3.1. Materials and Methods

All reagents were purchased from commercial sources and used as received. The calix[4]arene-based diamines **1** and **2** were prepared as described in the literature [62]. The aldehyde **4** as well as Boc-Ala-OPfp-ester were prepared following the literature procedure [63,64]. Automated column chromatography was performed using a BIOTAGE Select ENKEL system (Biotage, Uppsala, Sweden). NMR spectra were recorded on a Bruker FT 300 spectrometer or AVANCE DRX 400 spectrometer at 298 K (Bruker, Billerica, MA, USA). Chemical shifts refer to solvent signals. The spectra are supplied in the Supplementary Materials, along with the atomic numbering scheme utilized for assignment of the signals. Mass spectra were obtained using the positive ion or negative electrospray ionization modus (ESI) on a Bruker Daltronics ESQUIRE 3000 Plus ITMS or Impact II UHR Qq-TOF instrument. ATR-infrared spectra were recorded using a Thermoscientific Nicolet iS5 (Thermo Fischer Scientific, Waltham, MA, USA) or a Bruker Vertex 80v in the area between 4000 and 400 cm^−1^. Raman spectra were recorded at ambient temperature using a Bruker Vertex 70 instrument equipped with a RAM II module (Nd-YAG laser, 1064 nm). Raman intensities are reported in percentages relative to the most intense peak and are given in parenthesis. All UV-vis spectra were recorded using a JASCO V770 (JASCO International Co., Ltd., Tokyo, Japan) with quartz cuvettes of 1 cm path length. Elemental analysis was carried out using the elementar UNICUBE analyzer (Elementar Analysensysteme GmbH, Langenselbold, Germany) and reported herein, nevertheless as mentioned by other authors, the elemental analyses of calixarenes are very often incorrect because of inclusion of solvent molecules and cannot be considered an appropriate criterion of purity [65,66].

### 3.2. X-Ray Crystallography

Suitable specimens for single-crystal X-ray diffraction were selected and mounted on a cryoloop using perfluoropolyether oil. The data sets were collected at 180(2) K or 130(2) K on a STOE STADIVARI X-ray diffractometer equipped with a GeniX 3D Cu-HF (Xenocx) microfocus X-ray source, with a graded multilayer mirror (Cu-Kα, λ = 1.54186 Å) and a hybrid pixel detector Pilatus3 300K (Dectris). Data processing was carried out with the STOE X-Area 2.7 software, including a spherical absorption correction and scaling routine. Structures were solved with SHELXT 2018/2 [67] using dual methods and refined full-matrix least-squares techniques on the basis of all data against F2, using version 2018/3 SHELXL [68] and Olex2 [69]. The coordinates of all non-hydrogen and non-disordered atoms were refined with anisotropic thermal parameters. Hydrogen atoms were included in idealized positions. In cases of highly disorder solvent molecules, the SQUEEZE routine, as implemented in Platon, was applied to remove diffuse electron density.

### 3.3. Theoretical Calculations

The electron density of the uranyl complexes was calculated in ORCA 6.0.0 [70,71] by the one-parameter version of the Perdew–Burke–Ernzerhof hybrid DFT (PBE0) [72] method with Grimmes D4 dispersion correction [73,74,75], using a def2-TZVP basis set [76] for all atoms except uranium and a scalar relativistic SARC-ZORA-TZVPP basis set [77] for the uranium.

NCI analysis was performed using the nciplot [78] and muliwfn [79] tools utilizing the calculated electron densities. The full workflow, including all scripts, plots, inputs, and outputs generated in this project, are available on Zenodo [80].

### 3.4. Synthesis of Compounds

Boc-Di-Ala-Calix[4]arene. A solution of 5,11,17,23-tetra-*p*-*tert*-butyl-25,27-bis(aminoethoxy)-26,28-dihydroxycalix[4]arene (**3**) (331 mg, 450 µmol, 1 eq.) and Boc-Ala-OPfp-ester (330 mg, 900 µmol, 2 eq.) in chloroform (25 mL) was stirred at room temperature for 24 h. The reaction mixture was then washed with 5% sodium carbonate solution (2 × 20 mL) followed by 1 M HCl (2 × 20 mL). The organic phase was dried with Na_2_SO_4_, filtered, and evaporated to dryness. The residue was purified by flash column chromatography using ethyl acetate in *n*-hexane (12–100%). Yield: 189 mg (176 µmol, 39%). ^1^H-NMR (400 MHz, CDCl_3_) δ [ppm] = 8.64 (s, 2H, C[4]a-OH), 8.50 (br, 2H, -C_2_H_4_-NH), 7.02 (m, 8H, *meta*-H-C[4]a-OH + *meta*-H-C[4]a-O_sub._, C^4/6/10/12/16/18/22/24^), 5.32 (d, 2H, *J* = 8.2 Hz, Ala-NH,), 4.30 (br, 8H, Ala-CH + 2x Ar-CH_ax._H-Ar + O-CHHCH_2_-NH, C^2/8/14/20/29a/32/34a/37^), 3.99 (br, 4H, OCHHCH_2_NH + OCH_2_CHHNH, C^29b/34b/30a/35a^), 3.74 (br, 2H, OCH_2_CHHNH, C^30b/35b^), 3.44 (d, 2H, *J* = 13.5 Hz, Ar-CH_eq._H-Ar, C^2/14^), 3.35 (d, 2H, *J* = 12.8 Hz, Ar-CH_eq._H-Ar, C^8/20^), 1.42 (s, 18H, O_sub_.-Ar-C(CH_3_)_3_, C^11/23^), 1.34 (m, 6H, Ala-CH_3_, C^33/38^), 1.24 (s, 18H, HO-Ar-C(CH_3_)_3_, C^5/17^), 1.11 (s, 18H, Boc-C(CH_3_)_3_). ^13^C-NMR (100 MHz, CDCl_3_) δ [ppm] = 173.18, 155.28, 149.32, 148.87, 148.29, 143.10, 133.27, 127.95, 125.99, 125.82, 75.79, 65.93, 49.85, 39.48, 34.35, 34.04, 32.14, 31.74, 31.70, 31.26, 29.21, 28.54, 27.89, 27.07, 24.53, 22.96, 22.80, 19.79, 18.91. ESI-MS (+) *m/z* 1077.68 [M+H^+^] (calc. 1077.68). IR (ATR-IR) ṽ [cm^−1^] = 3325 (br), 2955 (s), 2905 (m), 2867 (m), 1710 (s), 1672 (s), 1667 (s), 1513 (sh), 1484 (s), 1460 (s), 1390 (m), 1362 (s), 1299 (m), 1241 (m), 1194 (s), 1164 (s), 1123 (m), 1095 (w), 1047 (s), 1025 (sh), 981 (w), 945 (w), 920 (w), 870 (w), 821 (w), 799 (w), 780 (w), 756 (w), 708 (w), 634 (w), 588 (w), 552 (w), 494 (w), 482 (w), 470 (w), 461 (w), 431 (w). Elemental analysis calcd. (%) for C_64_H_92_N_4_O_10_: C 71.34 H 8.61 N 5.20; found: C 71.36 H 8.63 N 4.88.

Di-Ala-Calix[4]arene (**5**). Boc-di-Ala-calix[4]arene (200 mg, 185 µmol, 1 eq.) was dissolved in DCM (4 mL) and TFA (4 mL) was added as a deprotection agent. The resulting solution was stirred for 1 h at room temperature, before removing excess reagent and solvent under reduced pressure. The resulting off-white oil was neutralized using a saturated sodium bicarbonate solution and extracted with DCM (3 × 50 mL). The solution was dried using anhydrous MgSO_4_ and the solvent removed under reduced pressure. Yield: 157 mg (179 µmol, 96%). ^1^H-NMR (400 MHz, CDCl_3_) δ [ppm] = 8.35 (t, 2H, *J* = 5.6 Hz, -C_2_H_4_-NH), 7.65 (s, 2H, C[4]a-OH), 7.05 (s, 4H, *meta*-H-C[4]a-O_sub._, C^10/12/22/24^), 6.84 (s, 4H, *meta*-H-C[4]a-OH, C^4/6/16/18^), 4.20 (m, 4H, Ar-CH_ax._H-Ar, C^2/8/14/20^), 4.08 (m, 4H, O-CH_2_-CH_2_-N, C^29/34^), 3.86 (m, 4H, O-CH_2_-CH_2_-N, C^30/35^), 3.63 (m, 2H, Ala-H, C^32/37^), 3.34 (d, 4H, *J* = 13.1 Hz, Ar-CH_eq._H-Ar, C^2/8/14/20^), 1.37 (d, 6H, *J* = 6.9 Hz, Ala-CH_3_, C^33/38^), 1.27 (s, 18H, O_sub_.-Ar-C(CH_3_)_3_, C^11/23^), 0.98 (s, 18H, HO-Ar-C(CH_3_)_3_, C^5/17^). ^13^C-NMR (100 MHz, CDCl_3_) δ [ppm] = 176.50, 150.00, 148.98, 147.74, 142.48, 132.69, 127.89, 127.81, 125.91, 125.52, 51.30, 39.49, 34.17, 34.02, 31.78, 31.14, 24.53, 21.61. ESI-MS (+) 878.60 *m/z* [M+H^+^] (calc. 878.59). IR (ATR-IR) ṽ [cm^−1^] = 3341 (br), 2953 (s), 2904 (m), 2866 (m), 1663 (s), 1523 (m), 1483 (s), 1461 (s), 1361 (s), 1299 (m), 1240 (m), 1195 (s), 1123 (s), 1096 (m), 1046 (s), 978 (w), 946 (w), 918 (m), 870 (s), 839 (w), 820 (m), 798 (m), 780 (m), 756 (m), 734 (w), 718 (m), 710 (m), 676 (w), 664 (sh), 643 (m), 590 (m), 552 (m), 526 (w), 502 (w), 484 (m), 471 (w), 464 (sh), 454 (w), 433 (w), 423 (sh), 407 (w). Elemental analysis calcd. (%) for C_54_H_76_N_4_O_6_: C 73.94 H 8.73 N 6.39; found: C 72.01 H 8.60 N 5.62.

H_4_L1. A solution of 5,11,17,23-tetra-*tert*-butyl- 25,27-bis(2′-aminoethyl)-26,28-dihydroxycalix[4]arene (750 mg, 1 mmol, 1 eq.) and 2-hydroxy-3-methoxybenzaldehyde (34 mg, 2.2 mmol, 2.2 eq.) in ethanol (75 mL) was refluxed for 16 h. The solvent was partially removed under reduced pressure and the solution stored at 5 °C for 48 h. The resulting yellow precipitate was filtered, washed with cold ethanol (35 mL), and dried at 60 °C. Yield: 0.45 g (0.449 mmol, 45%). ^1^H-NMR (400 MHz, CDCl_3_) δ [ppm] = 13.57 (s, 2H, -ArOH), 8.57 (s, 2H, N=CH, C^31/41^), 7.00 (s, 4H, *meta*-H-C[4]a-O_sub_, C^10/12/22/24^), 6.95 (dd, 4H, *J* = 7.8 Hz, 1.5 Hz, *meta*-H-Ar-OMe, C^35/45^), 6.87 (dd, 4H, *J* = 8.0 Hz, 1.5 Hz, *meta*-H-Ar-C=N, C^33/43^), 6.78 (m, 4H, *para*-H-Ar + C[4]a-OH, C^34/44^), 6.72 (s, 4H, *meta*-H-C[4]a-OH, C^4/6/16/18^), 4.27 (t, 4H, *J* = 5.9 Hz, O-CH_2_-CH_2_-N, C^29/39^), 4.21 (d, 4 H, *J* = 13.1 Hz, Ar-CH_ax._H-Ar, C^2/8/14/20^), 4.12 (t, 4H, *J* = 5.8 Hz, O-CH_2_-CH_2_-N, C^30/40^), 3.83 (s, 6H, Ar-OCH_3_, C^38/48^), 3.28 (d, 4H, *J* = 13.1 Hz, Ar-CH_eq._H-Ar, C^2/8/14/20^), 1.28 (s, 18H, O_sub_.-Ar-C(CH_3_)_3_, C^11/23),^ 0.90 (s, 18H, HO-Ar-C(CH_3_)_3_, C^5/17^). ^13^C-NMR (100 MHz, CDCl_3_) δ [ppm] = 167.90, 151.80, 150.58,149.79, 148.47, 147.14, 141.52, 132.39, 127.85, 125.70, 125.15, 123.57, 118.91, 118.08, 114.47, 75.08, 58.65, 56.28, 34.01, 33.94, 31.84, 31.76, 31.09. ESI-MS (+) 1003.58 *m/z* [M+H^+^] (calc. 1003.578). IR (ATR-IR) ṽ [cm^−1^] = 3397 (br), 3052 (m), 3003 (s), 2961 (s), 2919 (s), 2904 (s), 2868 (s), 2835 (m), 2715 (m), 2668 (m), 1628 (s, C=N), 1484 (s), 1464 (s), 1430 (m), 1418 (m), 1393 (w), 1373 (w), 1362 (m), 1344 (m), 1311 (w), 1254 (s), 1200 (m), 1169 (w), 1124 (m), 1092 (m), 1082 (s), 1045 (s), 972 (s), 869 (m), 778 (m), 737 (s), 729 (m), 710 (w), 636 (m). UV-Vis (DCM/MeOH 1:1 *v*/*v*) λ_max_ [nm] = 229, 263, 292 (sh), 330, 424. Elemental analysis calcd. (%) for C_64_H_78_N_2_O_8_: C 76.61 H 7.84 N 2.79. Found C, 76.21 H 7.86 N 2.50. This compound was additionally characterized by X-ray crystallography.

H_4_L2. A solution of 25,27-bis(2′-aminoethyl)-26,28-dihydroxycalix[4]arene (450 mg, 0.88 mmol, 1 eq.) and 5-*tert*-Butyl-2-hydroxy-3-methoxybenzaldehyde (381 mg, 1.83 mmol, 2.1 eq.) in ethanol (75 mL) was refluxed for 24 h. The solution was concentrated under reduced pressure and stored at 5 °C for 48 h to give a yellow precipitate, which was filtered, washed with cold ethanol (35 mL), and dried at 60 °C. Yield: 345 mg (0.387 mmol, 44%). ^1^H-NMR (400 MHz, CDCl_3_) δ [ppm] = 13.26 (s, 2H, Ar-OH), 8.65 (s, 2H, N=CH, C^31/41^), 7.52 (s, 2H, C[4]a-OH), 6.99 (d, 4H, *J* = 7.5 Hz, *meta*-H-C[4]a-O_sub._, C^10/12/22/24^), 6.93 (d, 2H, *J* = 2.2 Hz, tBu-ArH-OMe, C^35/45^), 6.88 (d, 2H, *J* = 2.2 Hz, tBu-ArH-sub., C^33/43^), 6.84 (d, 4H, *J* = 7.5 Hz, *meta*-H-C[4]a-OH, C^4/6/16/18^), 6.70 (t, 2H, *J* = 7.5 Hz, *para*-H-C[4]a-O_sub._, C^11/23^), 6.60 (t, 2H, *J* = 7.5 Hz, *para*-H-C[4]a-OH, C^5/17^), 4.28 (m, 8H, Ar-CH_ax._H-Ar+O-CH_2_-CH_2_-N, C^2/8/14/20/29/39^), 4.16 (t, 4H, *J* = 5.6 Hz, O-CH_2_-CH_2_-N, C^30/40^), 3.83 (s, 6H, Ar-OCH_3_, C^38/48^), 3.34 (d, 4H, *J* = 13.1 Hz, Ar-CH_eq._H-Ar, C^2/8/14/20^), 1.27 (s, 18H, Ar-C(CH_3_)_3_, C^34/44^). ^13^C-NMR (100 MHz, CDCl_3_) δ [ppm] = 168.49, 153.21, 151.78, 149.45, 147.92, 141.21, 133.13, 129.19, 128.60, 127.97, 125.64, 119.74, 119.09, 118.08, 113.17, 75.61, 58.85, 56.51, 34.34, 31.56, 31.51. ESI-MS (+) 891.46 *m/z* [M+H^+^] (calc. 891.46). IR (ATR-IR) ṽ [cm^−1^] = 3373 (br), 2947 (m), 2923 (m), 2898 (sh), 2870 (m), 2836 (w), 1630 (s), 1588 (w), 1447 (s), 1393 (m), 1387 (m), 1357 (w), 1332 (w), 1265 (s), 1230 (sh), 1221 (s), 1192 (s), 1155 (m), 1122 (w), 1092 (s), 1081 (s), 1063 (m), 1037 (s), 969 (s), 916 (m), 911 (sh), 888 (m), 865 (w), 844 (m), 816 (m), 803 (m), 754 (s), 746 (s), 738 (s), 693 (w), 643 (m), 629 (m), 604 (m), 593 (sh), 575 (w), 549 (w), 526 (w), 510, 485, 480 (m), 467 (w), 452 (w). UV-Vis (DCM/MeOH 1:1 *v*/*v*) λ_max_ [nm] = 229, 267, 305, 337, 433. Elemental analysis calcd. (%) for C_56_H_62_N_2_O_8_: C 75.48 H 7.01 N 3.14 O 14.36, found: C 74.81 H 6.83 N 3.06 O 14.12. This compound was additionally characterized by X-ray crystallography.

H_4_L3. To a solution of di-ala-calix[4]arene (157 mg, 179 µmol, 1 eq.) in DCM (5 mL), 2-hydroxy-3-methoxybenzaldehyde 57 mg (376 µmol, 2 eq.) was added, followed by ethanol (50 mL). The resulting reaction mixture was stirred under reflux for 18 h, and then cooled to room temperature before partially removing the DCM/ethanol mixture under reduced pressure. The concentrated solution was stored at 5 °C for at least 24 h to give a precipitate, which was filtered, washed with cold ethanol, and dried at 60 °C. Yield: 30 mg (26 µmol, 15%). ^1^H-NMR (400 MHz, CDCl_3_) δ [ppm] = 13.20 (s, 2H, Ar-OH), 8.19 (s, 2H, N=CH, C^34/47^), 8.08 (s, 2H, C[4]a-OH), 7.96 (t, 2H, *J* = 5.6 Hz, -C_2_H_4_-NH), 6.96 (d, 2H, *J* = 2.4 Hz, *meta*-H-C[4]a-O_sub._, C^10/22^), 6.91 (dd, 2H, *J* = 8.1, 1.4 Hz, *meta*-H-Ar-C=N, C^36/49^), 6.87 (d, 2H, *J* = 2.4 Hz, *meta*-H-C[4]a-O_sub._, C^12/24^), 6.79 (t, 4H, *J* = 2.2 Hz, *meta*-H-C[4]a-OH, C^4/6/16/18^), 6.69 (t, 2H, *J* = 7.9 Hz, *para*-H-Ar, C^37/50^), 6.37 (dd, 2H, *J* = 7.8, 1.4 Hz, *meta*-H-Ar-OMe, C^38/51^), 4.28 (d, 2H, *J* = 12.7 Hz, Ar-CH_ax._H-Ar, C^8/20^) 4.05 (m, 8H, O-CH-H-CH_2_-N+O-CH_2_-CH_2_-N+Ala-H, C^29a/30/32/42a/43/45^) 3.88 (s, 6H, Ar-OCH_3_, C^41/54^), 3.70 (m, 4H, Ar-CH_ax._H-Ar+O-CH-H-CH_2_-N, C^2/14/29b/42b^), 3.23 (d, 2H, *J* = 12.8 Hz, Ar-CH_eq._H-Ar, C^8/20^), 2.92 (d, 2H, *J* = 13.1 Hz, Ar-CH_eq._H-Ar, C^2/14^), 1.55 (d, 6H, *J* = 6.9 Hz Ala-CH_3_, C^33/46^), 1.23 (s, 18H, O_sub_.-Ar-C(CH_3_)_3_, C^11/23^), 1.03 (s, 18H, OH-Ar-C(CH_3_)_3_, C^5/17^). ^13^C-NMR (100 MHz, CDCl_3_) δ [ppm] = 172.25, 166.88, 151.16, 149.54, 148.85, 148.28, 147.74, 142.27, 133.43, 133.21, 127.83, 127.76, 126.08, 125.78, 125.71, 125.39, 123.91, 118.90, 118.42, 114.80, 75.89, 68.94, 56.35, 40.14, 34.22, 33.94, 31.99, 31.76, 31.21, 20.65. ESI-MS (+) 1145.66 *m/z* [M+H^+^] (calc. 1145.657). IR (ATR-IR) ṽ [cm^−1^] = 3334 (br), 2958 (m), 2933 (sh), 2864 (w), 1658 (s), 1624 (s), 1517 (m), 1484 (s), 1460 (s), 1418 (w), 1361 (w), 1308 (w), 1271 (m), 1250 (s), 1199 (s), 1156 (sh), 1141 (sh), 1114 (sh), 1100 (s), 1077 (s), 1049 (s), 1025 (sh), 982 (m), 970 (m), 920 (w), 894 (w), 870 (m), 798 (w), 779 (m), 736 (s), 591 (br), 425 (w), 413 (w)**.** UV-Vis (DCM/MeOH 1:1 *v*/*v*) λ_max_ [nm] = 229, 268, 292 (sh), 337, 434. Elemental analysis calcd. (%) for C_70_H_88_N_4_O_10_ C 73.40 H 7.74 N 4.89, found: C 72.70 H 7.77 N 4.72.

[(UO_2_)(H_2_L1)] (**6**). To the calix[4]arene ligand H_4_L1 (51 mg, 51 µmol, 1 eq.) in a mixed DCM/MeOH solution (1:1 *v*/*v*, 20 mL), neat NEt_3_ (28.2 µL, 203 µmol, 4 eq.) was added, followed by solid UO_2_(NO_3_)_2_^.^6H_2_O (31 mg, 61 µmol, 1.2 eq.). After the resulting mixture was stirred for 24 h at r.t., the DCM was removed under reduced pressure to give a red–brown solid, which was then filtered, washed with cold MeOH, and dried at 60 °C. Isolation of the pure product proved to be difficult due to similarities in solubility between the ligand H_4_L1 and the resulting metal complex, as indicated by NMR spectroscopy. Therefore, the reported peaks for the ^13^C-NMR include signals corresponding to the free ligand. Yield: 54 mg (70% with regard to H_4_L1). ^1^H-NMR (400 MHz, CD_2_Cl_2_) δ [ppm] = 14.23 (s, 1H, HC=N^+^H), 9.28 (s, 1H, HC=N, C^31^), 8.26 (d, 1H, *J* = 14.1 Hz, HC=N^+^H, C^41^), 7.30 (d, 1H, *J* = 2.6 Hz, *meta*-HAr, C^12^), 7.23 (d, 1H, *J* = 2.4 Hz, *meta*-HAr, C^24^), 7.14 (dd, 1H, *J* = 7.9, 1.6 Hz, *meta*-HAr, C^33^), 7.10 (d, 1H, *J* = 2.6 Hz, *meta*-HAr, C^10^), 7.07 (dd, 1H, *J* = 8.2, 1.6 Hz, C^34^), 7.02 (m, 2H, 2 x *meta*-HAr, C^22/43^), 6.96 (dd, 1H, *J* = 7.8, 1.6 Hz, *meta*-HAr, C^35^), 6.92 (m, 1H, *meta*-HAr, C^16^), 6.77 (*pseudo*-t, 1H, *J* = 7.9 Hz, *para*-HAr, C^45^), 6.71 (s, 2H, 2 x *meta*-HAr, C^4/6^), 6.67 (d, 1H, *J* = 2.5 Hz, *meta*-HAr, C^18^), 6.56 (*pseudo*-t, 1H, *J* = 7.7 Hz, *para*-HAr, C^44^), 6.22 (s, 1H, C[4]a-OH), 5.37 (d, 1H, *J* = 12.3 Hz, Ar-CH_ax._H-Ar, C^2^), 5.22 (m, 1H, O-CH_2_-CH-H-N, C^40a^), 5.11 (d, 1H, *J* = 12.8 Hz, Ar-CH_ax._H-Ar, C^8^), 4.79 (ddd, 1H, *J* = 11.8, 9.8, 2.3 Hz, O-CH_2_-CH-H-N, C^40b^), 4.69 (d, 1H, *J* = 12.5 Hz, Ar-CH_ax._H-Ar, C^20^), 4.56 (ddd, 1H, *J* = 9.9, 4.0, 2.0 Hz, O-CH-H-CH_2_-N, C^39a^), 4.41 (d, 1H, *J* = 13.6 Hz, O-CH-H-CH_2_-N, C^39b^), 4.03 (dt, 1H, *J* = 9.5, 2.5 Hz, O-CH-H-CH_2_-N, C^29a^), 3.81 (m, 1H, O-CH-H-CH_2_-N, C^29b^), 3.75 (d, 1H, *J* = 13.6 Hz, Ar-CH_ax._H-Ar, C^14^), 3.57 (m, 2H, O-CH_2_-CH_2_-N, C^30^), 3.40 (s+d, 4H, Ar-OCH_3_ + Ar-CH_eq._H-Ar, C^20/38^), 3.31 (d, 1H, *J* = 13.7 Hz, Ar-CH_eq._H-Ar, C^14^), 3.18 (d, 1H, J = 12.4 Hz, Ar-CH_eq._H-Ar, C^2^), 3.13 (s, 3H, Ar-OCH_3_, C^48^), 3.05 (d, 1H, *J* = 12.9 Hz, Ar-CH_eq._H-Ar, C^8^), 1.35 (s, 9H, C(CH_3_)_3_, C^23^), 1.33 (s, 9H, C(CH_3_)_3_, C^11^), 0.95 (s, 9H, C(CH_3_)_3_, C^17^), 0.90 (s, 9H, C(CH_3_)_3_, C^5^). ^13^C-NMR (100 MHz, CD_2_Cl_2_) δ [ppm] = 171.25, 169.37, 166.76, 165.33, 161.84, 155.67, 155.29, 151.61, 151.15, 151.05, 149.49, 148.04, 147.60, 146.71, 141.42, 139.29, 134.72, 133.70, 133.14, 133.10, 130.87, 129.90, 128.39, 127.38, 126.82, 126.74, 126.49, 126.42, 126.19, 125.89, 125.80, 125.32, 125.04, 124.82, 124.57, 124.27, 123.73, 123.31, 119.33, 118.52, 118.40, 116.44, 116.03, 115.41, 114.88, 114.68, 79.88, 75.04, 64.50, 56.45, 55.76, 52.07, 34.47, 34.29, 34.17, 34.06, 33.68, 32.44, 32.38, 32.20, 31.96, 31.33, 31.28, 29.64. ESI-MS (+) 1271.610 *m/z* [M+H^+^] (calc. 1271.608). IR (ATR-IR) ṽ [cm^−1^] = 3503 (br), 2954 (m), 2902 (m), 2866 (m), 1663 (m), 1653 (m), 1648 (m), 1616 (s), 1600 (m), 1547 (m), 1481 (s), 1472 (s), 1464 (s), 1456 (s), 1448 (s), 1436 (s), 1433 (s), 1404 (m), 1380 (w), 1360 (m), 1340 (m), 1296 (m), 1272 (m), 1250 (s), 1219 (s), 1205 (s), 1194 (s), 1169 (m), 1119 (m), 1111 (w), 1092 (m), 1072 (w), 1043 (m), 1030 (m), 973 (m), 914 (m), 893 (s), 866 (s), 829 (m), 801 (w), 781 (w), 762 (m), 752 (m), 740 (s), 705 (m), 646 (w), 624 (m), 597 (w), 570 (w), 537 (w), 523 (m), 513 (w), 488 (w), 451 (w), 416 (w). UV-Vis (DCM/MeOH 1:1 *v*/*v*) λ_max_ [nm] = 282, 305, 417 (7449). Elemental analysis calcd. (%) for C_64_H_76_N_2_O_10_U ∙ 2CH_2_Cl_2_: C 55.00 H 5.60 N 1.94, found: C 54.17, H 5.63, N 2.01.

[(UO_2_)(H_2_L2)] (**7**). To the calix[4]arene ligand H_4_L2 (44 mg, 49 µmol, 1 eq.) in a mixed DCM/MeOH solution (1:1 *v*/*v*, 20 mL), neat NEt_3_ (27.4.2 µL, 197 µmol, 4 eq.) was added, followed by solid UO_2_(NO_3_)_2_^.^6H_2_O (29.8 mg, 59 µmol, 1.2 eq.). After the resulting mixture was stirred for 24 h at r.t., the DCM was removed under reduced pressure, to give an orange–red solid, which was then filtered, washed with cold MeOH, and dried at 60 °C. Yield: 51 mg (89% with regard to H_4_L2). ^1^H-NMR (400 MHz, CD_2_Cl_2_) δ [ppm] = 14.15 (s, 1H, HC=N^+^H), 9.31 (s, 1H, HC=N, C^31^), 8.26 (d, 1H, *J* = 14.1 Hz, HC=N^+^H, C^41^), 7.22 (m, 2H, *meta*-HAr, *meta*-HAr, C^4/12^), 7.18 (d, 1H, *J* = 2.4 Hz, tBu-ArH-OMe, C^35^), 7.11 (d, 1H, *J* = 2.4 Hz, tBu-ArH-OMe, C^45^), 7.04 (m+d, 3H, ArH, *meta*-HAr, *meta*-HAr, C^10/6/43^), 7.00 (d, 1H, *J* = 2.4 Hz, ArH, C^33^), 6.75 (m, 3H, *para*-HAr, *meta*-HAr, C^11/22/24^), 6.64 (m, 4H, *para*-HAr, *para*-HAr, *meta*-HAr, C^17/16/18/23^), 6.35 (t, 1H, *J* = 7.3 Hz, *para*-H-C[4]a-OH, C^5^), 6.14 (s, 1H, C[4]a-OH), 5.37 (d, 1H, *J* = 12.5 Hz, Ar-CH_ax._H-Ar, C^2^), 5.20 (m, 1H, O-CH-H-CH_2_N, C^29a^), 5.08 (d, 1H, *J* = 13 Hz, Ar-CH_ax._H-Ar, C^8^), 4.79 (m, 1H, O-CH_2_-CH-H-N, C^30a^), 4.69 (d, 1H, *J* = 12.8 Hz, Ar-CH_ax._H-Ar, C^20^), 4.61 (m, 1H, O-CH_2_-CH-H-N, C^30b^), 4.45 (d, 1H, *J* = 13.5 Hz, O-CH-H-CH_2_N, C^29b^), 4.03 (d, 1H, *J* = 9.3 Hz, O-CH-H-CH_2_N, C^39a^), 3.80 (d+t, 2H, Ar-CH_ax._H-Ar+O-CH-H-CH_2_N non coord., C^14^/^39b^), 3.59 (m, 2H, O-CH_2_-CH_2_-N, C^40^), 3.48 (d, 1H, *J* = 12.8 Hz, Ar-CH_eq._H-Ar, C^20^), 3.37 (s+d, 4H, Ar-OCH_3_+Ar-CH_eq._H-Ar, C^14/38^), 3.16 (d, 1H, *J* = 12.6 Hz, Ar-CH_eq._H-Ar, C^2^), 3.10 (d, 1H, *J* = 13.1 Hz, Ar-CH_eq._H-Ar, C^8^), 3.03 (s, 3H, Ar-OCH_3_, C^48^), 1.39 (s, 9H, C(CH_3_)_3_-Ar, *t*Bu-C^34^), 1.32 (s, 9H, C(CH_3_)_3_-Ar, *t*Bu-C^44^). ^13^C-NMR (100 MHz, CD_2_Cl_2_) δ [ppm] = 169.91, 169.76, 167.61, 166.83, 159.74, 157.73, 154.99, 153.88, 152.26, 150.78, 139.18, 138.67, 135.97, 134.98, 133.54, 133.52, 131.80, 130.61, 129.60, 129.35, 128.85, 128.59, 128.27, 127.95, 127.57, 127.49, 125.10, 124.52, 122.18, 120.84, 120.78, 120.60, 118.75, 116.24, 115.53, 114.02, 79.67, 75.10, 64.16, 56.96, 56.14, 52.03, 34.49, 34.24, 33.88, 31.73, 31.58, 31.38, 29.54. ESI-MS (+) 1159.487 *m/z* [M+H^+^] (calc.1159.476). IR (ATR-IR) ṽ [cm^−1^] = 3505 (br), 2951 (w), 2942 (w), 2902 (w), 2872 (w), 1645 (s), 1627 (s), 1591 (w), 1548 (w), 1488 (sh), 1450 (s), 1436 (s), 1423 (sh), 1392 (w), 1353 (w), 1330 (w), 1315 (w), 1287 (m), 1256 (s), 1220 (s), 1207 (s), 1196 (s), 1160 (w), 1118 (w), 1083 (m), 1044 (m), 1019 (w), 981 (w), 922 (m), 901 (s), 849 (m), 806 (w), 780 (m), 760 (s), 745 (s), 707 (m), 693 (sh), 665 (w), 645 (w), 634 (w), 613 (w), 577 (w), 544 (w), 521 (m), 509 (m), 450 (sh), 413 (sh). UV-Vis (DCM/MeOH 1:1 *v*/*v*) λ_max_ [nm] = 280, 300, 426 (6726). Raman (100 mW, 1000 scans, 300 K, [cm^−1^]): 2.932 (3), 1.048 (100), 791 (44), 732 (14), 446 (4), 294 (2), 212 (5), 142 (38), 132 (5), 81(81). Elemental analysis calcd. (%) for C_56_H_60_N_2_O_10_U ∙ CH_2_Cl_2_: C 55.03 H 5.02 N 2.25 O 10.29, found: C 55.44 H 4.94 N 2.30 O 11.18.

[(UO_2_)_2_(H_2_L3)(MeO)_2_] (**8**). To the calix[4]arene ligand H_4_L3 (29 mg, 25 µmol, 1 eq.) in a mixed DCM/MeOH solution (1:1 *v*/*v*, 20 mL), neat NEt_3_ (15.4 µL, 111.4 µmol, 4.4 eq.) was added, followed by solid UO_2_(NO_3_)_2_^.^6H_2_O (15 mg, 30 µmol, 2.2 eq.). After the resulting mixture was stirred for 24 h at r.t., the solvent was removed under reduced pressure until a precipitate forms. The resulting suspension was stored at 5 °C for 72 h and filtered using a glass-fritted filter. Redissolving the precipitate in a mixed DCM/MeOH solution (1:2 *v*/*v*, 20 mL) and allowing for slow removal of the solution yields reddish-brown crystals. Yield: 18 mg (42% with regard to H_4_L3). ESI-MS (+) 841.357 *m/z* [H_2_L^2−^ + 2 UO_2_^2+^] (calc. 841.357), 1413.686 *m*/*z* [H_3_L^−^ + UO_2_^2+^] (calc.1413.682), 1681.716 *m/z* [HL^3−^ + 2 UO_2_^2+^] (calc.1681.707), 1713.734 *m*/*z* [M^+–^MeO^−^] (calc.1713.733). IR (ATR-IR) ṽ [cm^−1^] = 3370 (br), 2953 (m), 2902 (sh), 2869 (sh), 1620 (s), 1597 (sh), 1553 (w), 1482 (m), 1467 (m), 1443 (s), 1415 (w), 1392 (w), 1361 (w), 1302 (m), 1243 (s), 1222 (s), 1194 (s), 1171 (sh), 1124 (m), 1103 (w), 1081 (w), 1039 (w), 1023 (w), 971 (w), 901 (s), 870 (m), 817 (w), 781 (w), 736 (m), 642 (w), 587 (w), 570 (w), 542 (w), 524 (w), 441 (w), 420 (w). UV-Vis (DCM/MeOH 1:1 *v*/*v*) λ_max_ [nm] = 280, 292, 356, 400 (4665). The recorded CHN analysis disagrees with the calculated values due to loss of solvent molecules.

## 4. Conclusions

The synthesis and crystallographic characterization of three discrete uranyl complexes supported by pendant calix[4]arene/Schiff-base complexes has been achieved. The vanillylimine-appended calix[4]arene ligands H_4_L1 and H_4_L2 bind to the UO_2_^2+^ ions in **6** and **7** in a pentadentate fashion. Interestingly, only one of the Schiff-base units is in a chelating mode with the UO_2_^2+^ ion. The other ligand arm acts only in a monodentate fashion with the UO_2_^2+^ cation. The calix[4]arene ligand H_4_L3 supports a binuclear, dimethoxido-bridged (UO_2_)(μ-OMe)_2_(UO_2_) core unit. Each ligand arm acts in a tridentate fashion, thereby occupying the terminal coordination sites of the dinuclear uranyl(alkoxido) complex. The calix[4]arene unit acts only as a backbone, with no contacts to the metal atoms, clearly illustrating the pronounced influence of the elongation between calixarene backbone and salicylaldimine residue.

## Figures and Tables

**Table 1:** Selected analytical data for the ligands and uranyl complexes reported in this work.

Compound	ESI-MS(+)/*m*/*z*	ATR-IR/cm^−1^	λ_max_/nm (*ε*/M^−1^cm^−1^) ^[a]^
H_4_L1	1003.58 [H_4_L1+H^+^]	1628 (C=N)	330 (5678), 424 (1716)
H_4_L2	891.46 [H_4_L2+H^+^]	1630 ν(C=N)	337 (5518), 433 (1494)
H_4_L3	1145.66 [H_4_L3+H^+^]	1624 ν(C=N), 1658 ν(C=O_amide_)	337 (5060); 434 (774)
**6**	1271.61 [UO_2_(H_3_L1)]^+^]	1627, 1645 ν(C=N) 901 (ν_as_(O=U=O)	417 (7449)
**7**	1159.49 [UO_2_(H_3_L2)]^+^]	1627, 1645 ν(C=N) 901 ν_as_(O=U=O)	426 (6726)
**8**	1713.73 [(UO_2_)_2_(H_2_L3)(MeO)]^+^	1620 ν(C=O_amide_) 901 (ν_as_(O=U=O)	400 (4665)

^[a]^ Concentration of solutions (DCM/MeOH, *v*/*v* 1:1) were ~ 5.0 × 10^–5^ M, T = 298 K.

**Table 2:** Summary of selected bond lengths [A] and angles [°] for complexes **6**, **7**, and **8**. The listed values only correspond to one representative molecule if multiple complex molecules are located inside the asymmetric unit.

	6	7		8
U-O9	1.779(4)	1.751(18)	U1-O11 (U2-O14)	1.771(8) (1.772(7))
U-O10	1.777(4)	2.045(18)	U1-O12 (U2-O13)	1.795(6) (1.785(7))
U-O1	2.732(3)	2.833(15)	U1-O5 (U2-O8)	2.429(5) (2.415(6))
U-O2	2.211(3)	2.240(1)	U1-N2 (U2-N4)	2.548(7) (2.616(7))
U-O5	2.324(3)	2.249(13)	U1-O6 (U2-O9)	2.239(5) (2.269(5))
U-O7	2.347(3)	2.344(13)	U1-O15 (U2-O15))	2.374(5) (2.348(5))
U-N1	2.579(4)	2.523(18)	U1-O16 (U2-O16)	2.346(6) (2.304(6))
O9-U-O10	176.19(14)	176.2(6)	O11-U1-O12 (O13-U2-O14)	177.0(3) (177.5(3))

## Data Availability

The data supporting this article have been included as part of the ESI. Crystallographic data for compounds H_4_L1, H_4_L2, **6**, **7**, and **8** have been deposited at the CCDC under CCDC-2533613, CCDC-2533614, CCDC-2533616, CCDC-2533615, and CCDC-2533617, respectively. Regarding the DFT, the full workflow, including all scripts, plots, inputs, and outputs generated in this project, are available on Zenodo.

## Supplementary Materials

The following supporting information can be downloaded at: https://www.mdpi.com/article/10.3390/molecules31132357/s1, Figure S1: ^1^H-NMR (400 MHz, 300 K, CDCl_3_) 4-*tert*-Butyl-2-methoxyphenol. Figure S2: ^1^H-NMR (400 MHz, 300 K, CDCl_3_) 5-*tert*-Butyl-2-hydroxy-3-methoxybenzaldehyde. Figure S3: ^1^H-NMR (400 MHz, 300 K, CDCl_3_) Boc-Di-Ala-Calix[4]arene. Figure S4: ^13^C-NMR (100 MHz, 300 K, CDCl_3_) Boc-Di-Ala-Calix[4]arene. Figure S5: COSY ^1^H-^1^H (400 MHz, 300 K, CDCl_3_) Boc-Di-Ala-Calix[4]arene. Figure S6: HSQC ^1^H-^13^C (400–100 MHz, 300 K, CDCl_3_) Boc-Di-Ala-Calix[4]arene. Figure S7: Temp-Var ^1^H-NMR (400 MHz, CDCl_3_) Boc-Di-Ala-Calix[4]arene-15–50 °C. Figure S8: ^1^H-NMR (400 MHz, 300 K, CDCl_3_) Di-Ala-Calix[4]arene (**5**). Figure S9: ^13^C-NMR (100 MHz, 300 K, CDCl_3_) Di-Ala-Calix[4]arene (**5**). Figure S10: COSY ^1^H-^1^H (400 MHz, 300 K, CDCl_3_) Di-Ala-Calix[4]arene (**5**). Figure S11: HSQC ^1^H-^13^C (400–100 MHz, 300 K, CDCl_3_) Di-Ala-Calix[4]arene (**5**). Figure S12: ^1^H-NMR (400 MHz, 300 K, CDCl_3_) H_4_L1. Figure S13: ^13^C-NMR (100 MHz, 300 K, CDCl_3_) H_4_L1. Figure S14: COSY ^1^H-^1^H (400 MHz, 300 K, CDCl_3_) H_4_L1. Figure S15: HSQC ^1^H-^13^C (400-100 MHz, 300 K, CDCl_3_) H_4_L1. Figure S16: ^1^H-NMR (400 MHz, 300 K, CDCl_3_) H_4_L2. Figure S17: ^13^C-NMR (100 MHz, 300 K, CDCl_3_) H_4_L2. Figure S18: COSY ^1^H-^1^H (400 MHz, 300 K, CDCl_3_) H_4_L2. Figure S19: NOESY ^1^H-^1^H (400 MHz, 300 K, CDCl_3_) H_4_L2. Figure S20: HSQC ^1^H-^13^C (400-100 MHz, 300 K, CDCl_3_) H_4_L2. Figure S21: ^1^H-NMR (400 MHz, 300 K, CDCl_3_) H_4_L3. Figure S22: ^13^C-NMR (100 MHz, 300 K, CDCl_3_) H_4_L3. Figure S23: COSY ^1^H-^1^H (400 MHz, 300 K, CDCl_3_) H_4_L3. Figure S24: HSQC ^1^H-^13^C (400–100 MHz, 300 K, CDCl_3_) H_4_L3. Figure S25: ^1^H-NMR (400 MHz, 300 K, CD_2_Cl_2_) [(UO_2_)(H_2_L1)] (**6**). Figure S26: ^13^C-NMR (100 MHz, 300 K, CD_2_Cl_2_) [(UO_2_)(H_2_L1)] (**6**). Figure S27: COSY ^1^H-^1^H (400 MHz, 300 K, CD_2_Cl_2_) [(UO_2_)(H_2_L1)] (**6**). Figure S28: HSQC-^1^H-^13^C (400–100 MHz, 300 K, CD_2_Cl_2_) [(UO_2_)(H_2_L1)] (**6**). Figure S29: ^1^H-NMR (400 MHz, 300 K, CD_2_Cl_2_) [(UO_2_)(H_2_L2)] (**7**). Figure S30: ^13^C-NMR (100 MHz, 300 K, CD_2_Cl_2_) [(UO_2_)(H_2_L2)] (**7**). Figure S31: COSY ^1^H-^1^H (400 MHz, 300 K, CD_2_Cl_2_) [(UO_2_)(H_2_L2)] (**7**). Figure S32: HSQC ^1^H-^13^C (400–100 MHz, 300 K, CD_2_Cl_2_) [(UO_2_)(H_2_L2)] (**7**). Figure S33: ^1^H-NMR (400 MHz, 300 K, CD_2_Cl_2_) [(UO_2_)_2_(H_2_L3)(MeO)_2_] (**8**). Figure S34: ATR-IR Boc-Di-Ala-Calix[4]arene. Figure S35: ATR-IR Di-Ala-Calix[4]arene (5). Figure S36: ATR-IR H_4_L1. Figure S37: ATR-IR H_4_L2. Figure S38: ATR-IR H_4_L3. Figure S39: ATR-IR [(UO_2_)(H_2_L1)] (**6**). Figure S40: ATR-IR [(UO_2_)(H_2_L2)] (**7**). Figure S41: ATR-IR [(UO_2_)_2_(H_2_L3)(MeO)2] (**8**). Figure S42: UV-vis spectrum of H_4_L1 recorded in DCM/MeOH (c = 5.0 × 10^–5^ M). Figure S43: UV-vis spectrum of H_4_L2 recorded in DCM/MeOH (c = 5.0 × 10^–5^ M). Figure S44: UV-vis spectrum of H_4_L3 recorded in DCM/MeOH (c = 5.0 × 10^–5^ M). Figure S45: UV-vis spectrum of [(UO_2_)(H_2_L1)] (**6**). recorded in DCM/MeOH (c = 5.0 × 10^–5^ M). Figure S46: UV-vis spectrum of [(UO_2_)(H_2_L2)] (**7**). recorded in DCM/MeOH (c = 5.0 × 10^–5^ M). Figure S47: UV-vis spectrum of [(UO_2_)_2_(H_2_L3)(MeO)2] (**8**). recorded in DCM/MeOH (c = 5.0 × 10^–5^ M). Figure S48: Raman spectrum of complex [UO_2_(H_2_L2)] (**7**). Figure S49: ESI(+)-MS of Boc-Di-Ala-Calix[4]arene. Figure S50: Expended region of the ESI(+)-MS of Boc-Di-Ala-Calix[4]arene. Figure S51: Expended region of the ESI(+)-MS of Di-Ala-Calix[4]arene (5). Figure S52: Expended region of the ESI(+)-MS of Di-Ala-Calix[4]arene (5). Figure S53: ESI(+)-MS of H_4_L1. Figure S54: Expended region of the ESI(+)-MS of H_4_L1. Figure S55: ESI(+)-MS of H_4_L2. Figure S56 Expended region of the ESI(+)-MS of H_4_L2. Figure S57: ESI(+)-MS of H_4_L3. Figure S58: Expended region of the ESI(+)-MS of H_4_L3. Figure S59: ESI(+)-MS of [(UO_2_)(H_2_L1)] (**6**). Figure S60: Expended region of the ESI(+)-MS of [(UO_2_)(H_2_L1)] (**6**). Figure S61: ESI(+)-MS of [(UO_2_)(H_2_L2)] (**7**). Figure S62: Expended region of the ESI(+)-MS of [(UO_2_)(H_2_L2)] (**7**). Figure S63: ESI(+)-MS of [(UO_2_)_2_(H_2_L3)(MeO)_2_] (**8**). Figure S64: Excerpt of the ESI(+)-MS of [(UO_2_)_2_(H_2_L3)(MeO)_2_] (**8**). Figure S65: Excerpt of the ESI(+)-MS of [(UO_2_)_2_(H_2_L3)(MeO)_2_] (**8**). Figure S66: Excerpt of the ESI(+)-MS of [(UO_2_)_2_(H_2_L3)(MeO)_2_] (**8**). Figure S67: Excerpt of the ESI(+)-MS of [(UO_2_)_2_(H_2_L3)(MeO)_2_] (**8**). Figure S68: The resulting fingerprint plot for the calculated NCI plot of [(UO_2_)(H_2_L1)] (**6**). Figure S69: Fingerprint plot for the calculated NCI plot for [(UO_2_)_2_(H_2_L3)(MeO)_2_] (**8**). Figure S70: Color change upon UO_2_(NO_3_)_2_ * 6 H_2_O addition to a solution of the Ligand H_4_L2 in DCM / MeOH with 4.4 eq. NEt_3_. Figure S71: ORTEP representation of H_4_L1. Thermal ellipsoids drawn at 50% probability. Color code: C, grey; H, white; N, light blue; O, red. Solvent molecules have been omitted for clarity. Figure S72: ORTEP representation of H_4_L2. Thermal ellipsoids drawn at 50% probability. Color code: C, grey; H, white; N, light blue; O, red. Solvent molecules have been omitted for clarity. Figure S73: ORTEP representation of [(UO_2_)(H_2_L1)] (**6**). Thermal ellipsoids drawn at 50% probability. Color code: C, grey; H, white; N, light blue; O, red; U. deep blue. Solvent molecules have been omitted for clarity. Figure S74: ORTEP representation of [(UO_2_)(H_2_L2)] (**7**). Thermal ellipsoids drawn at 50% probability. Color code: C, grey; H, white; N, light blue; O, red; U. deep blue. Solvent molecules have been omitted for clarity. Figure S75: ORTEP representation of [(UO_2_)_2_(H_2_L3)(MeO)_2_] (**8**). Thermal ellipsoids drawn at 50% probability. Color code: C, grey; H, white; N, light blue; O, red; U. deep blue. Solvent molecules have been omitted for clarity. Table S1: Crystal data and structure refinement for the reported structures. Table S2: xyz coordinates of the crystal structure and the dft optimized structure of **6**. Table S3: xyz coordinates of the crystal structure and the dft calculated structure of **8**. Figure S76: Overlayed calculated (pink) and experimentally determined (blue) molecular structure of complex **6**. Figure S77: Overlayed calculated (pink) and experimentally determined (blue) molecular structure of complex **8**.
