# Supplementary material for: Synthesis, Characterization, and Molecular Structure of Some Uranyl Complexes Supported by Hybrid Salicylaldimine/Calix[4]arene Ligands
Source: Molecules. 2026 Jul 3;31(13):2357. doi: 10.3390/molecules31132357 (PMC13362544; doi:10.3390/molecules31132357)
Supplement: Supplementary file 1 [file molecules-31-02357-s001.zip › molecules-4398289-supplementary.pdf]

Supporting Information

Synthesis, Characterization, and Molecular Structure of some Uranyl Complexes supported by Hybrid Salicylaldimine/Calix[4]arene Ligands

Table of Contents

1. Synthesis 2
2. Spectra 3
3. Single Crystal X-Ray crystallography 38
4. Comparison of optimized and experimentally determined structures of 6 and 8 42
References 51

1. Synthesis

Synthesis of the Boc-Ala-OPfp-ester was performed according to the reported procedure by GREEN *et.al.*.[1] 5-*tert*-Butyl-2-hydroxy-3-methoxybenzaldehyde was obtained by following a modified literature procedure, with a detailed description given below.[2]

4-*tert*-butyl-2-methoxyphenol

2.75 g (12.1 mmol) 2-Bromo-4-*tert*-butylphenol as well as 831.3 mg (6.18 mmol) anhydrous $CuCl_2$ were dissolved in 100 ml of DMF before adding a freshly prepared sodium methoxide solution (prep. by dissolving 5.01 g Na in 10 ml MeOH). The resulting mixture was heated to reflux for 1.5 h, filtered and the solvent partially evaporated under reduced pressure. The remaining solution was poured into water and 1 M HCl was added until a neutral pH was archived. The mixture was extracted 3 x with 100 ml DCM. The combined organic extracts were washed with 150 ml of a saturated NaCl solution, dried using Na_2SO_4 and concentrated under reduced pressure. Column chromatography on silica gel (DCM as eluent) yielded 4-*tert*-butyl-2-methoxyphenol as an orange oil.

Yield: 1.44 g (7.98 mmol, 66%).

^{1}H-NMR (400 MHz, $CDCl_3$) δ [ppm] = 6.87 (m, 3H, ArH); 5.46 (s, 1H, -OH); 3.90 (s, 3H, O-CH_3); 1.31 (s, 9H, $C(CH_3)_3$).

5-*tert*-Butyl-2-hydroxy-3-methoxybenzaldehyde

710 mg (3.94 mmol) 4-*tert*-butyl-2-methoxyphenol and 5.52 g (39.37 mmol) of Hexamethylenetetramine were dissolved in 25 ml of Trifluoroacetic acid and heated to reflux for 16 h. The resulting mixture was then hydrolyzed over the course of 24 h using 50 ml of 1 M HCl. The resulting mixture was extracted 3 times with 100 ml of DCM. The combined organic layers were washed with water, dried using $MgSO_4$ and the solvent was removed

under reduced pressure. The resulting red oil was used for the following reactions without additional purification.

Yield: 573 mg (crude, 2.75 mmol, 70%).

^{1}H-NMR (400 MHz, $CDCl_3$) δ [ppm] = 9.91 (s, 1H, Ar-CHO); 7.17 (d, 1H, ArH, J = 2.2 Hz); 7.14 (d, 1H, J = 2.2 Hz, ArH); 3.93 (s, 3H, Ar-OCH_3), 1.34 (s, 9H, Ar-$C(CH_3)$)).

2. Spectra

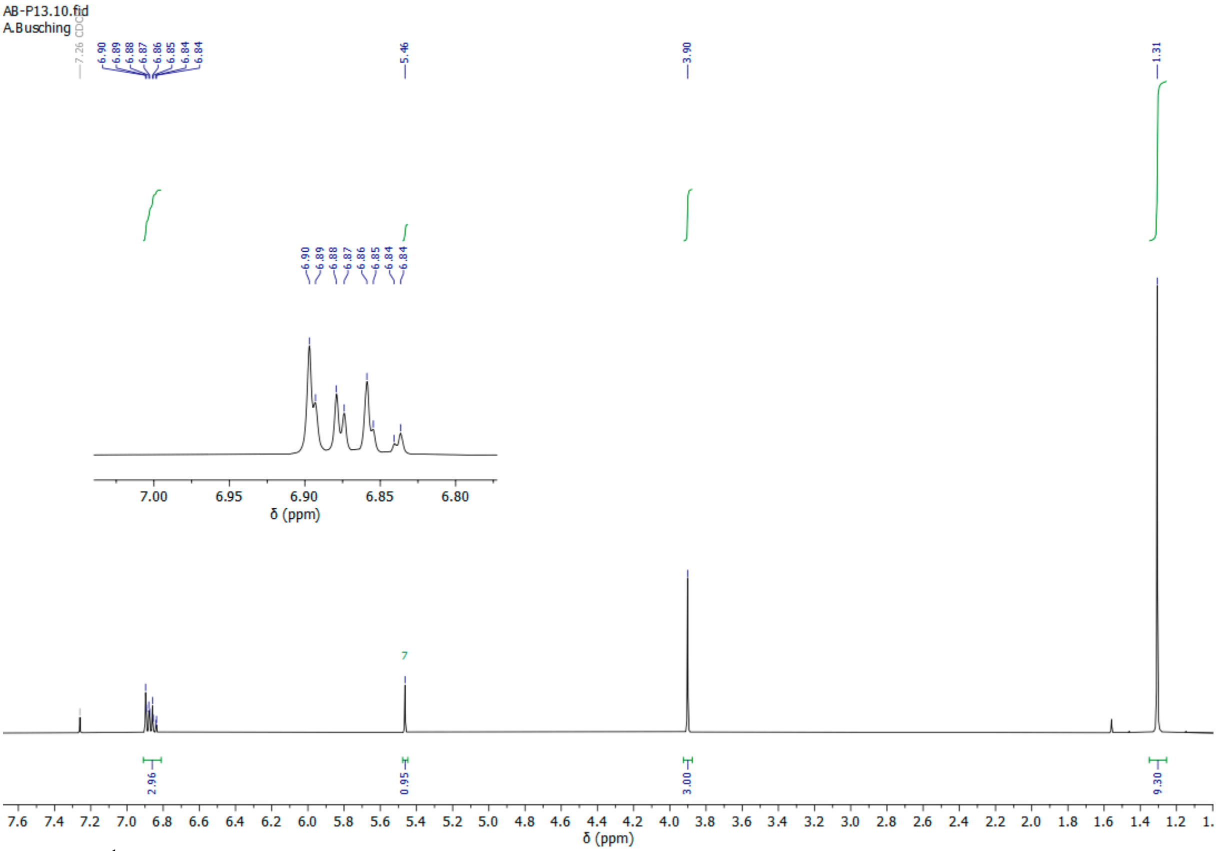

Figure S1: ^{1}H-NMR (400 MHz, 300 K, $CDCl_3$) 4-*tert*-Butyl-2-methoxyphenol.

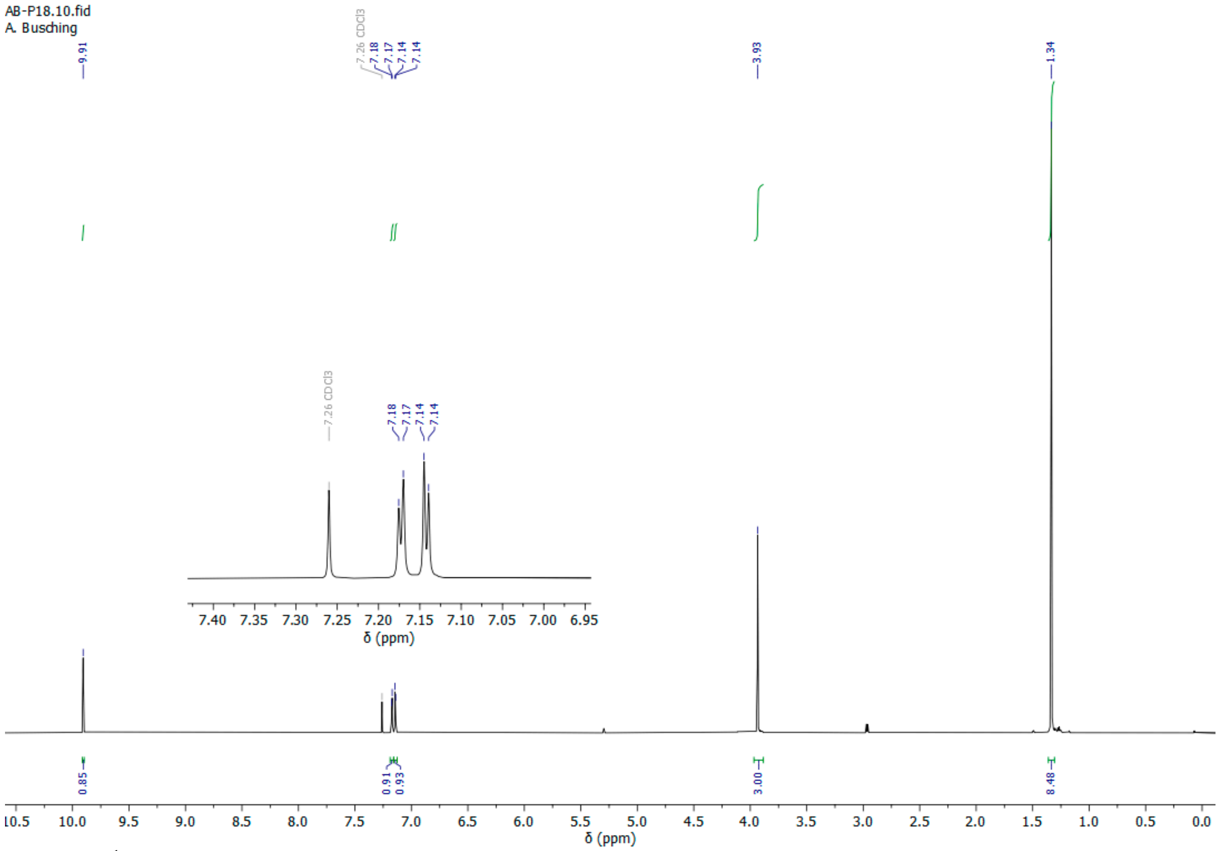

Figure S2: ^{1}H-NMR (400 MHz, 300 K, $CDCl_3$) 5-*tert*-Butyl-2-hydroxy-3-methoxybenzaldehyde.

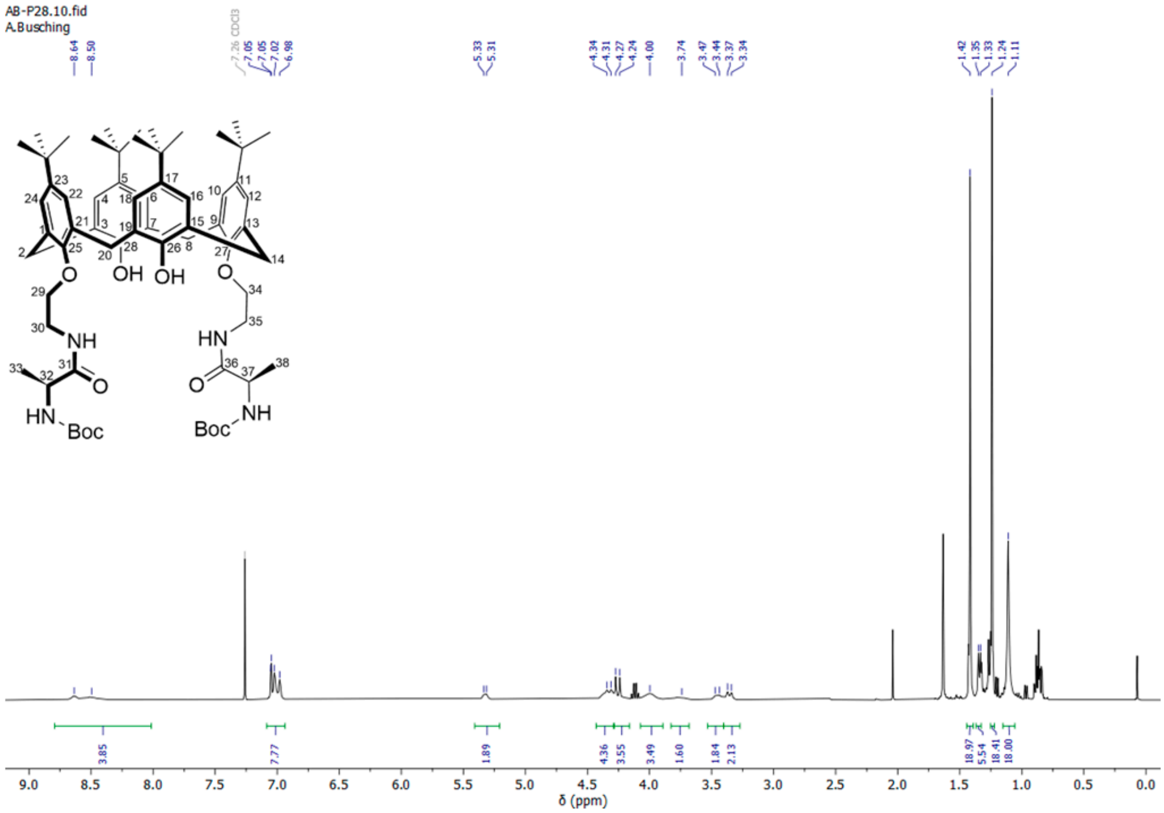

Figure S3: ^{1}H-NMR (400 MHz, 300 K, $CDCl_3$) Boc-Di-Ala-Calix[4]arene.

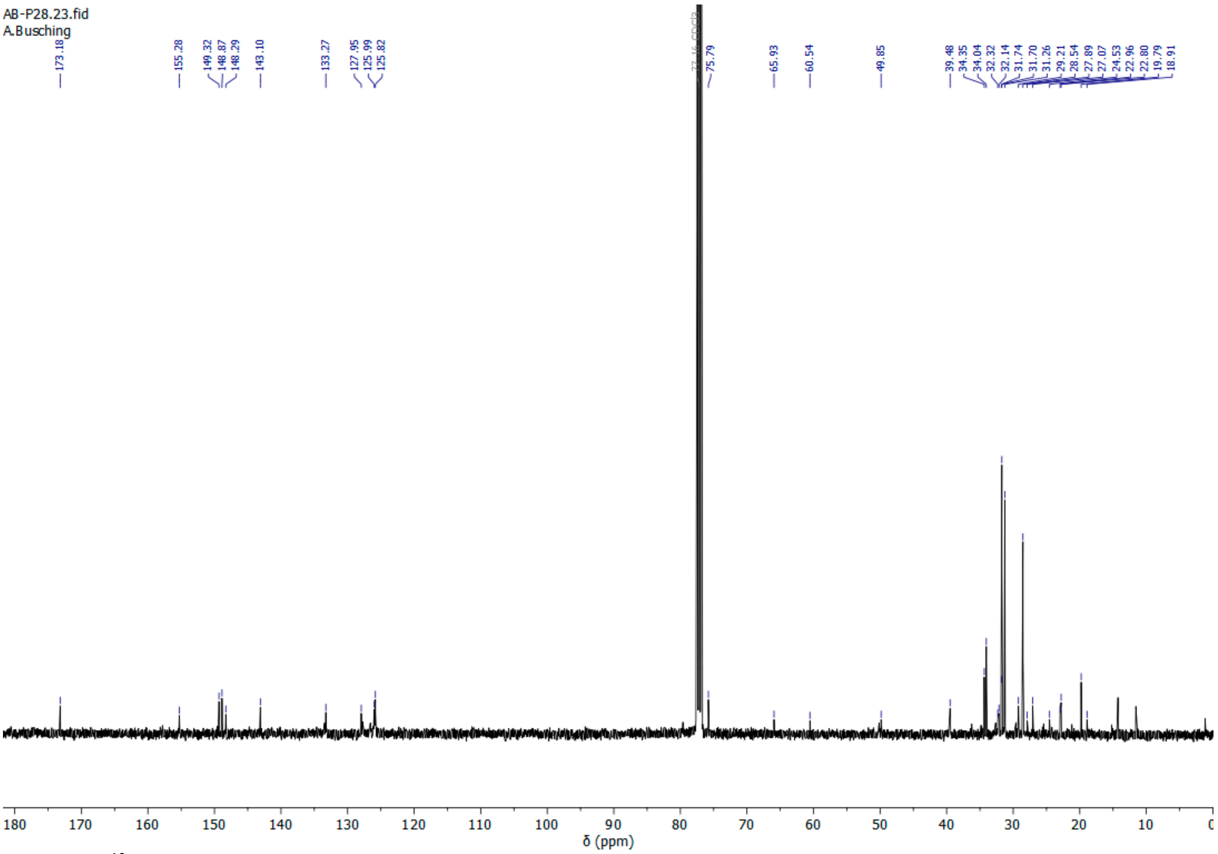

Figure S4: ^{13}C-NMR (100 MHz, 300 K, $CDCl_3$) Boc-Di-Ala-Calix[4]arene.

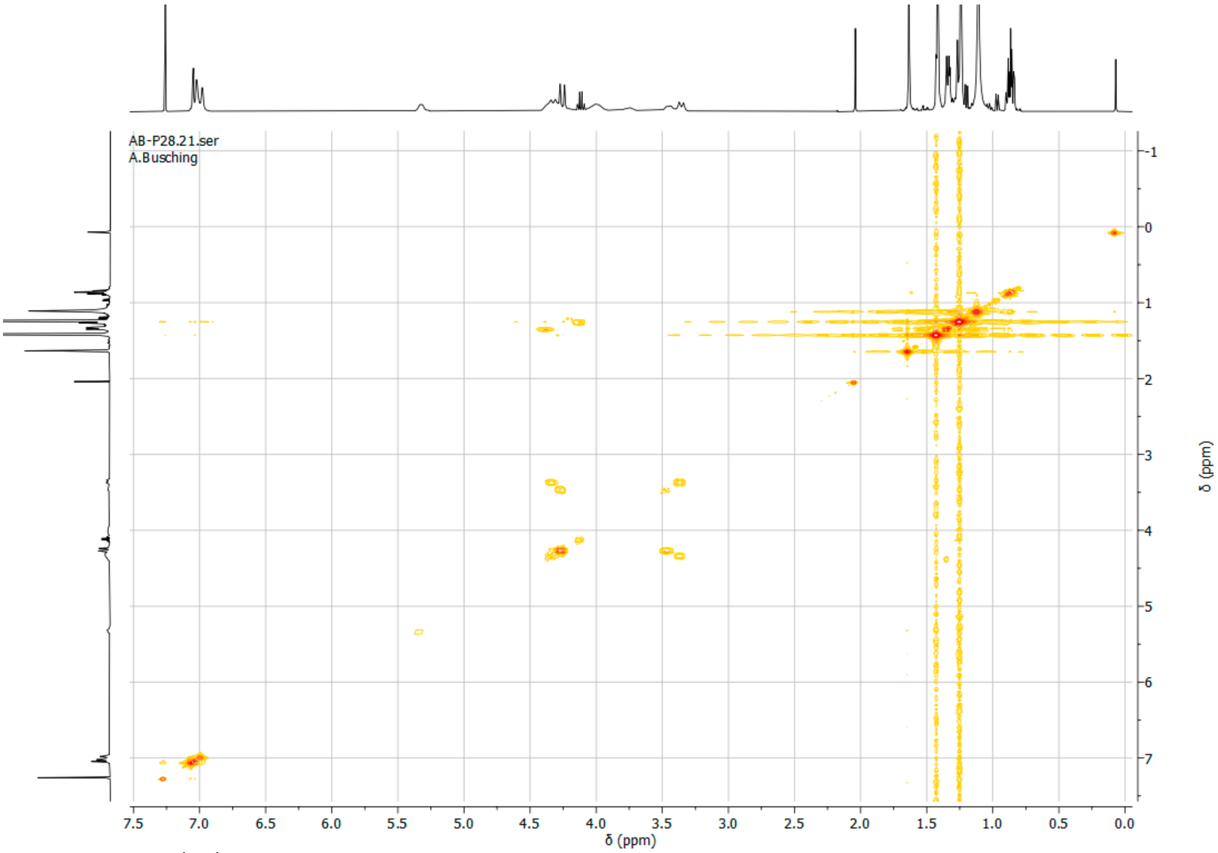

Figure S5: COSY ^{1}H-^{1}H (400 MHz, 300 K, $CDCl_3$) Boc-Di-Ala-Calix[4]arene.

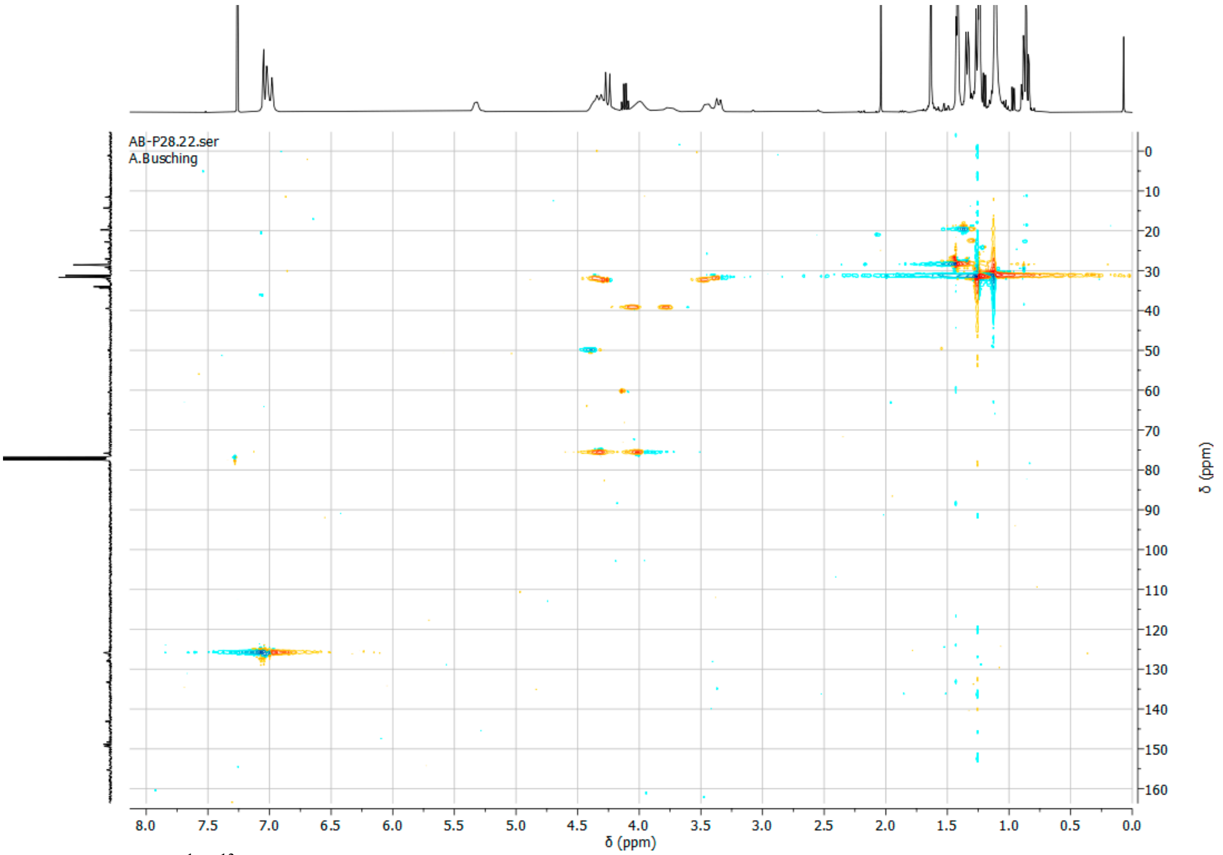

Figure S6: HSQC ^{1}H-^{13}C (400-100 MHz, 300 K, $CDCl_3$) Boc-Di-Ala-Calix[4]arene.

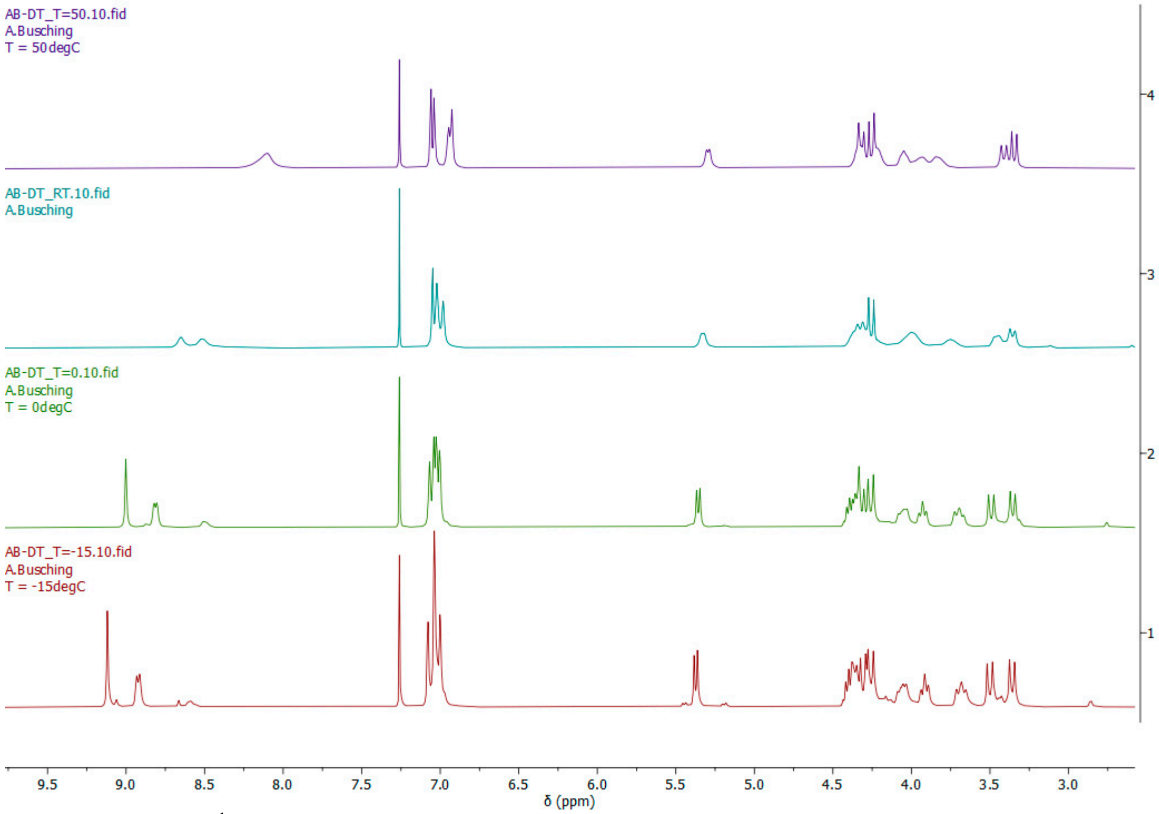

Figure S7: Temp-Var ^{1}H-NMR (400 MHz, $CDCl_3$) Boc-Di-Ala-Calix[4]arene -15 – 50 °C.

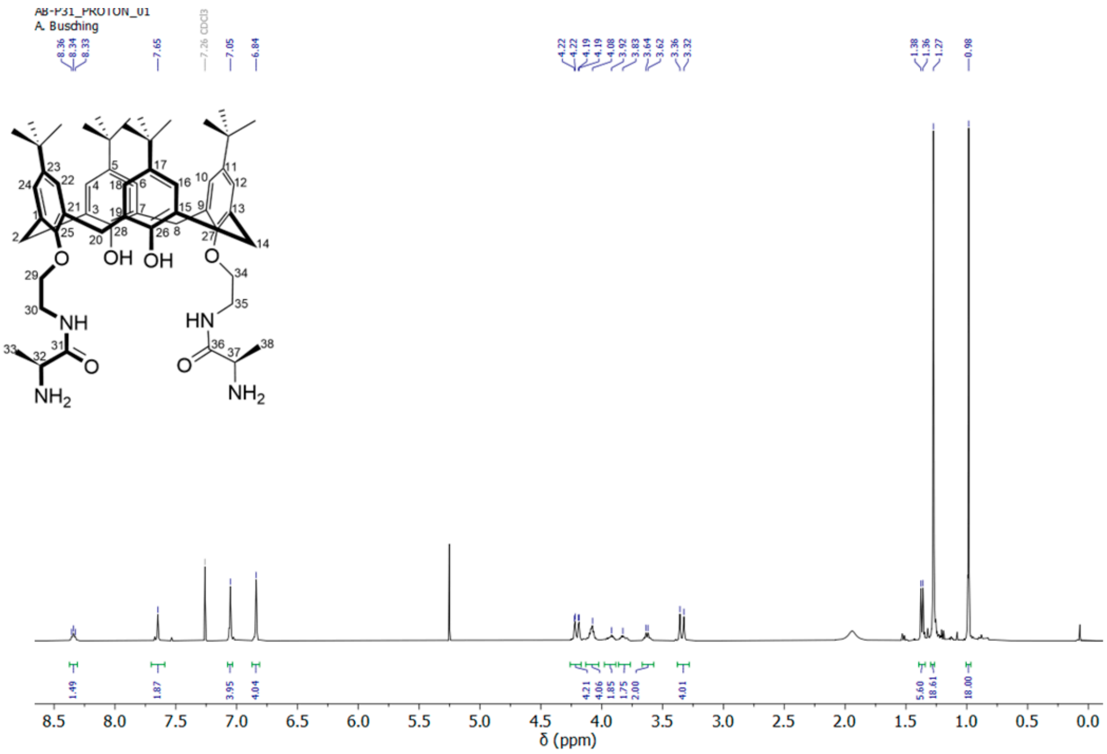

Figure S8: ^{1}H-NMR (400 MHz, 300 K, $CDCl_3$) Di-Ala-Calix[4]arene (**5**).

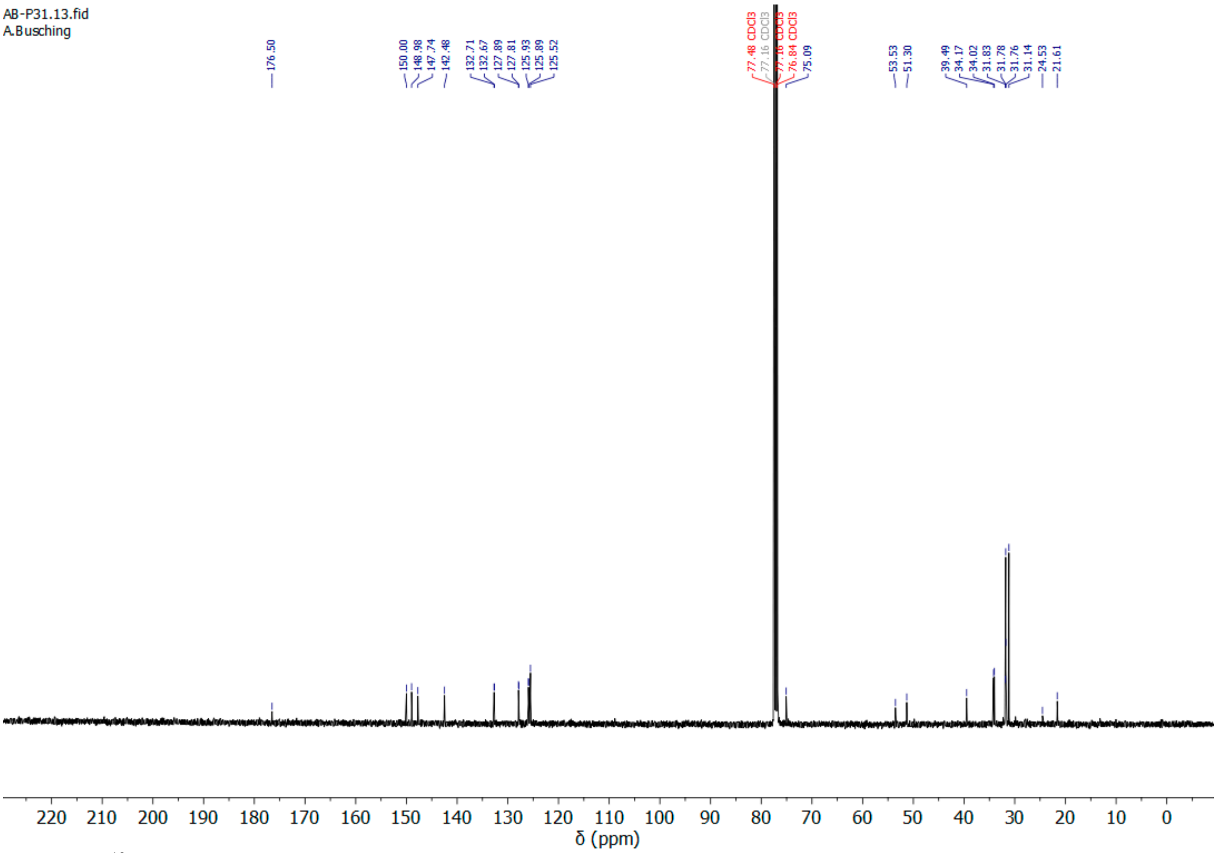

Figure S9: ^{13}C-NMR (100 MHz, 300 K, $CDCl_3$) Di-Ala-Calix[4]arene (**5**).

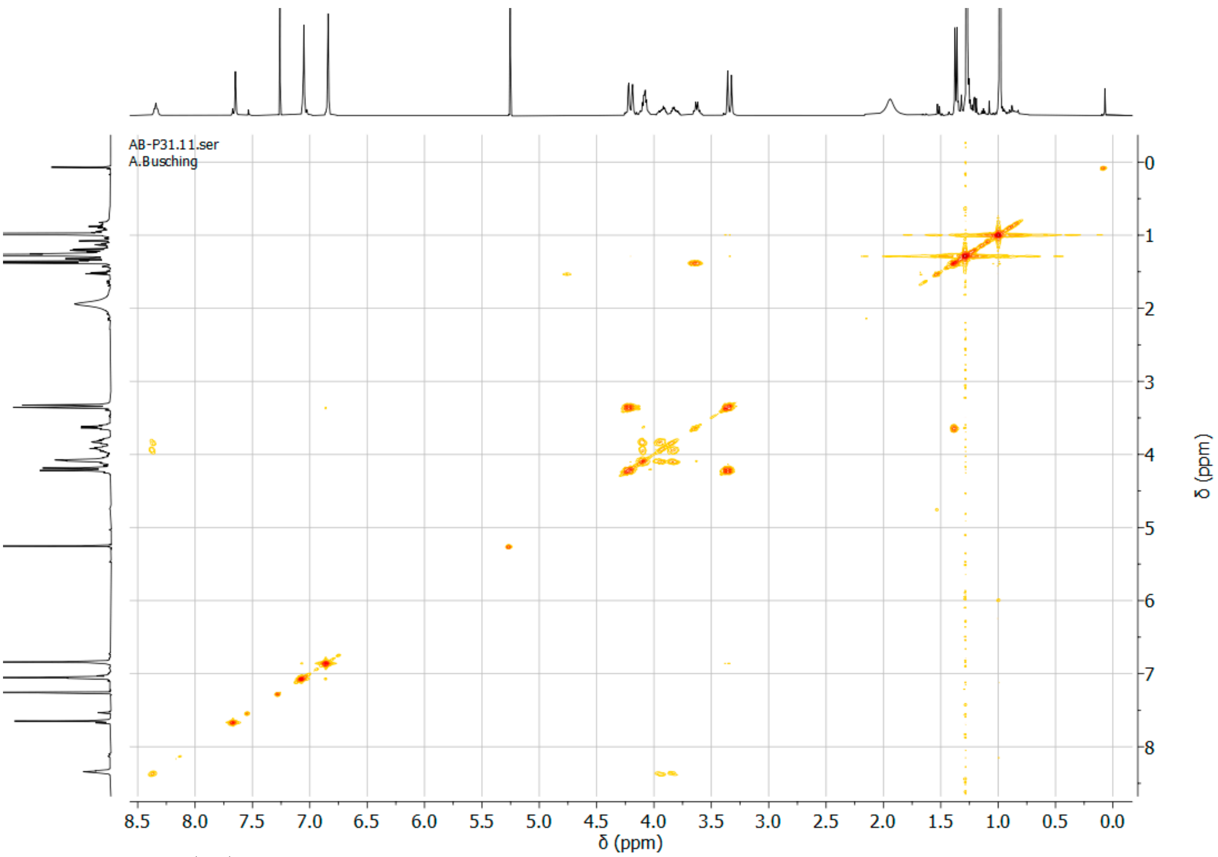

Figure S10: COSY ^{1}H-^{1}H (400 MHz, 300 K, $CDCl_3$) Di-Ala-Calix[4]arene (**5**).

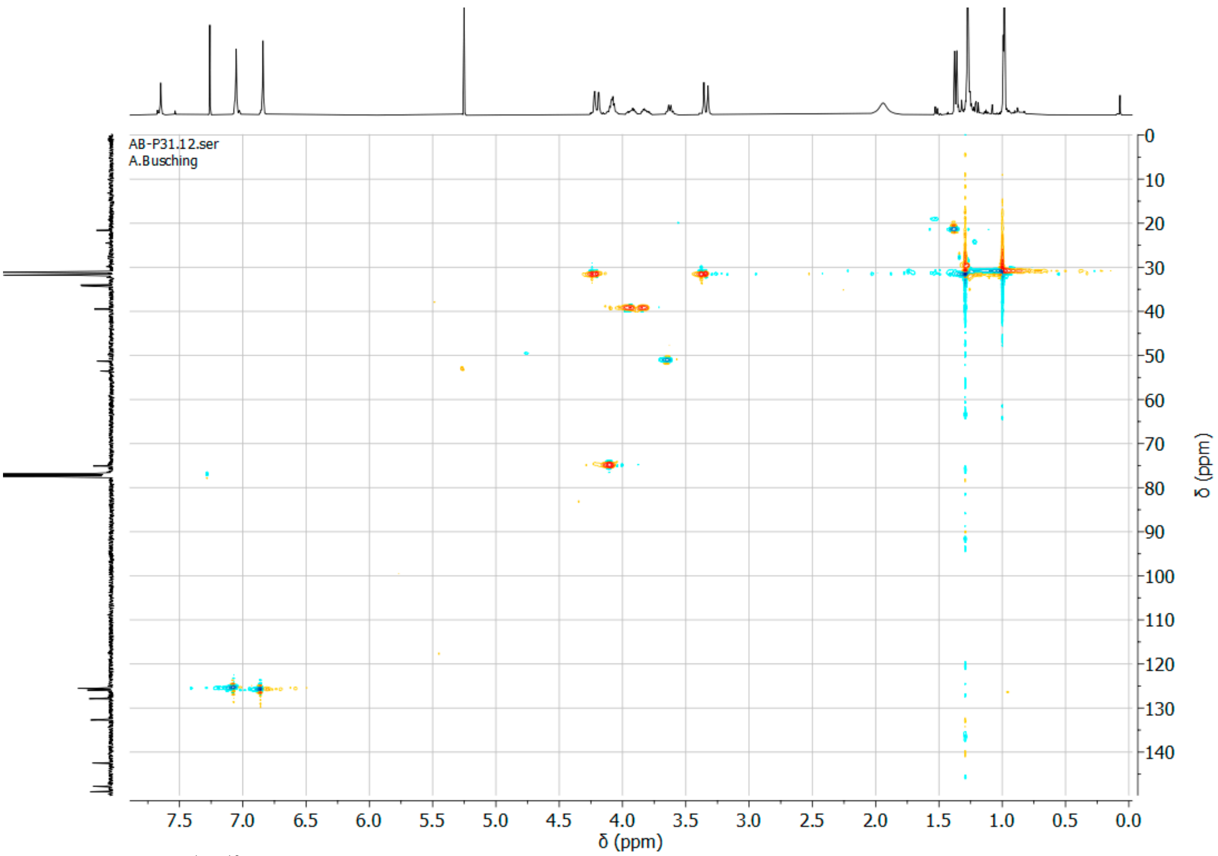

Figure S11: HSQC ^{1}H-^{13}C (400-100 MHz, 300 K, $CDCl_3$) Di-Ala-Calix[4]arene (**5**).

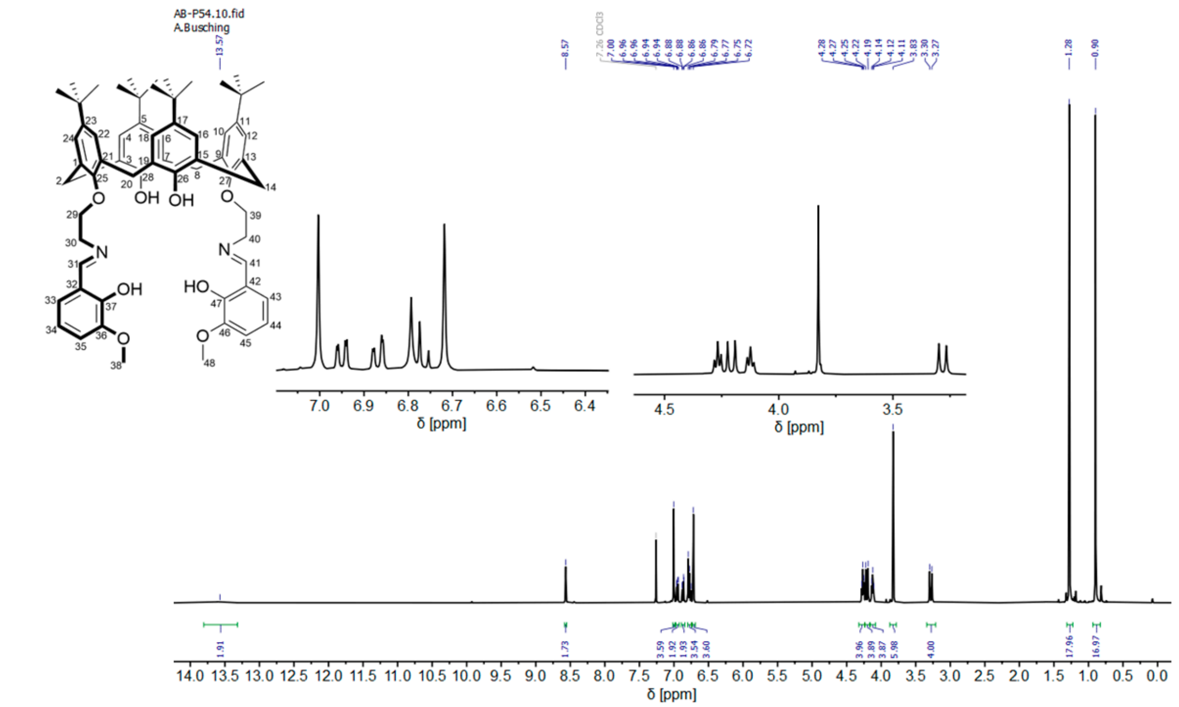

Figure S12: ^{1}H-NMR (400 MHz, 300 K, $CDCl_3$) H_4L1.

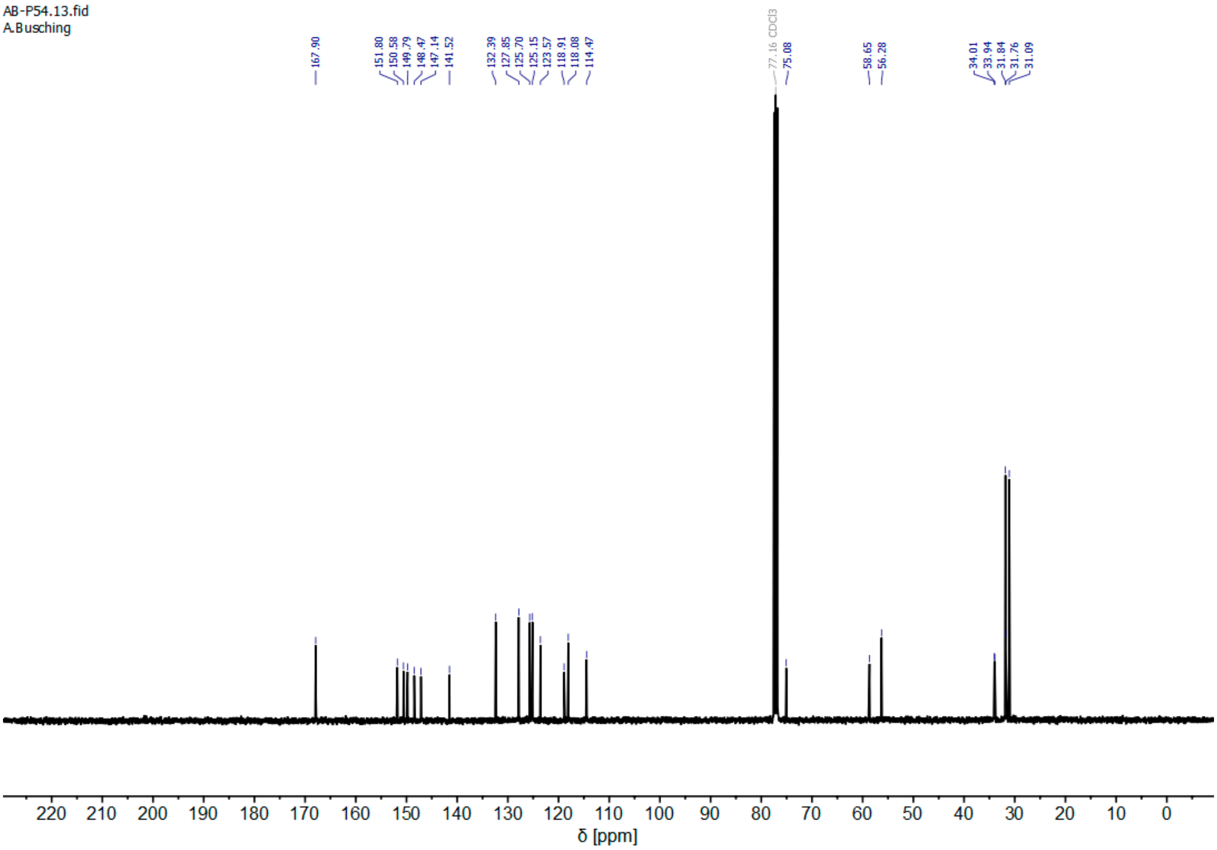

Figure S13: ^{13}C-NMR (100 MHz, 300 K, $CDCl_3$) H_4L1.

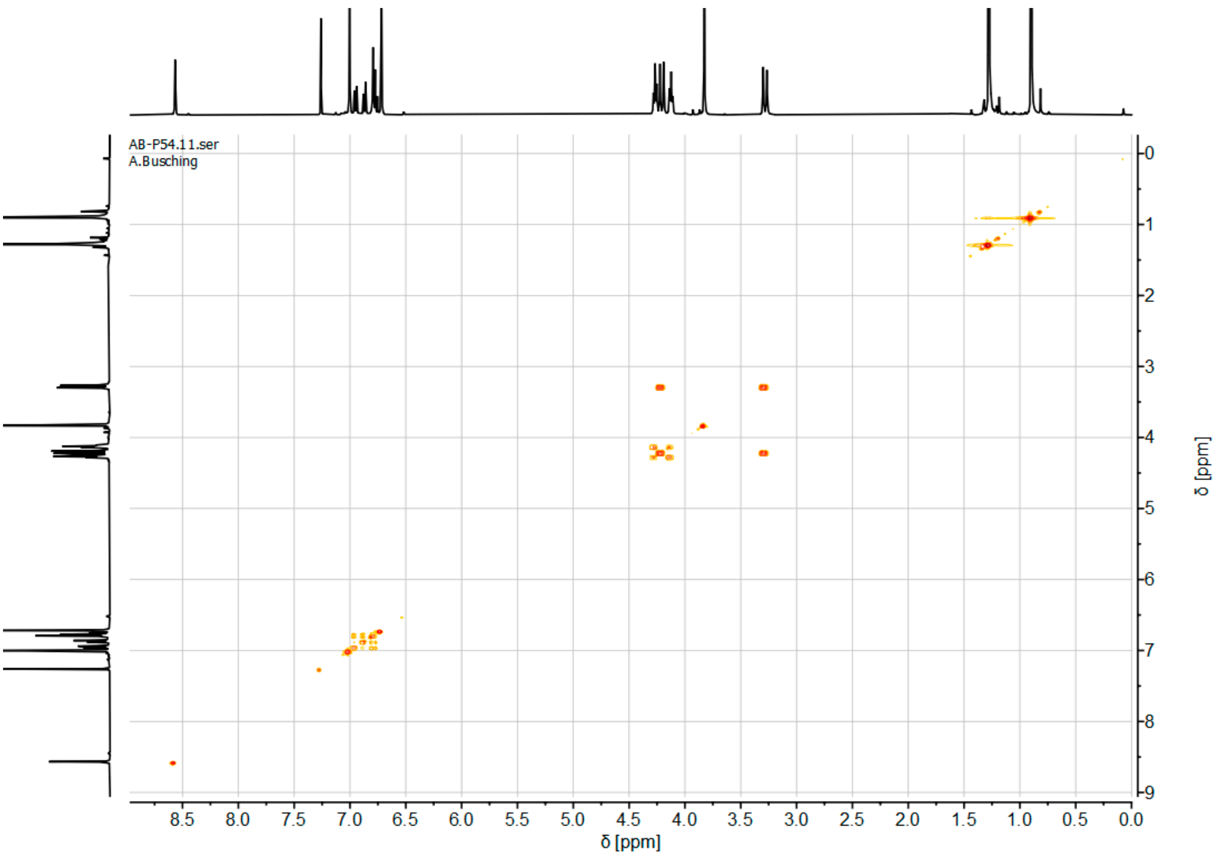

Figure S14: COSY ^{1}H-^{1}H (400 MHz, 300 K, $CDCl_3$) H_4L1.

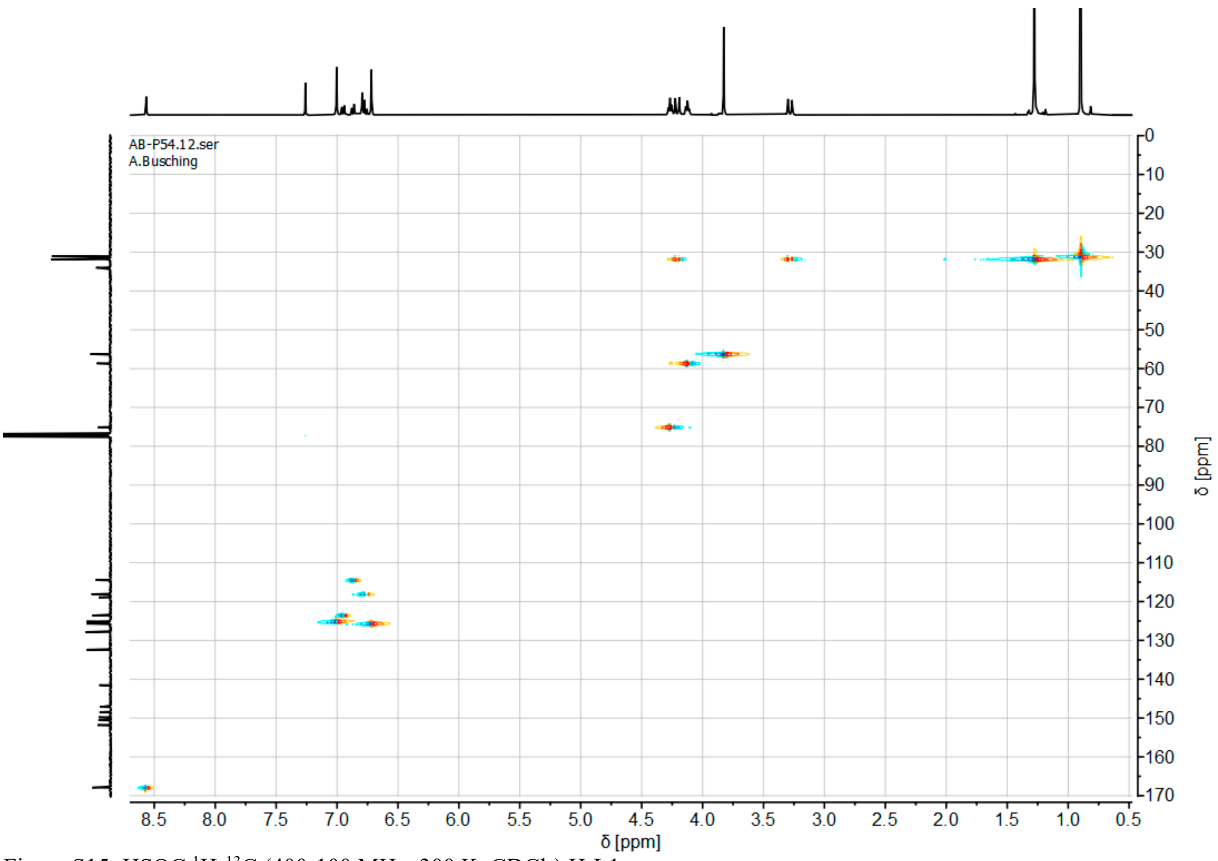

Figure S15: HSQC ^{1}H-^{13}C (400-100 MHz, 300 K, $CDCl_3$) H_4L1.

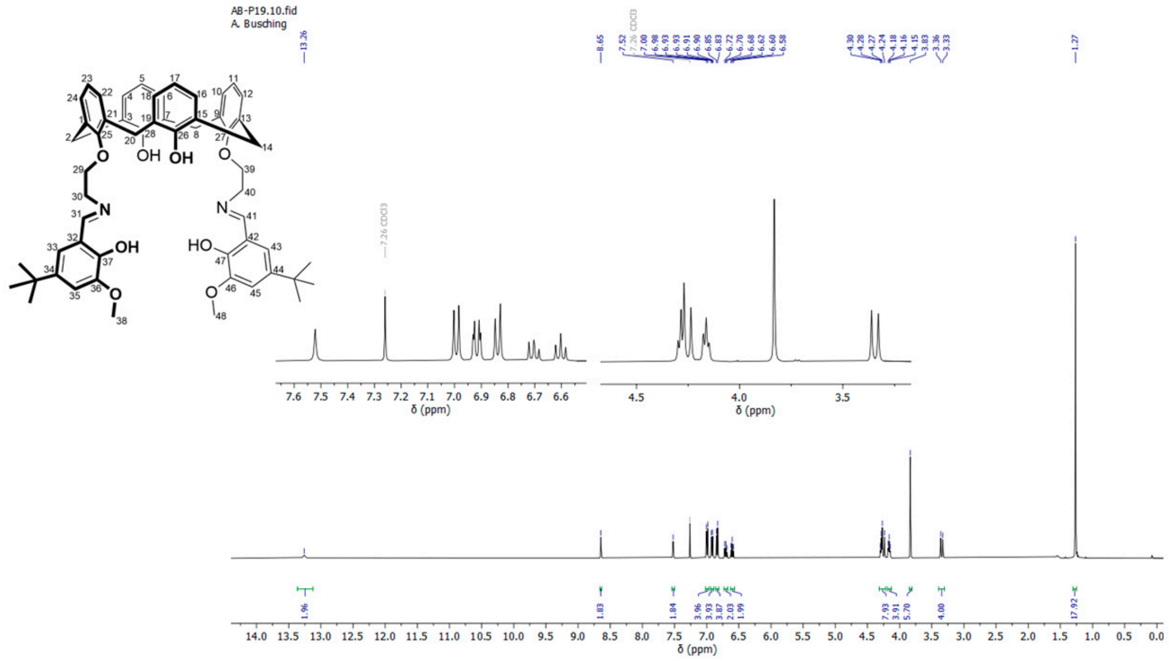

Figure S16: ^{1}H-NMR (400 MHz, 300 K, $CDCl_3$) H_4L2.

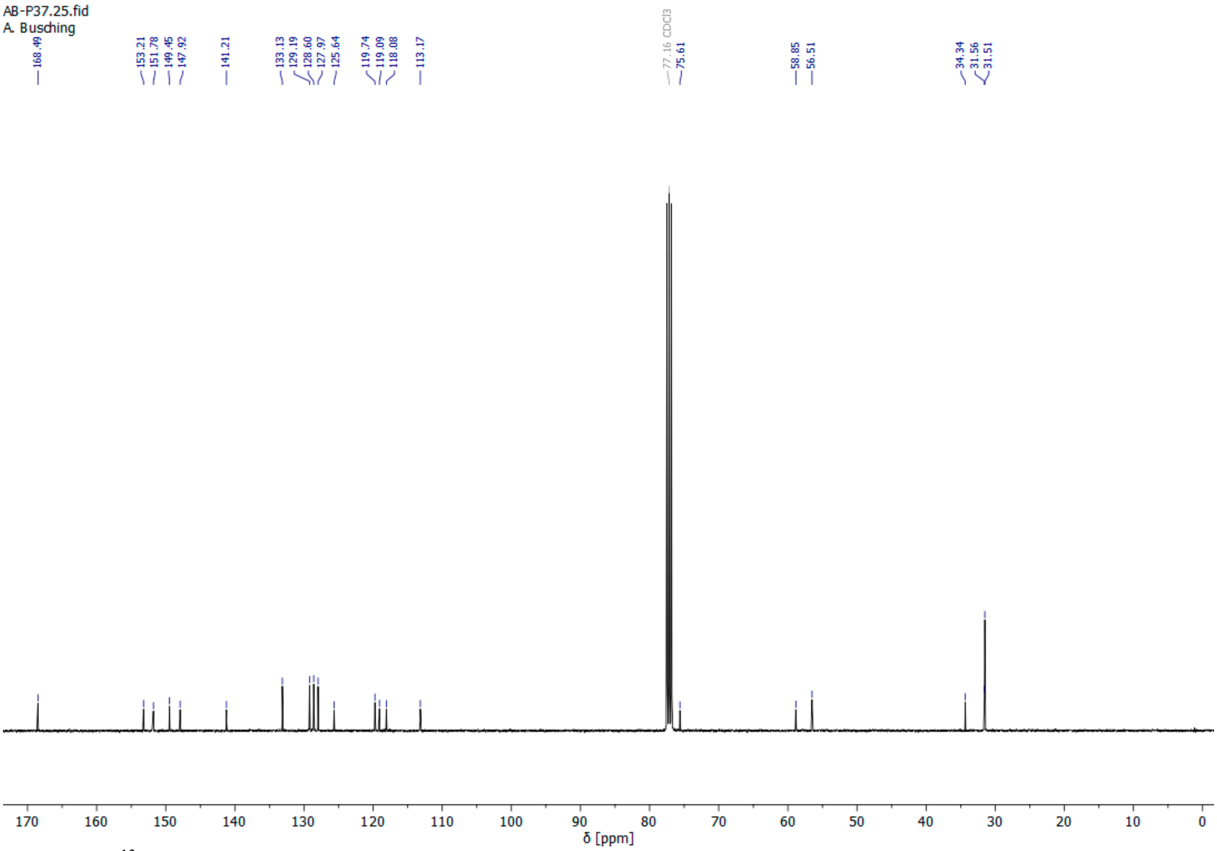

Figure S17: ^{13}C-NMR (100 MHz, 300 K, $CDCl_3$) H_4L2.

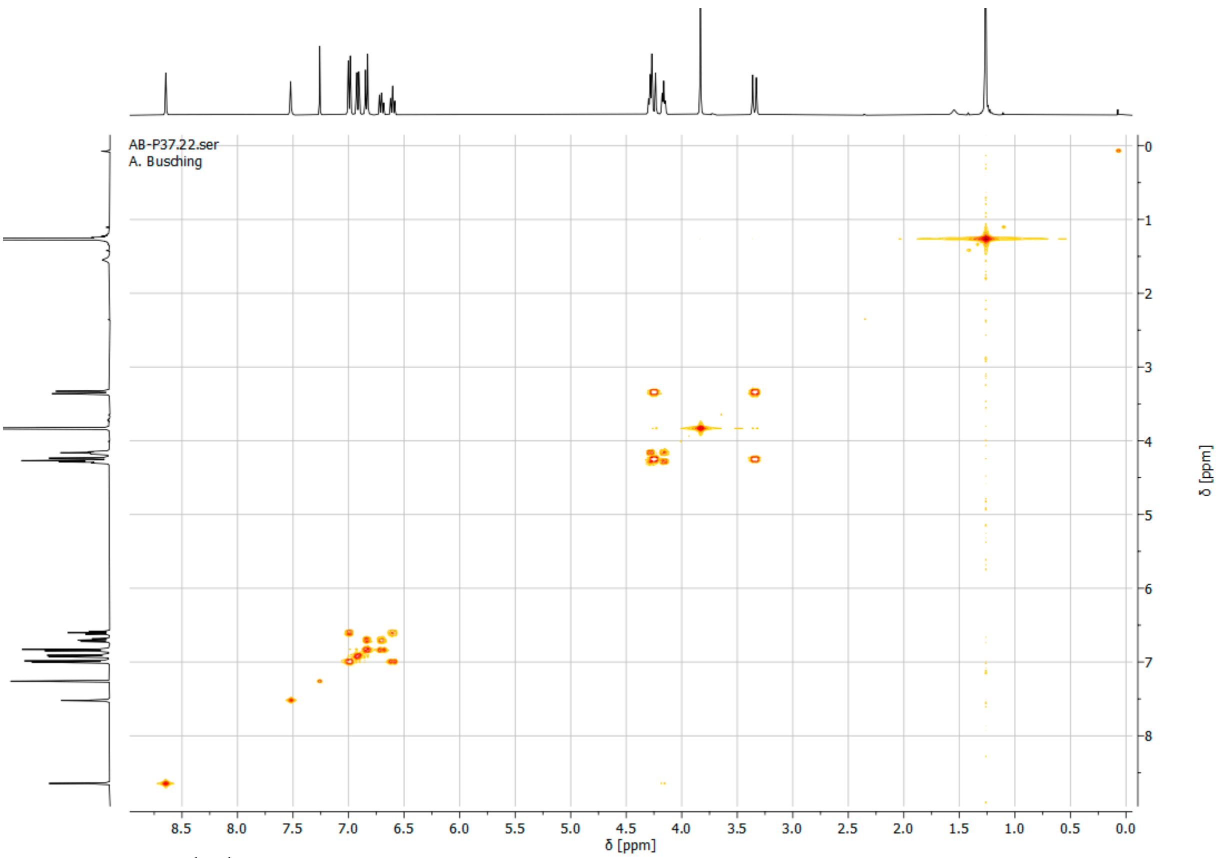

Figure S18: COSY ^{1}H-^{1}H (400 MHz, 300 K, $CDCl_3$) H_4L2.

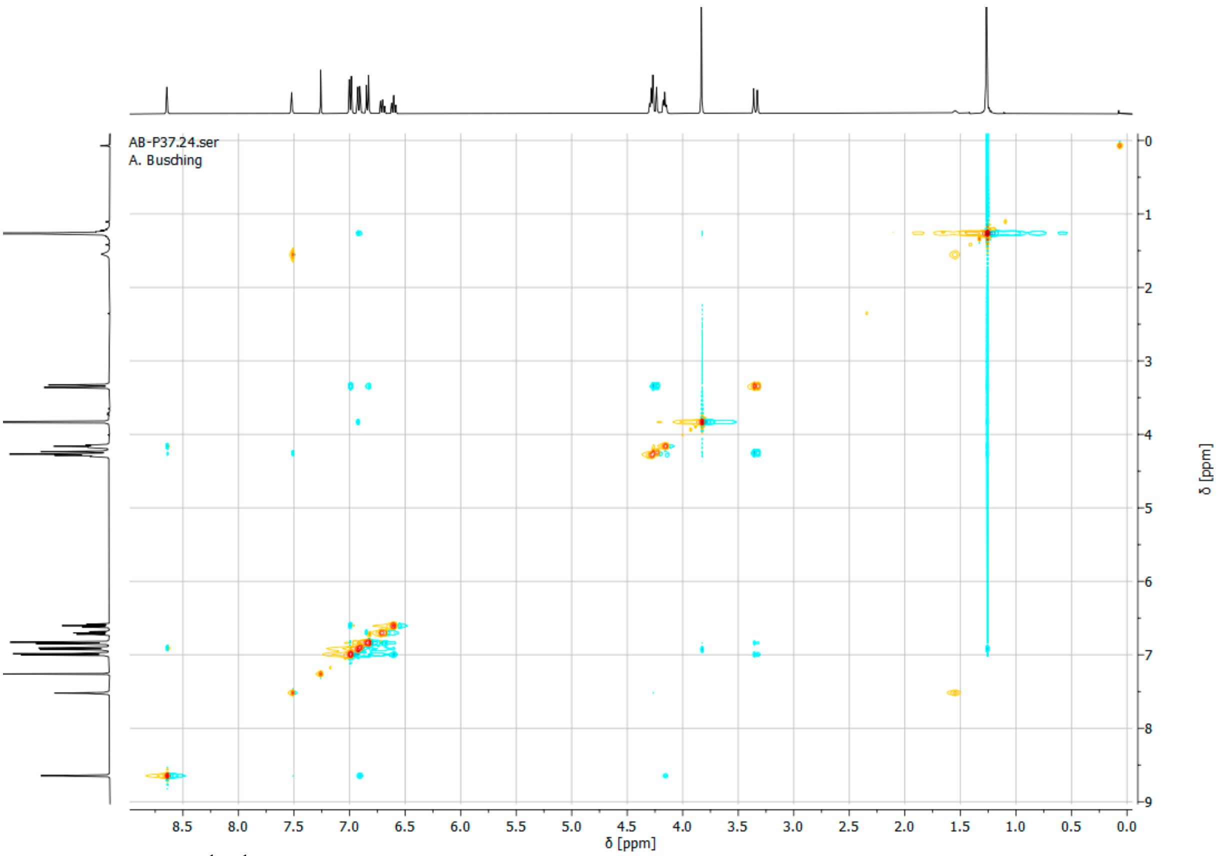

Figure S19: NOESY ^{1}H-^{1}H (400 MHz, 300 K, $CDCl_3$) H_4L2.

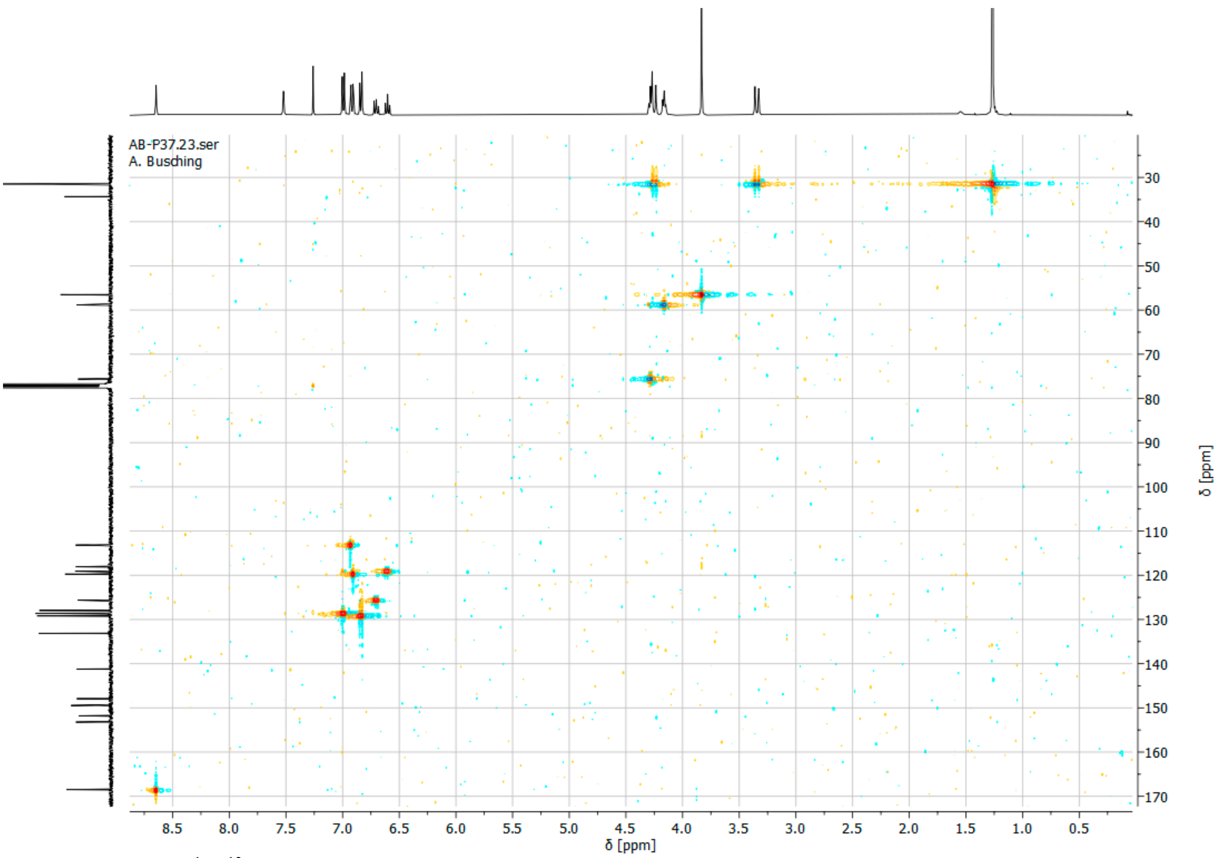

Figure S20: HSQC ^{1}H-^{13}C (400-100 MHz, 300 K, $CDCl_3$) H_4L2.

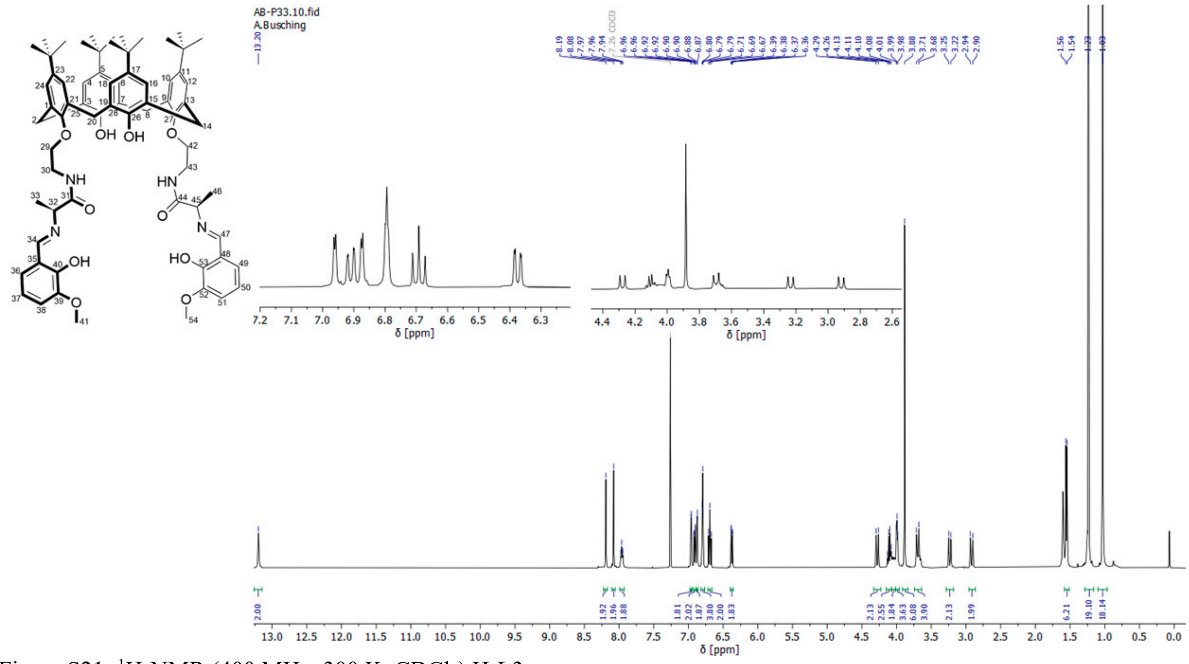

Figure S21: ^{1}H-NMR (400 MHz, 300 K, $CDCl_3$) H_4L3.

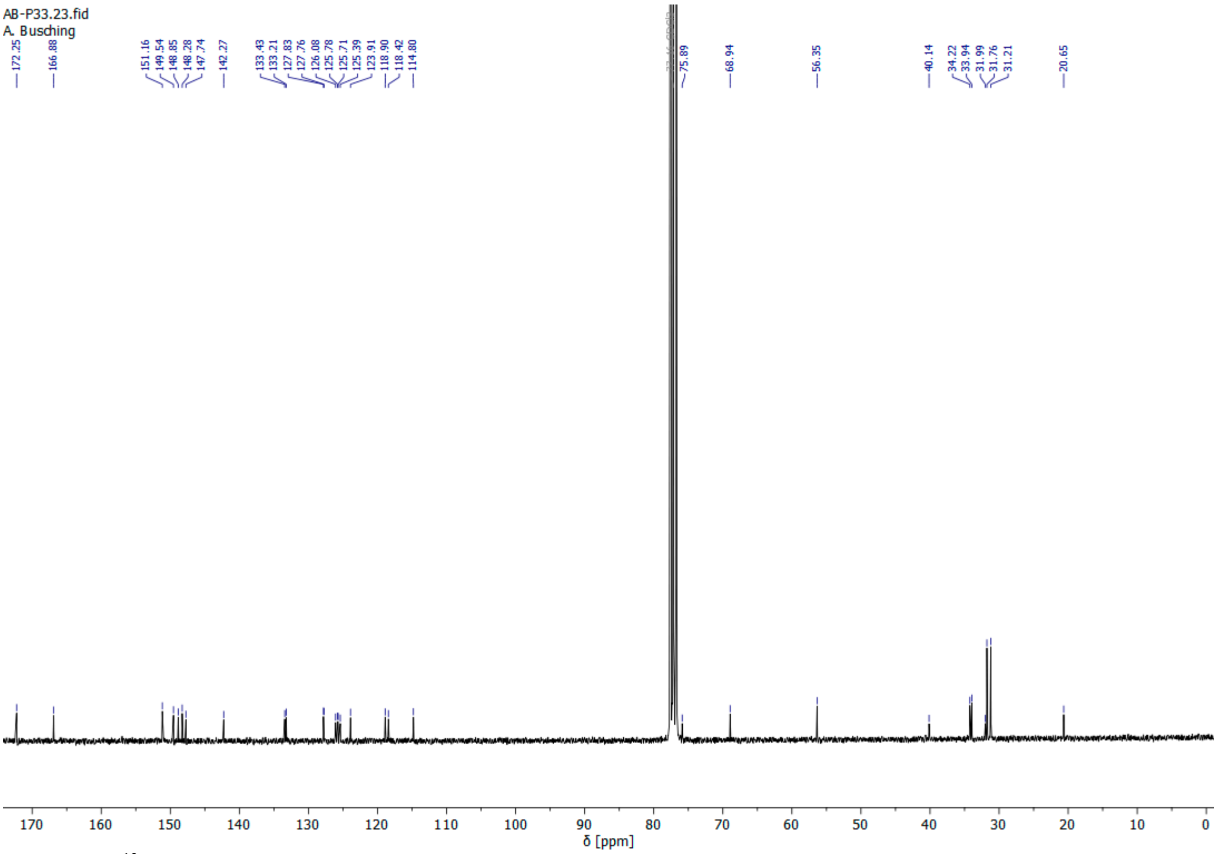

Figure S22: ^{13}C-NMR (100 MHz, 300 K, $CDCl_3$) H_4L3.

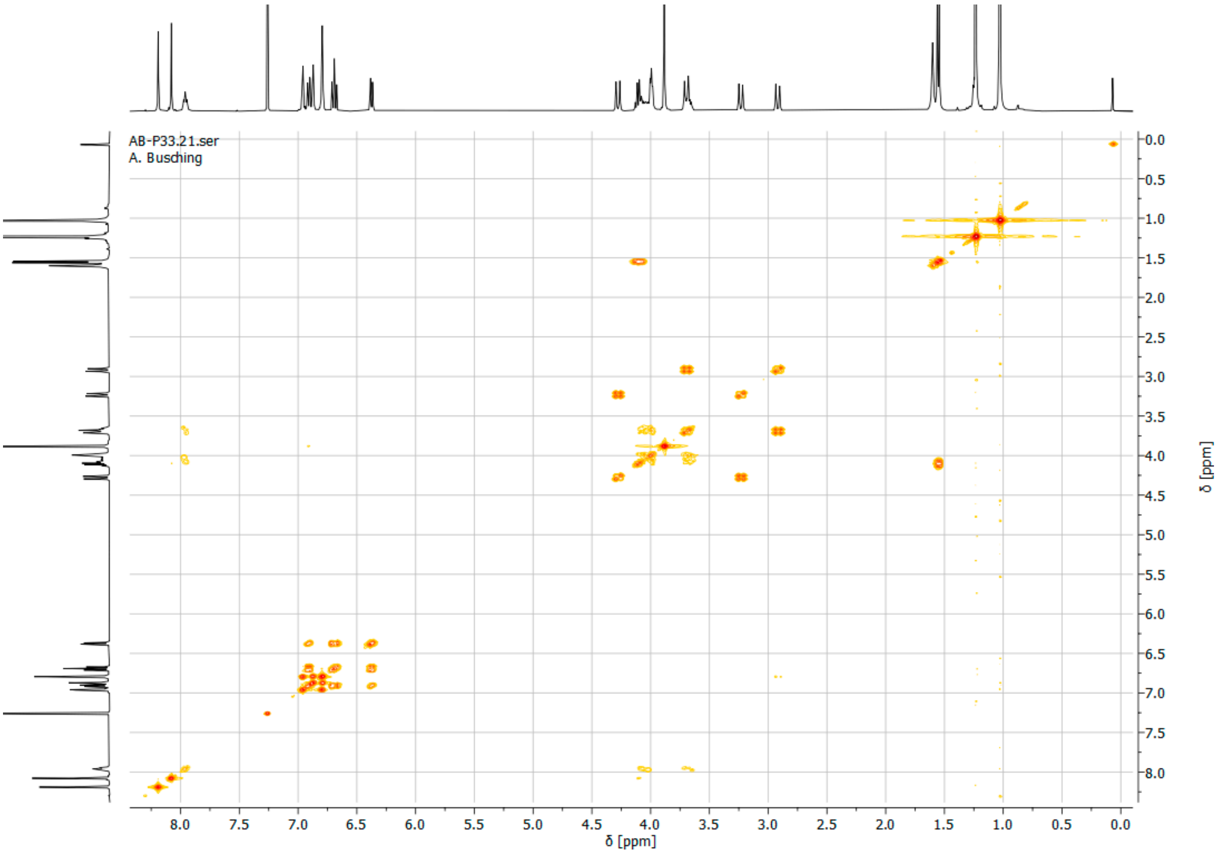

Figure S23: COSY ^{1}H-^{1}H (400 MHz, 300 K, $CDCl_3$) H_4L3.

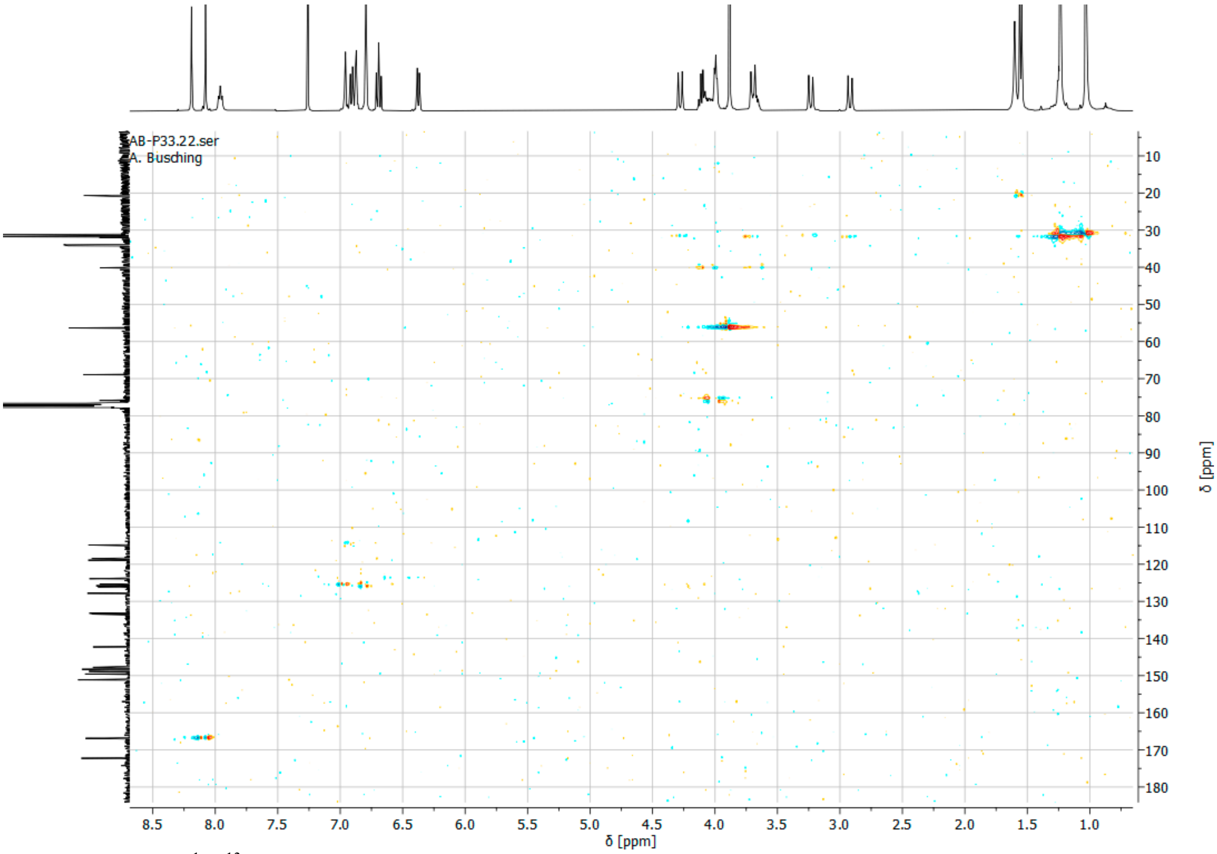

Figure S24: HSQC ^{1}H-^{13}C (400-100 MHz, 300 K, $CDCl_3$) H_4L3.

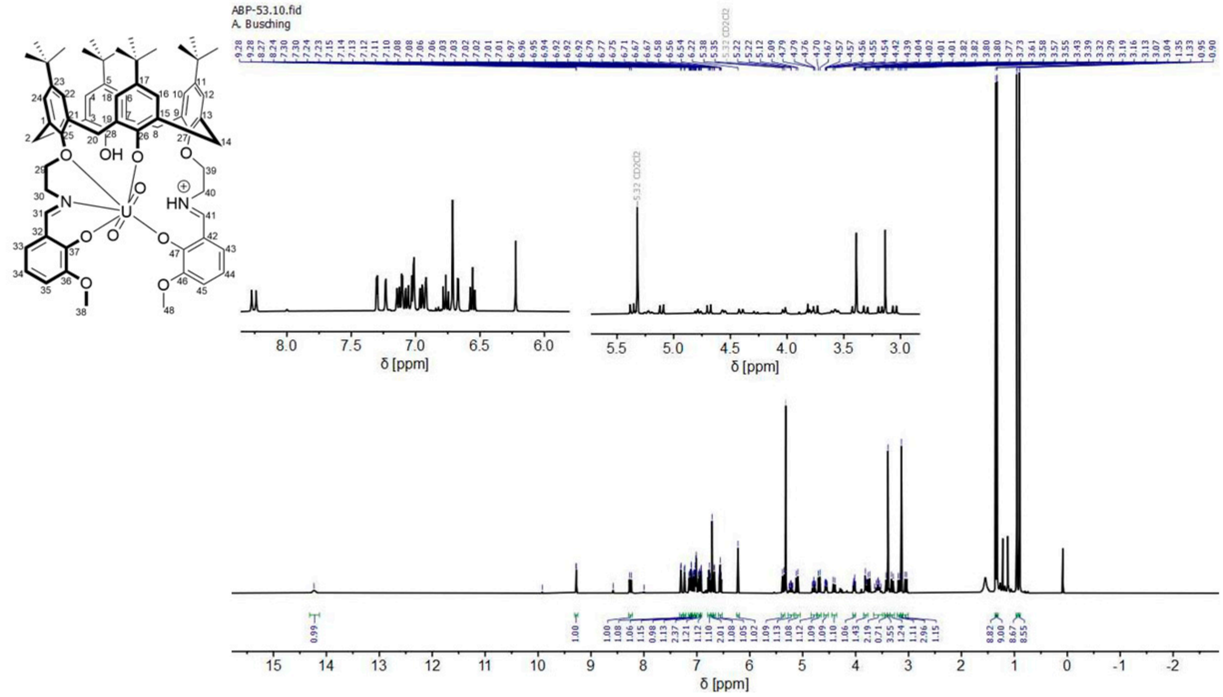

Figure S25: ^{1}H-NMR (400 MHz, 300 K, CD_2Cl_2) $[(UO_2)(H_2L1)]$ (**6**).

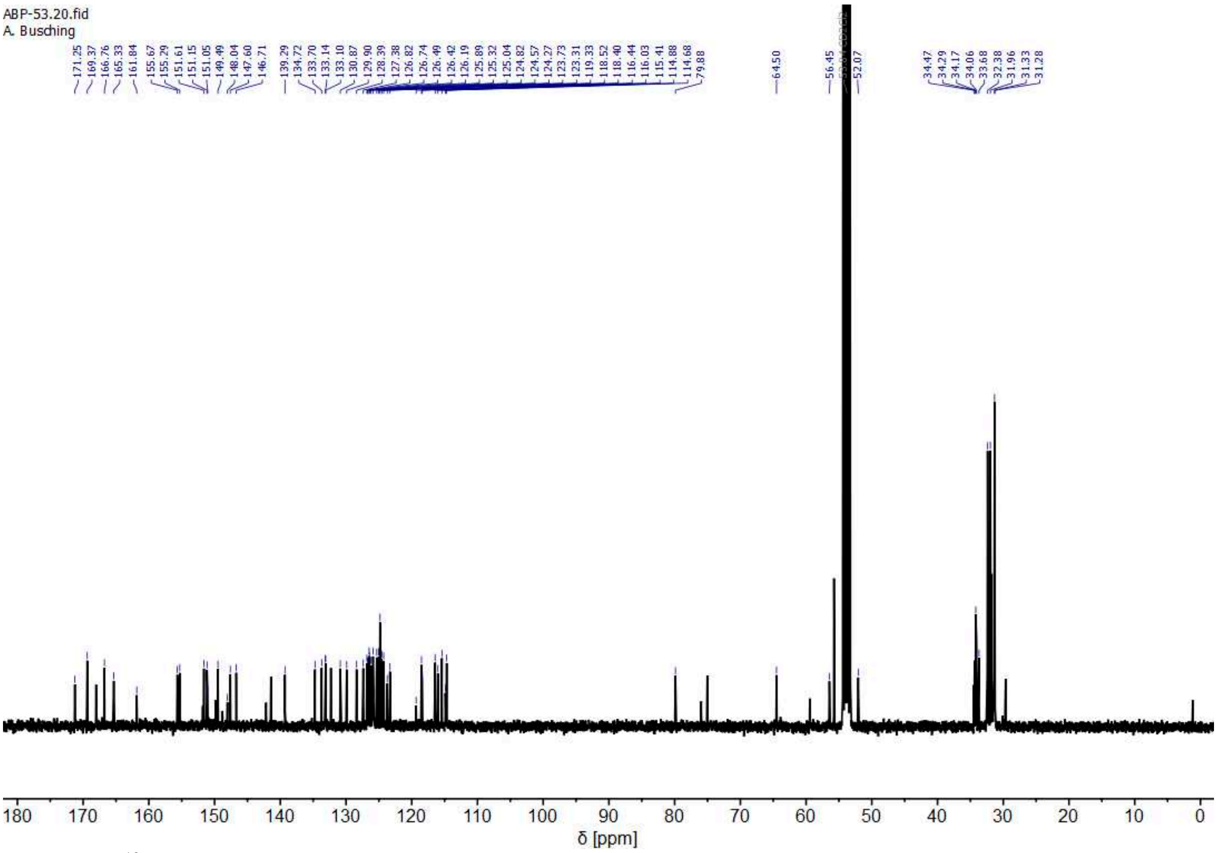

Figure S26: ^{13}C-NMR (100 MHz, 300 K, CD_2Cl_2) [$(UO_2)(H_2L1)$] (**6**).

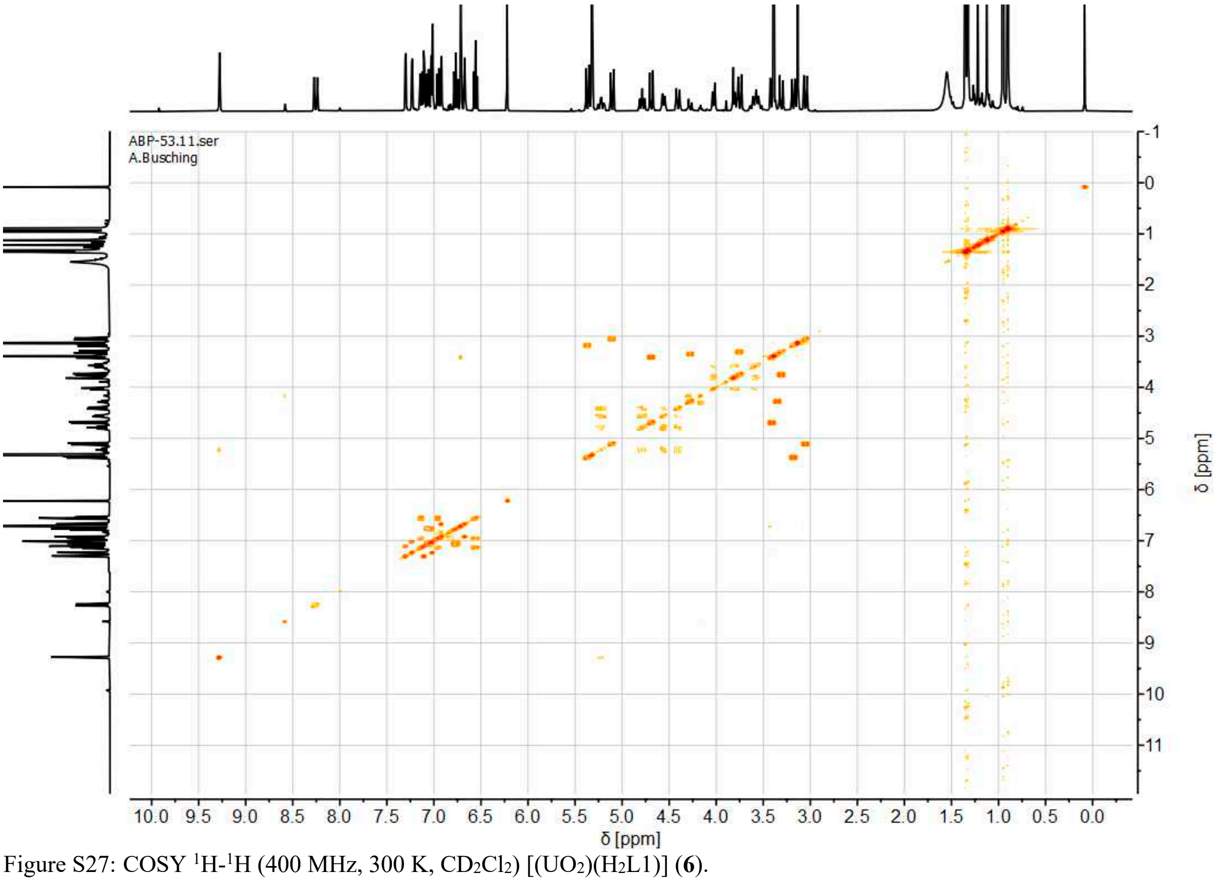

Figure S27: COSY ^{1}H-^{1}H (400 MHz, 300 K, CD_2Cl_2) [$(UO_2)(H_2L1)$] (**6**).

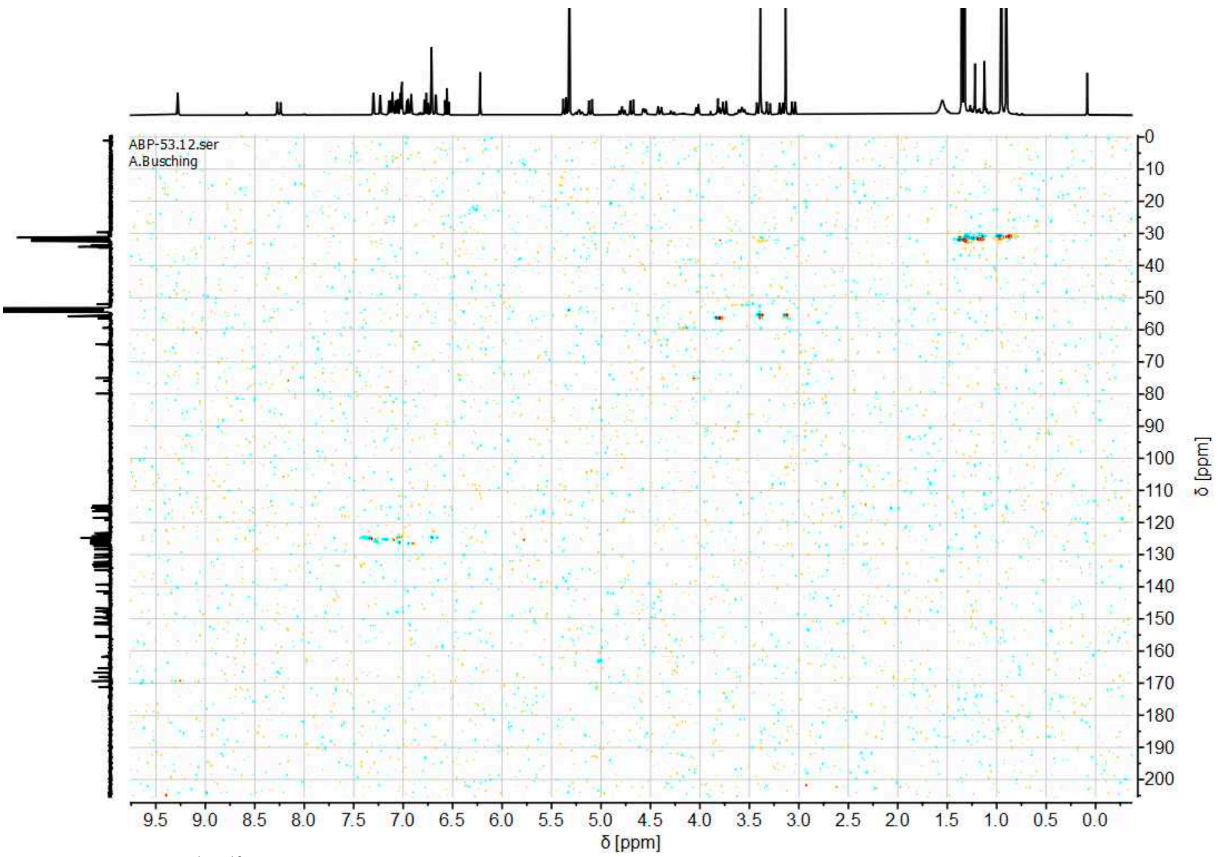

Figure S28: HSQC-^{1}H-^{13}C (400-100 MHz, 300 K, CD_2Cl_2) [$(UO_2)(H_2L1)$] (**6**).

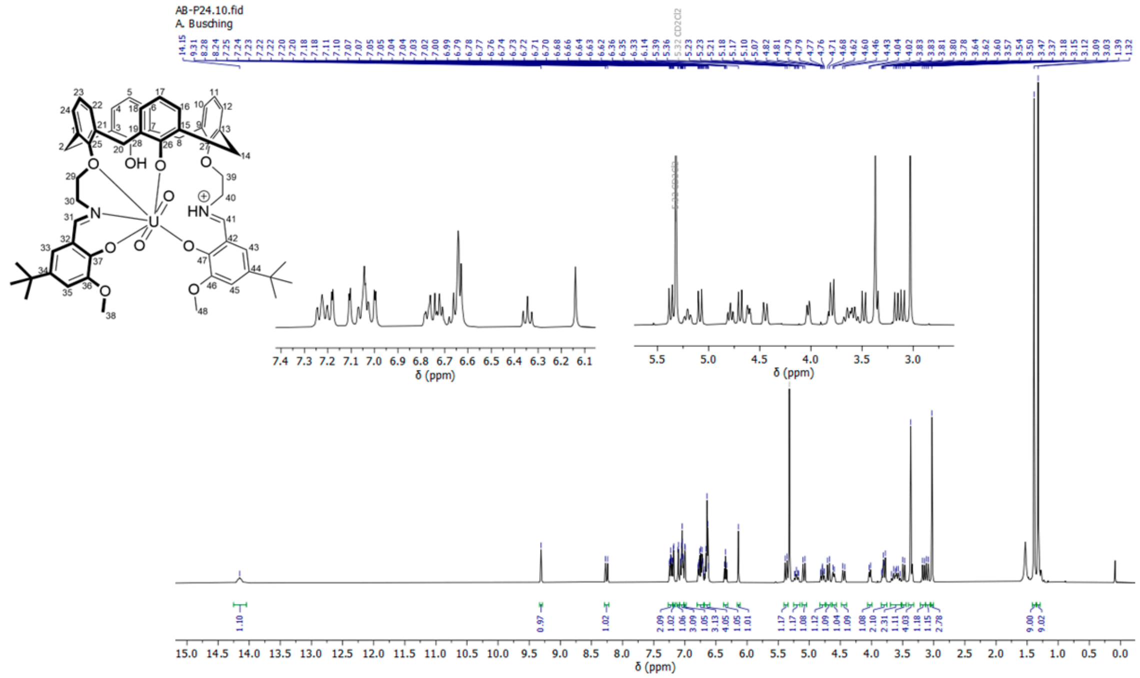

Figure S29: ^{1}H-NMR (400 MHz, 300 K, CD_2Cl_2) [$(UO_2)(H_2L2)$] (**7**).

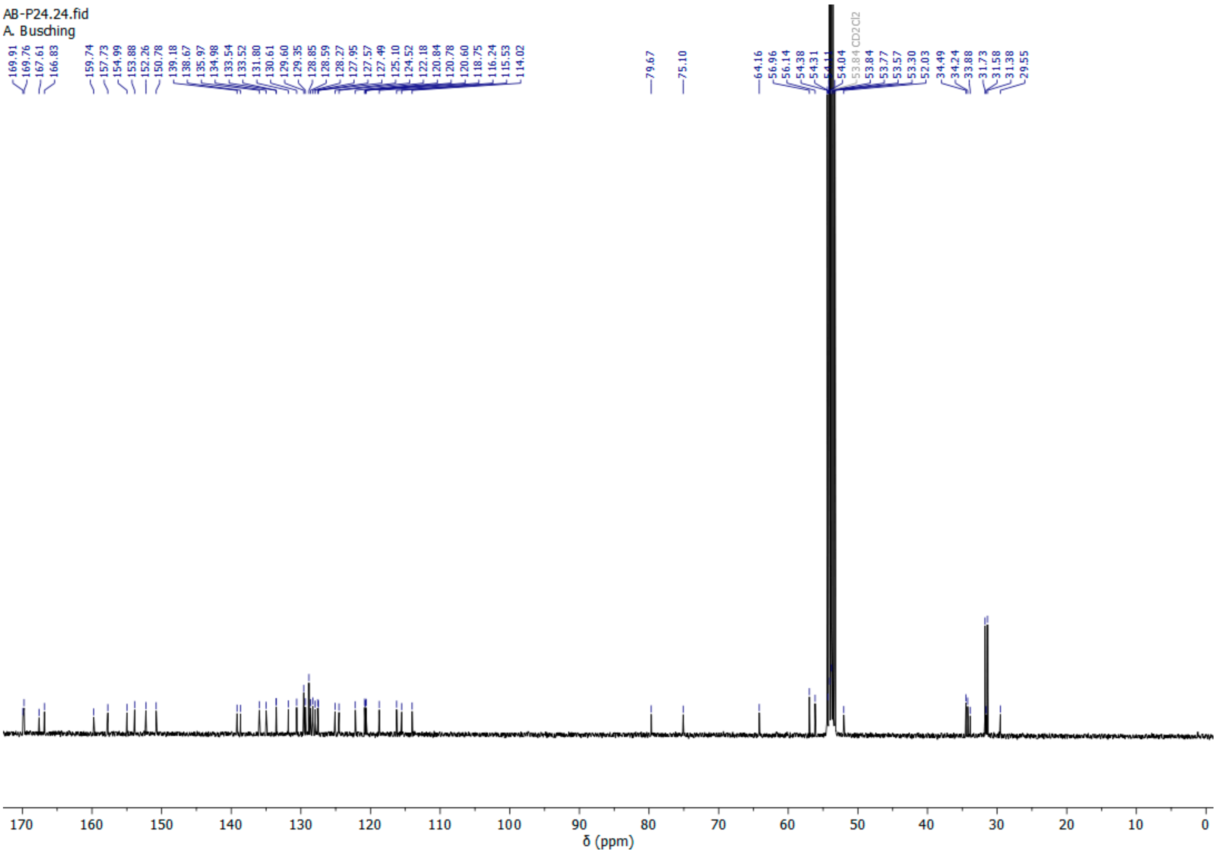

Figure S30: ^{13}C-NMR (100 MHz, 300 K, CD_2Cl_2) [$(UO_2)(H_2L2)$] (**7**).

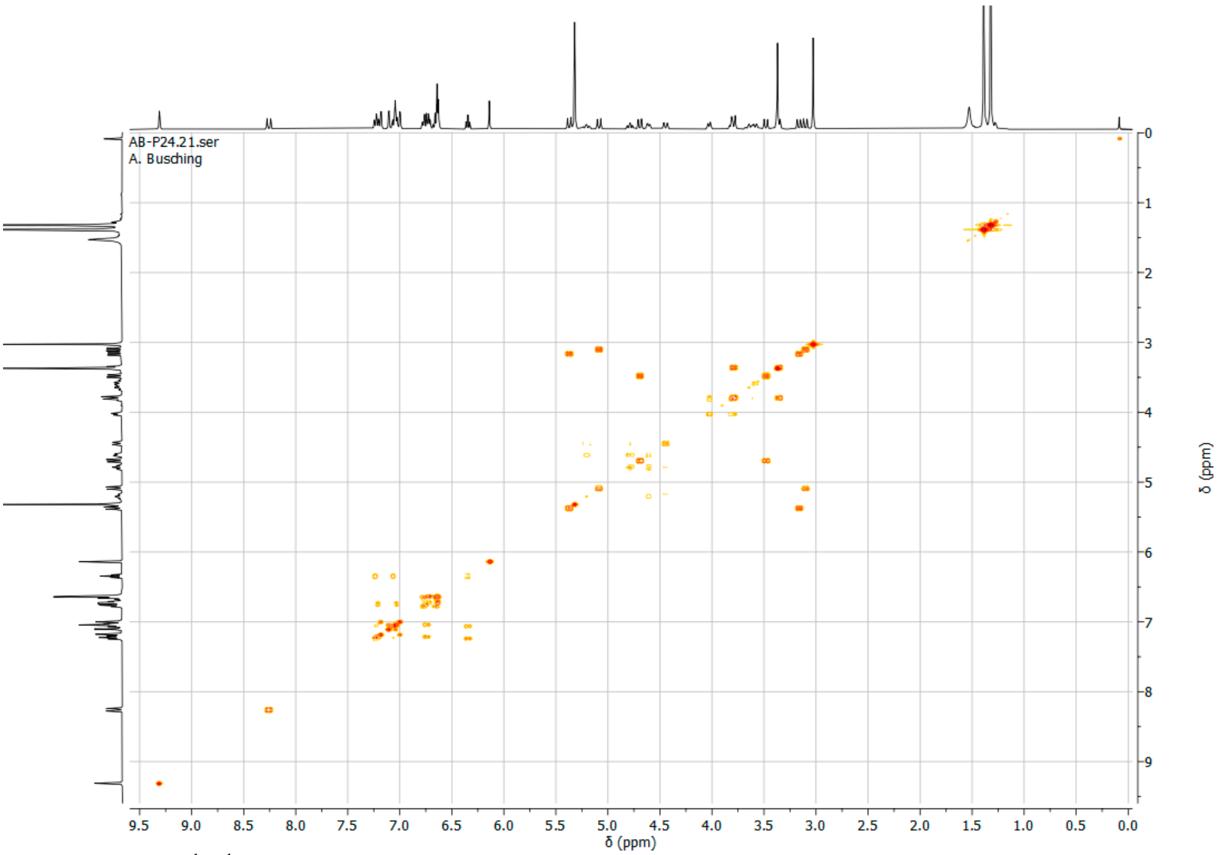

Figure S31: COSY ^{1}H-^{1}H (400 MHz, 300 K, CD_2Cl_2) [$(UO_2)(H_2L2)$] (**7**).

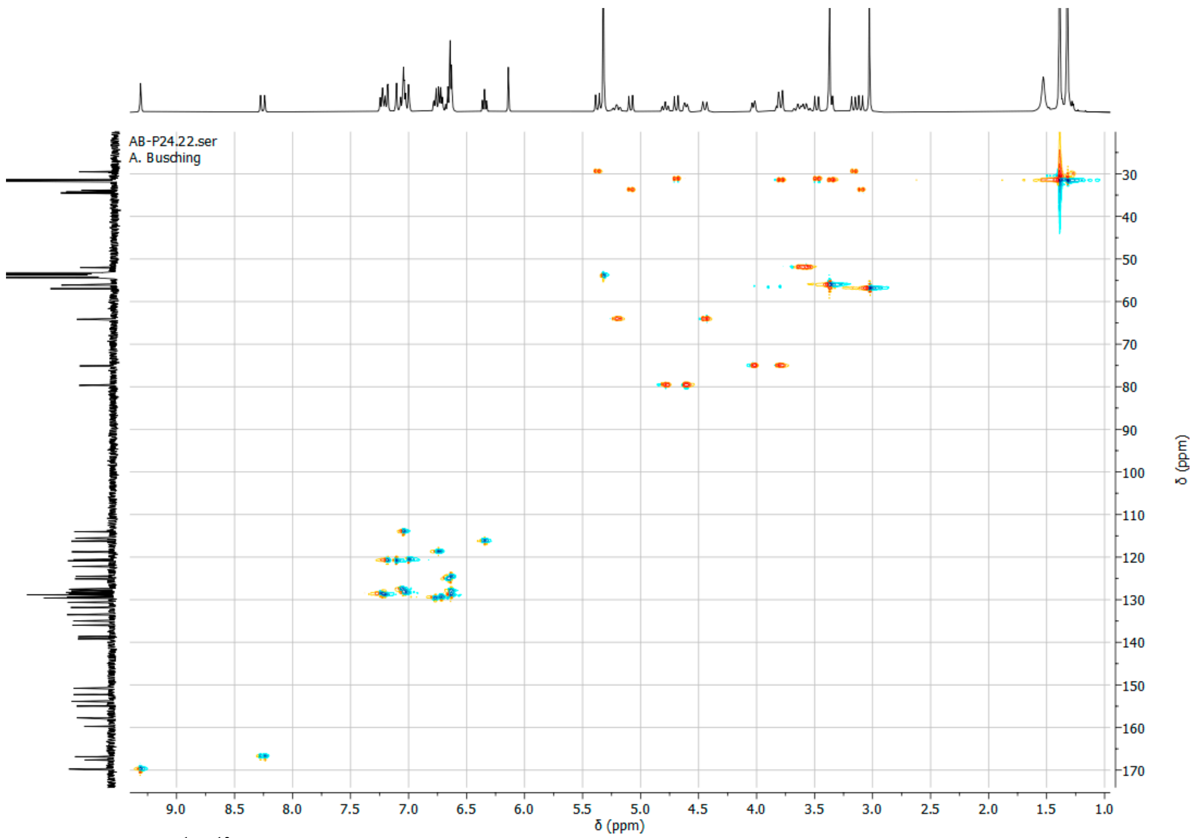

Figure S32: HSQC ^{1}H-^{13}C (400-100 MHz, 300 K, CD_2Cl_2) [$(UO_2)(H_2L2)$] (**7**).

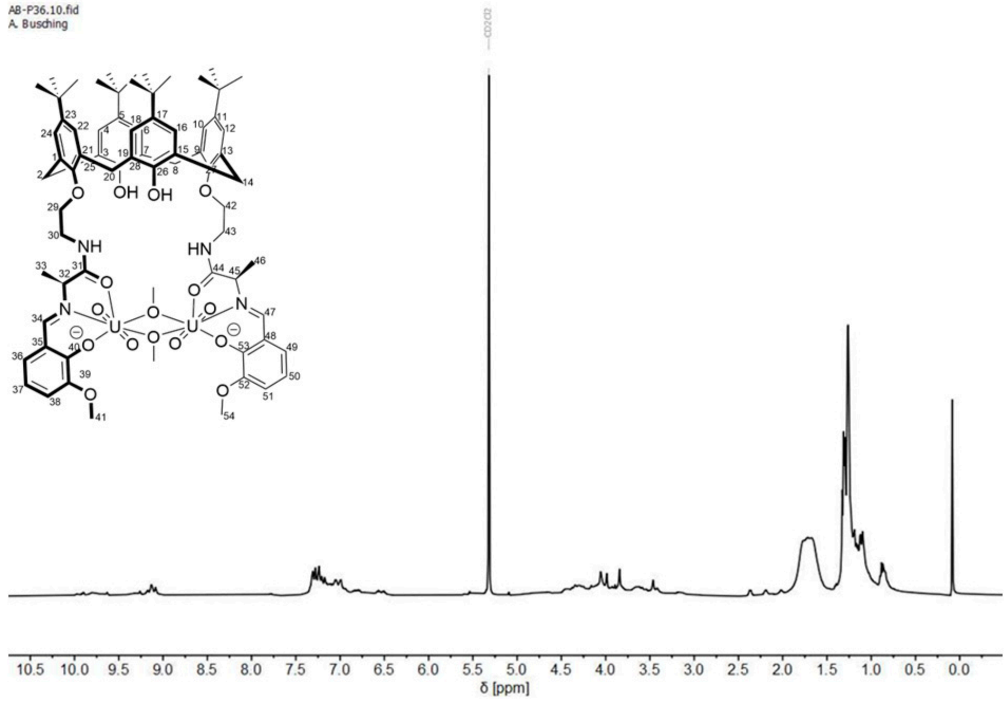

Figure S33: ^{1}H-NMR (400 MHz, 300 K, CD_2Cl_2) [$(UO_2)_2(H_2L3)(MeO)_2$] (**8**).

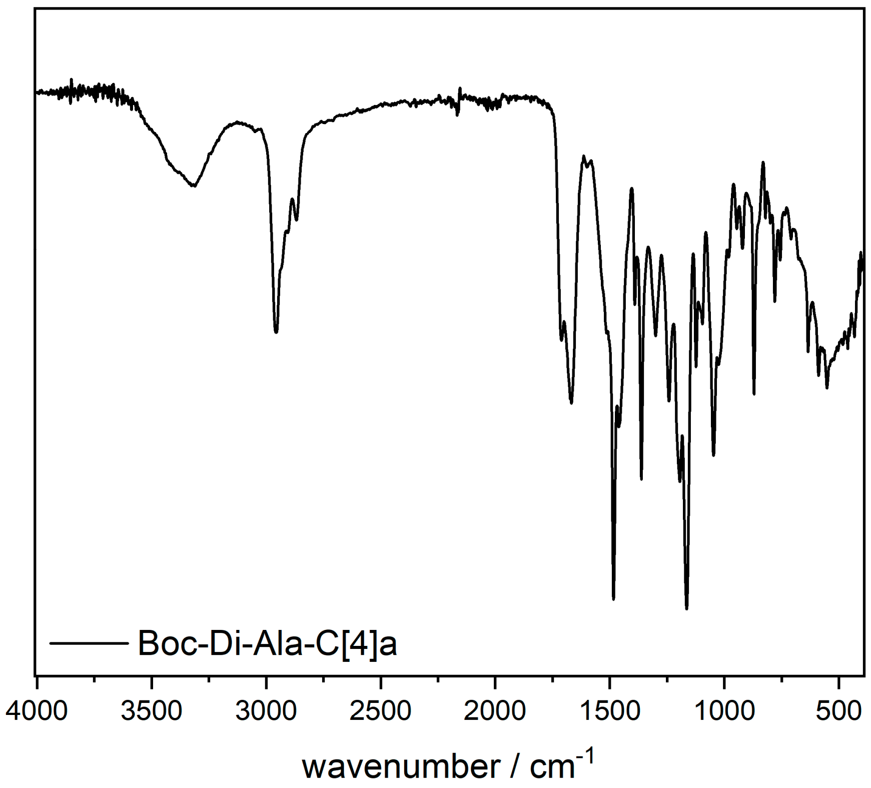

Figure S34: ATR-IR Boc-Di-Ala-Calix[4]arene.

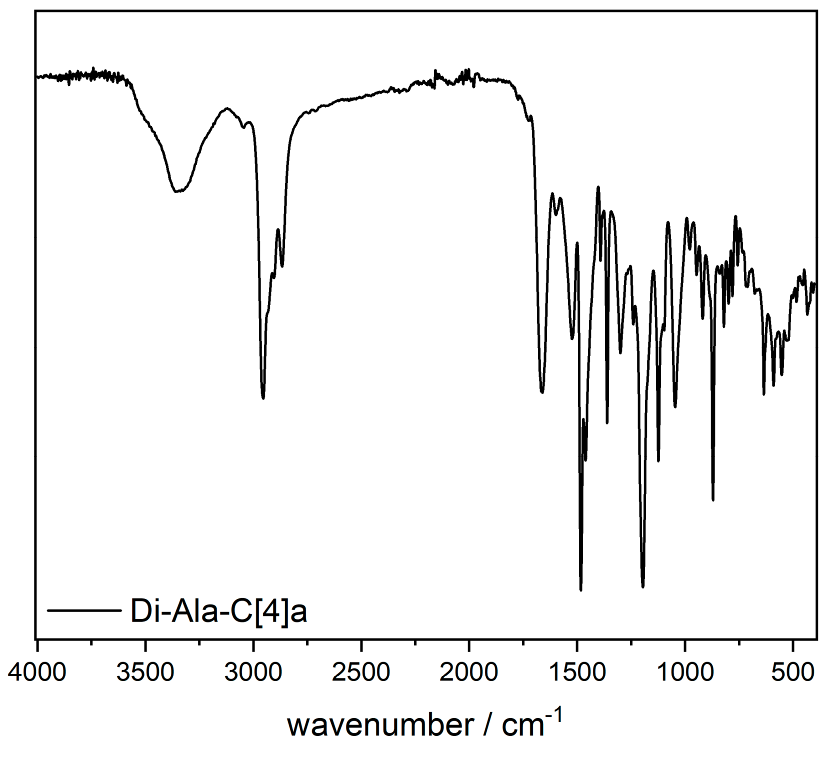

Figure S35: ATR-IR Di-Ala-Calix[4]arene (**5**).

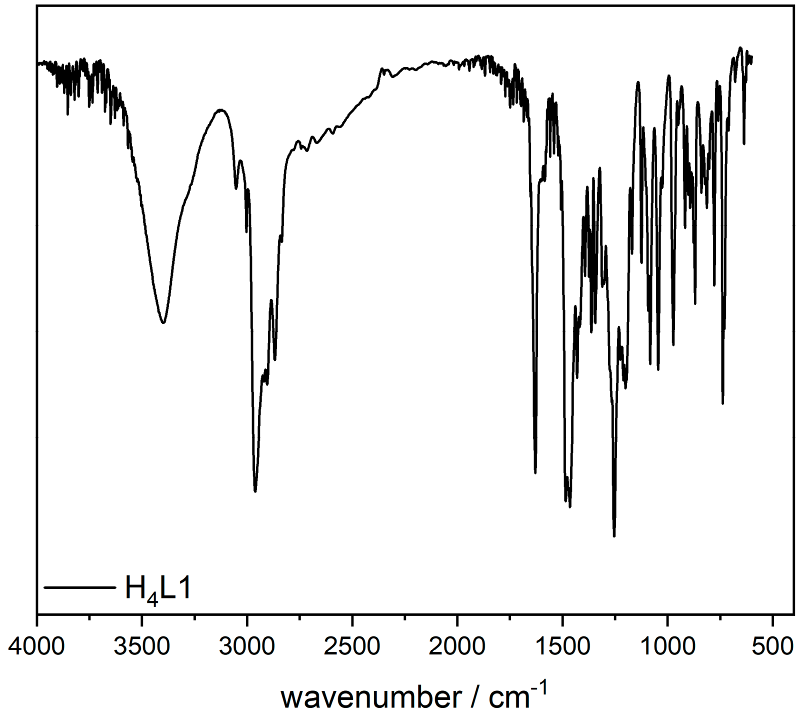

Figure S36: ATR-IR H_4L1.

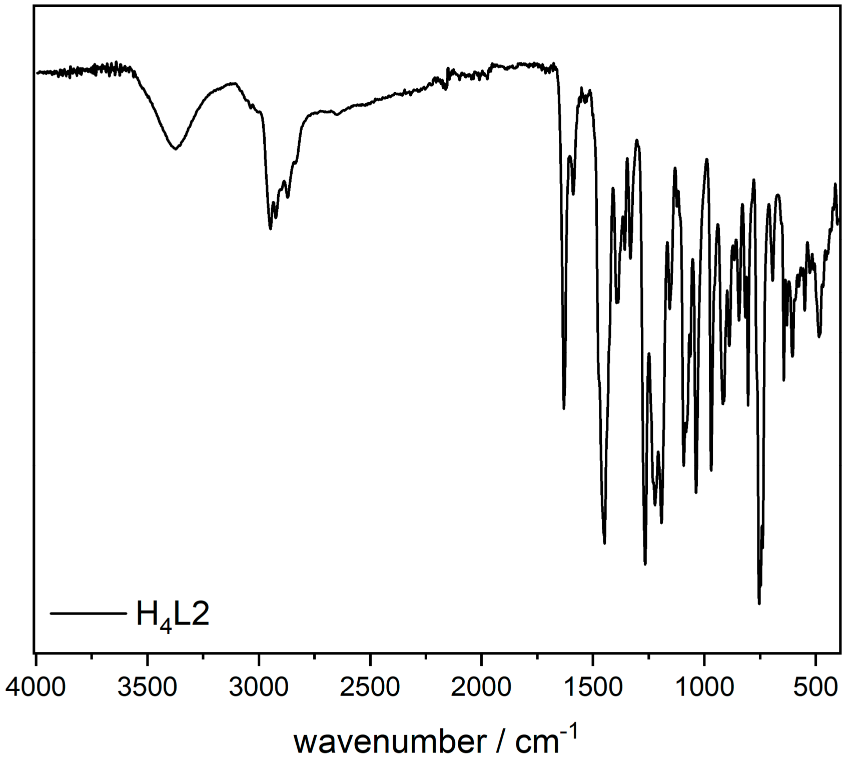

Figure S37: ATR-IR H_4L2.

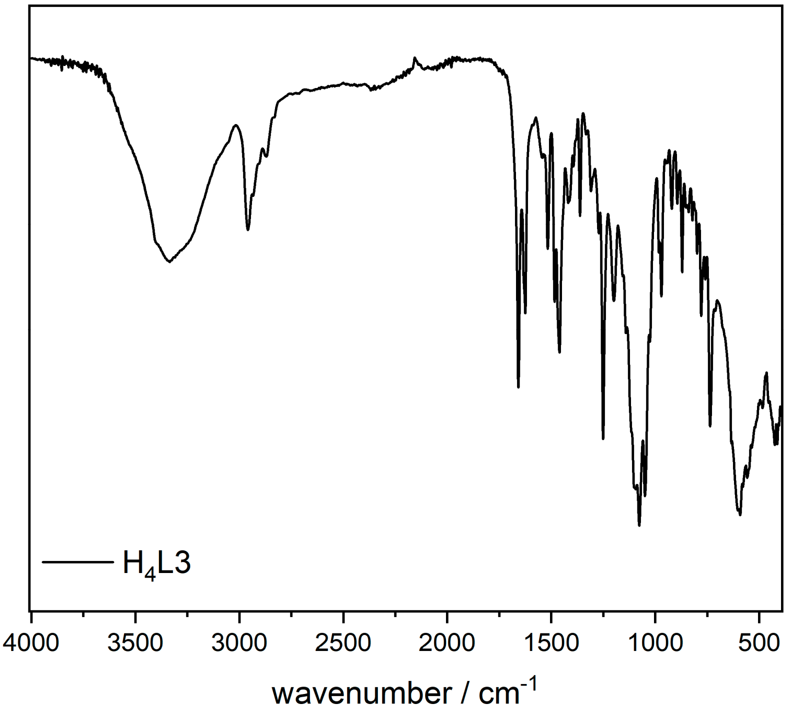

Figure S38: ATR-IR H_4L3.

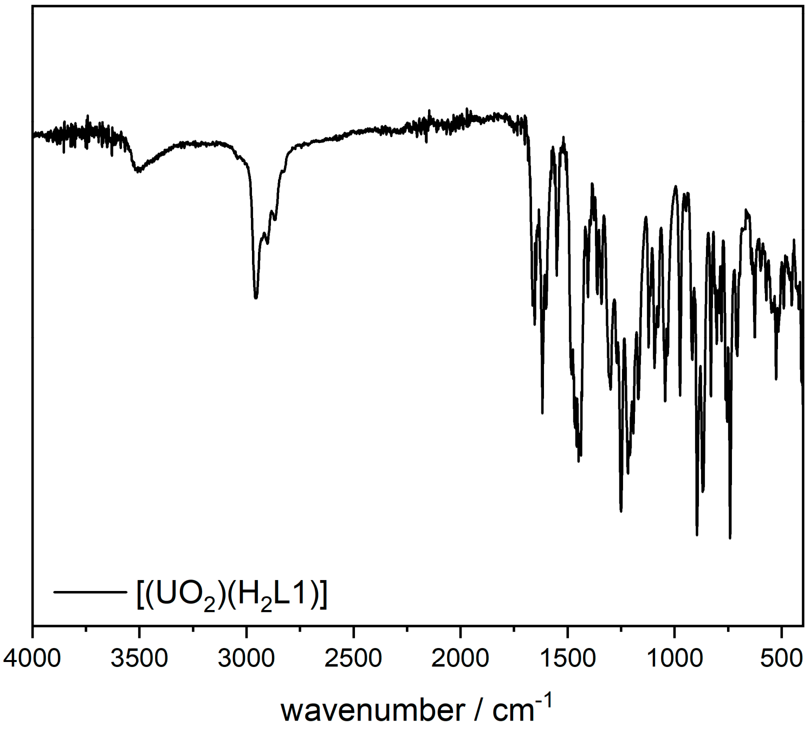

Figure S39: ATR-IR $[(UO_2)(H_2L1)]$ (**6**).

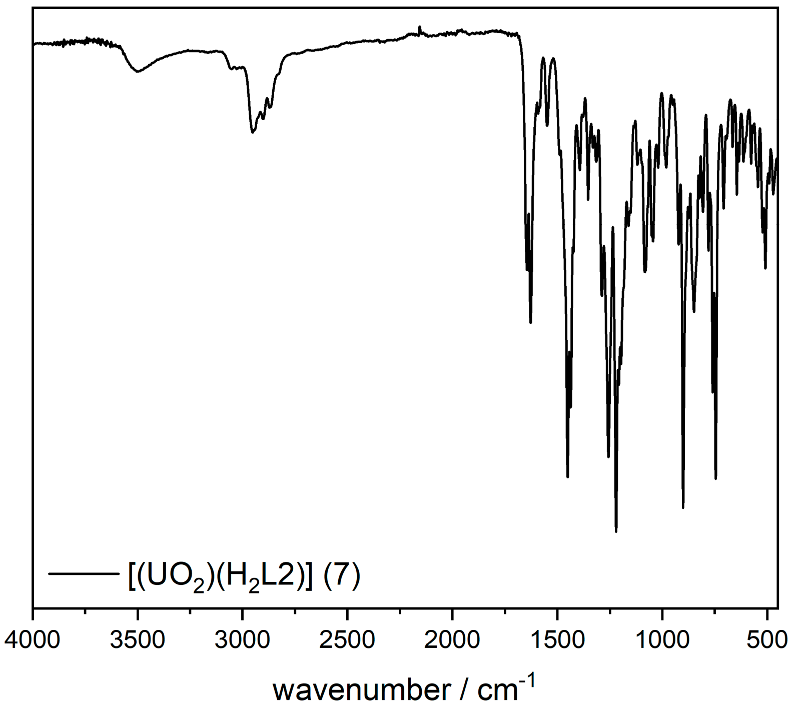

Figure S40: ATR-IR $[(UO_2)(H_2L2)]$ (**7**).

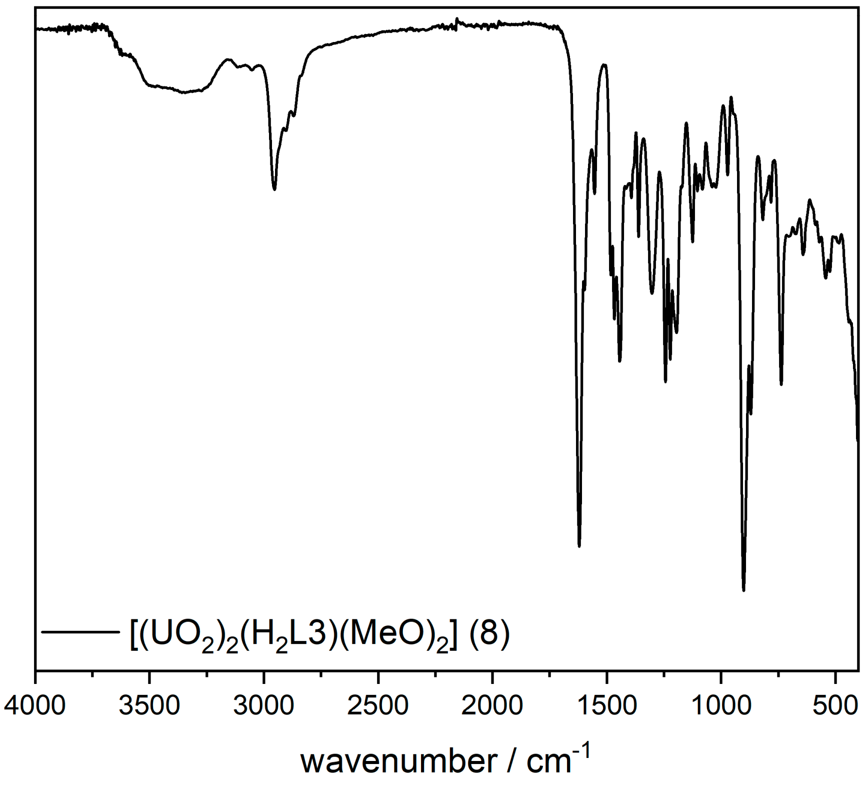

Figure S41: ATR-IR $[(UO_2)_2(H_2L3)(MeO)_2]$ (**8**).

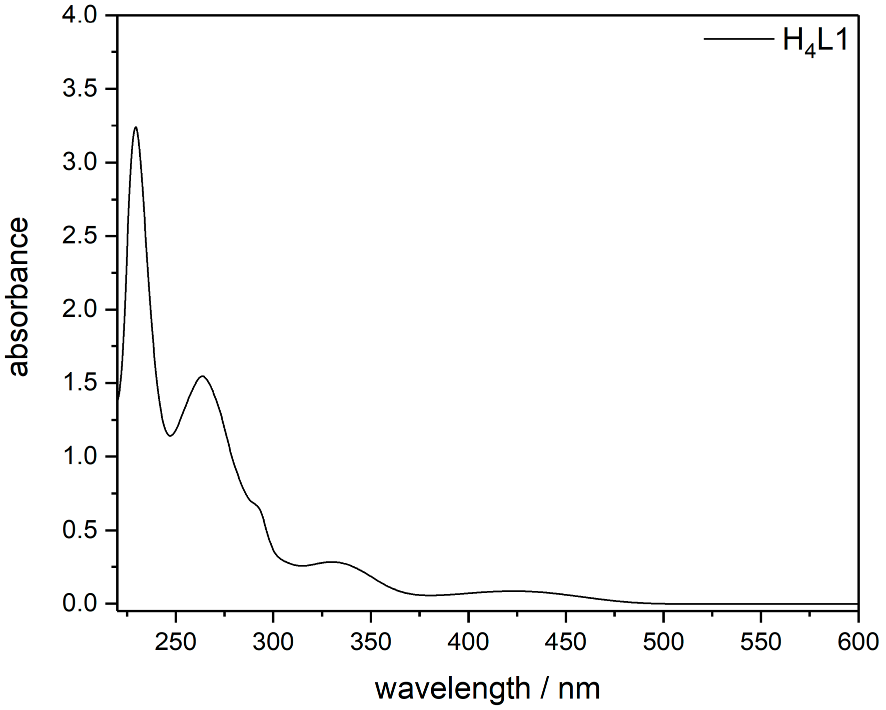

Figure S42: UV-vis spectrum of H_4L1 recorded in DCM/MeOH (c = 10^{-5} M).

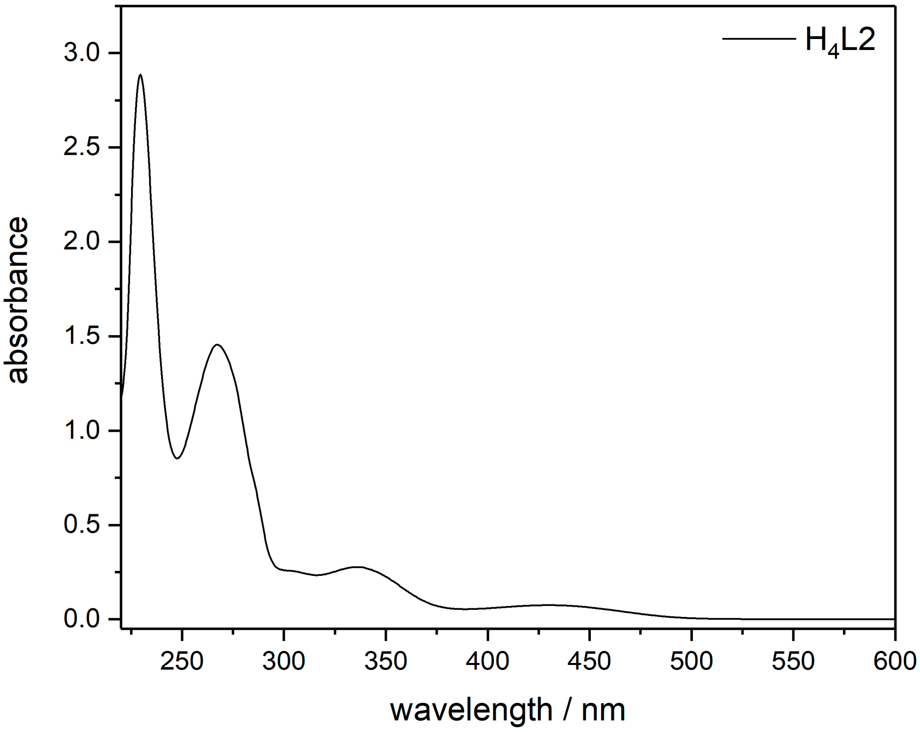

Figure S43: UV-vis spectrum of H_4L2 recorded in DCM/MeOH (c = 10^{-5} M).

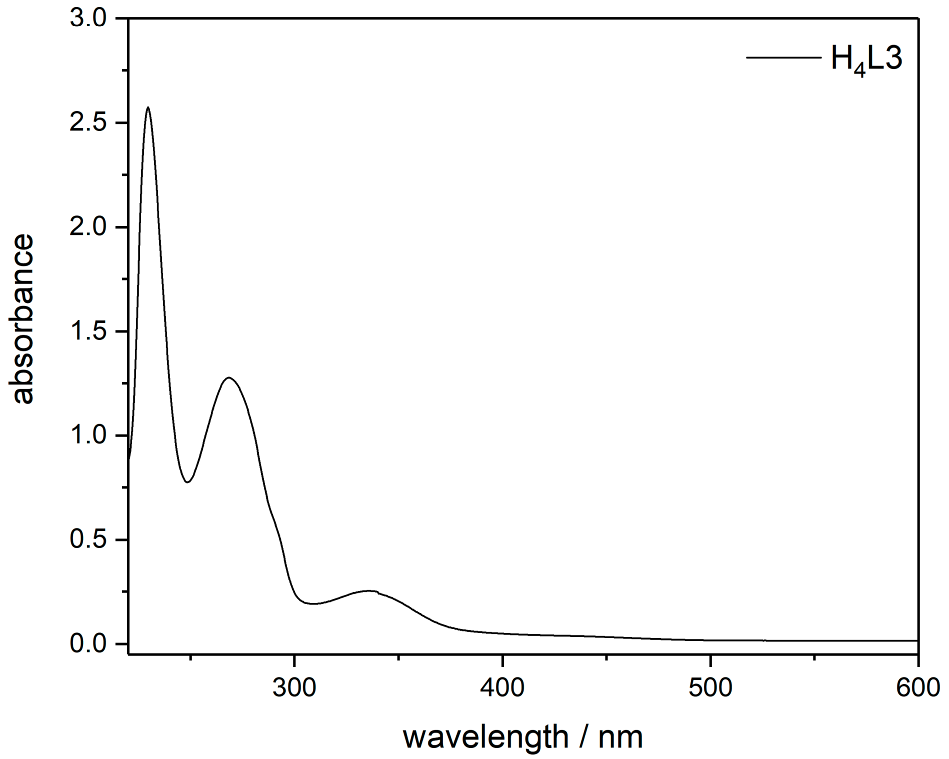

Figure S44: UV-vis spectrum of H_4L3 recorded in DCM/MeOH (c = 10^{-5} M).

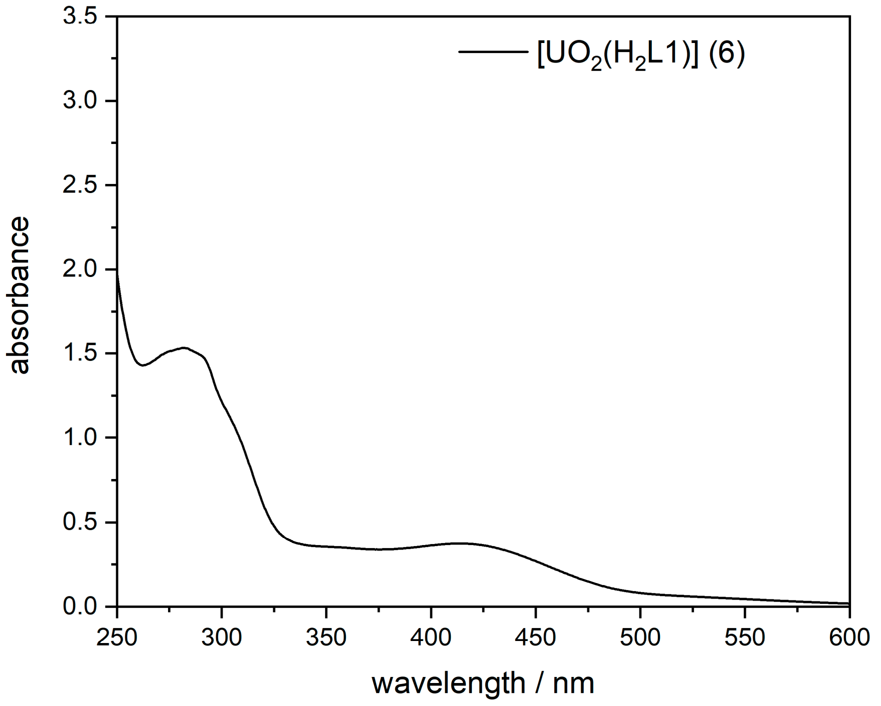

Figure S45: UV-vis spectrum of $[(UO_2)(H_2L1)]$ (**6**). recorded in DCM/MeOH (c = 10^{-5} M).

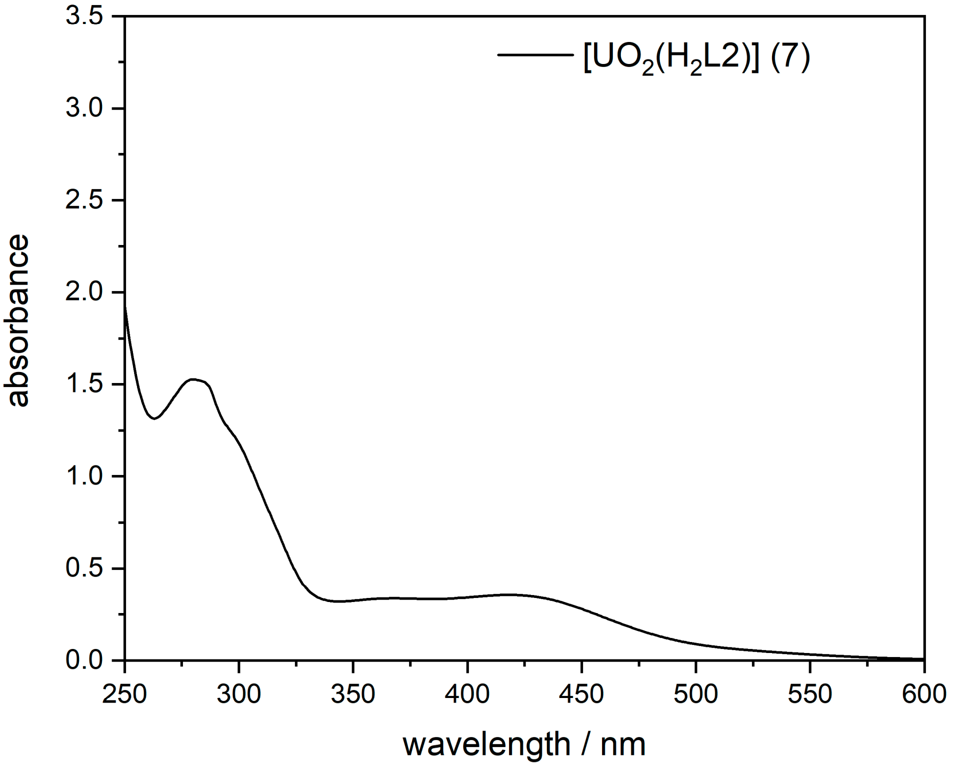

Figure S46: UV-vis spectrum of [$(UO_2)(H_2L2)$] (**7**). recorded in DCM/MeOH (c = 10^{-5} M).

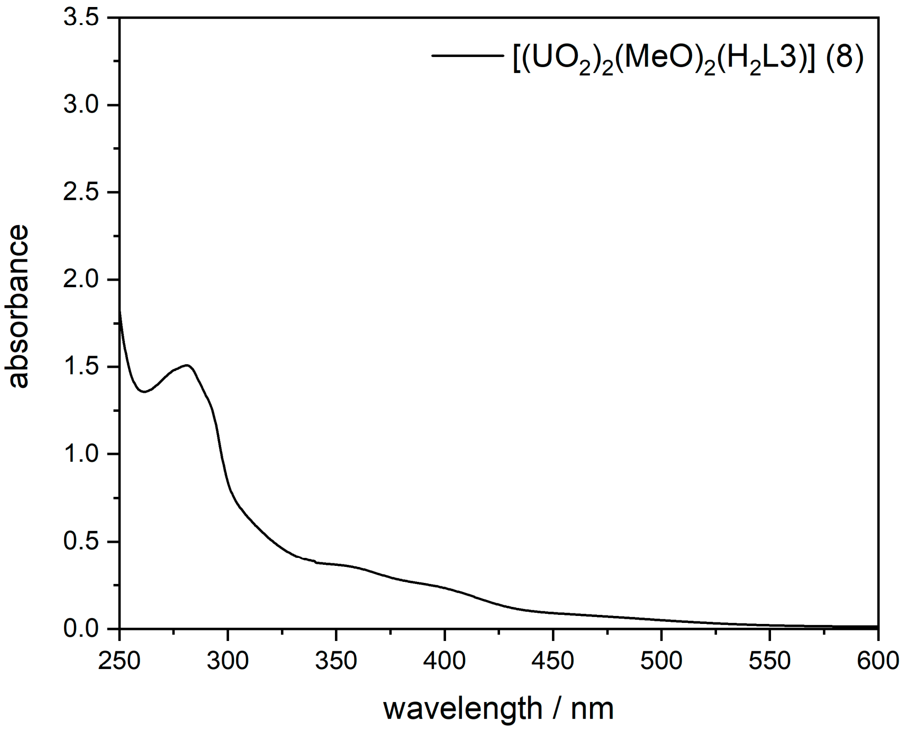

Figure S47: UV-vis spectrum of [$(UO_2)_2(H_2L3)(MeO)_2$] (**8**). recorded in DCM/MeOH (c = 10^{-5} M).

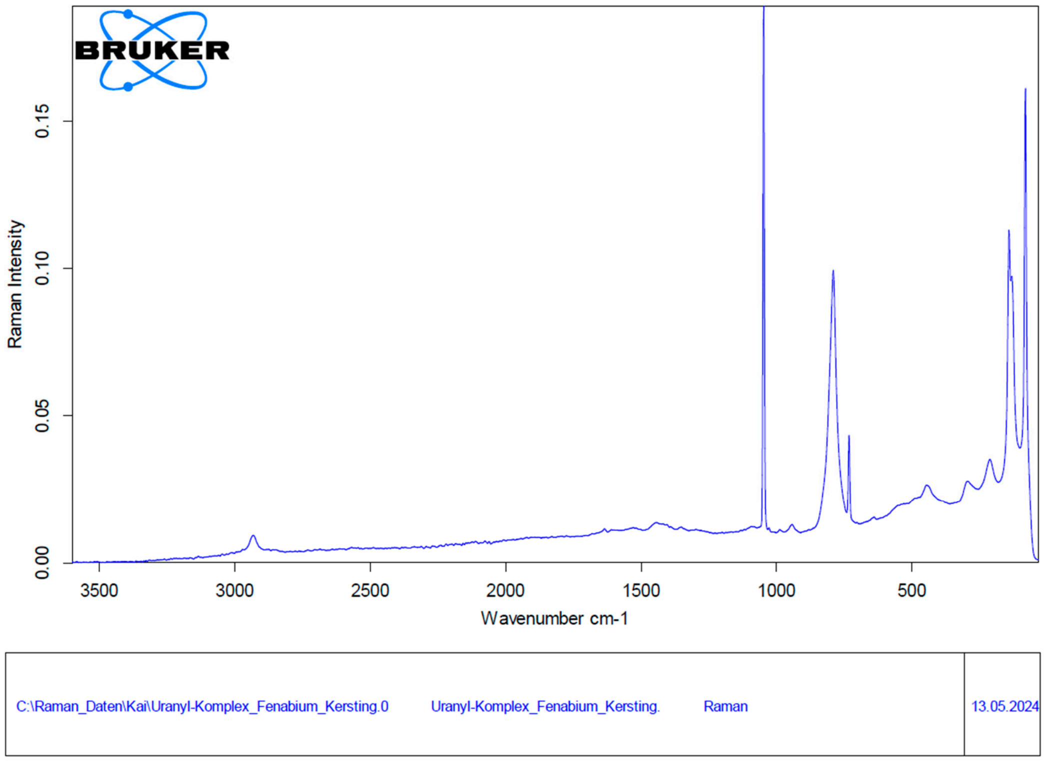

Figure S48: Raman spectrum of complex [$UO_2(H_2L2)$] (**7**).

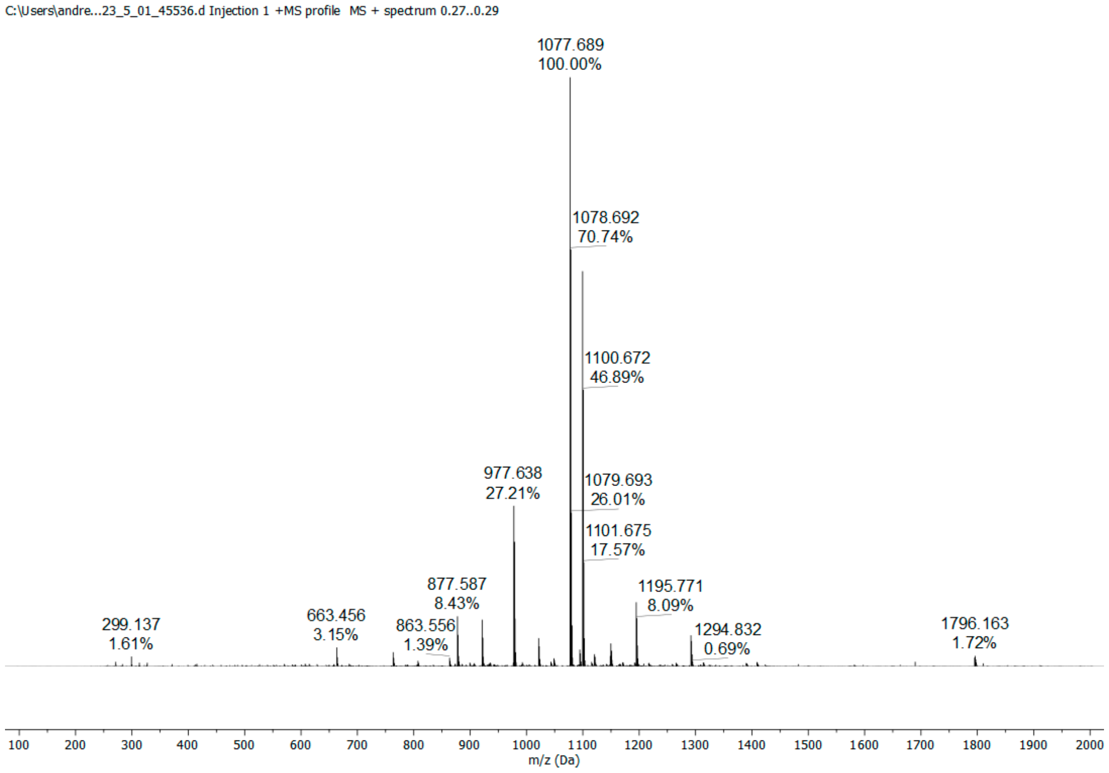

Figure S49: ESI(+)-MS of Boc-Di-Ala-Calix[4]arene.

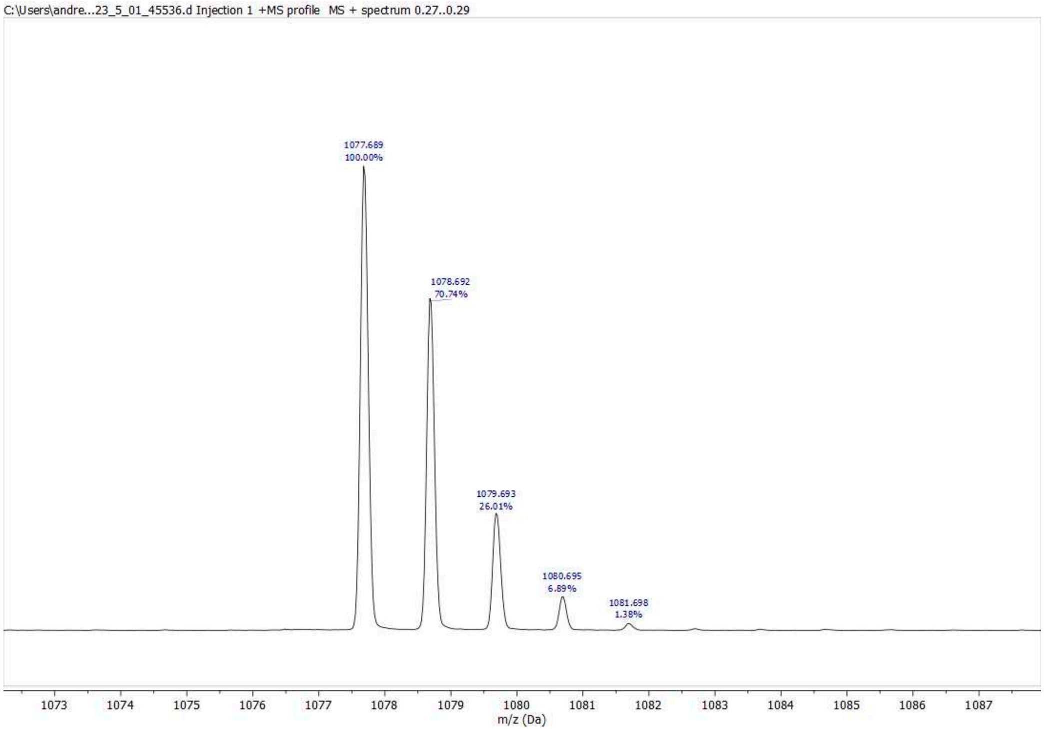

Figure S50: Expended region of the ESI(+)-MS of Boc-Di-Ala-Calix[4]arene.

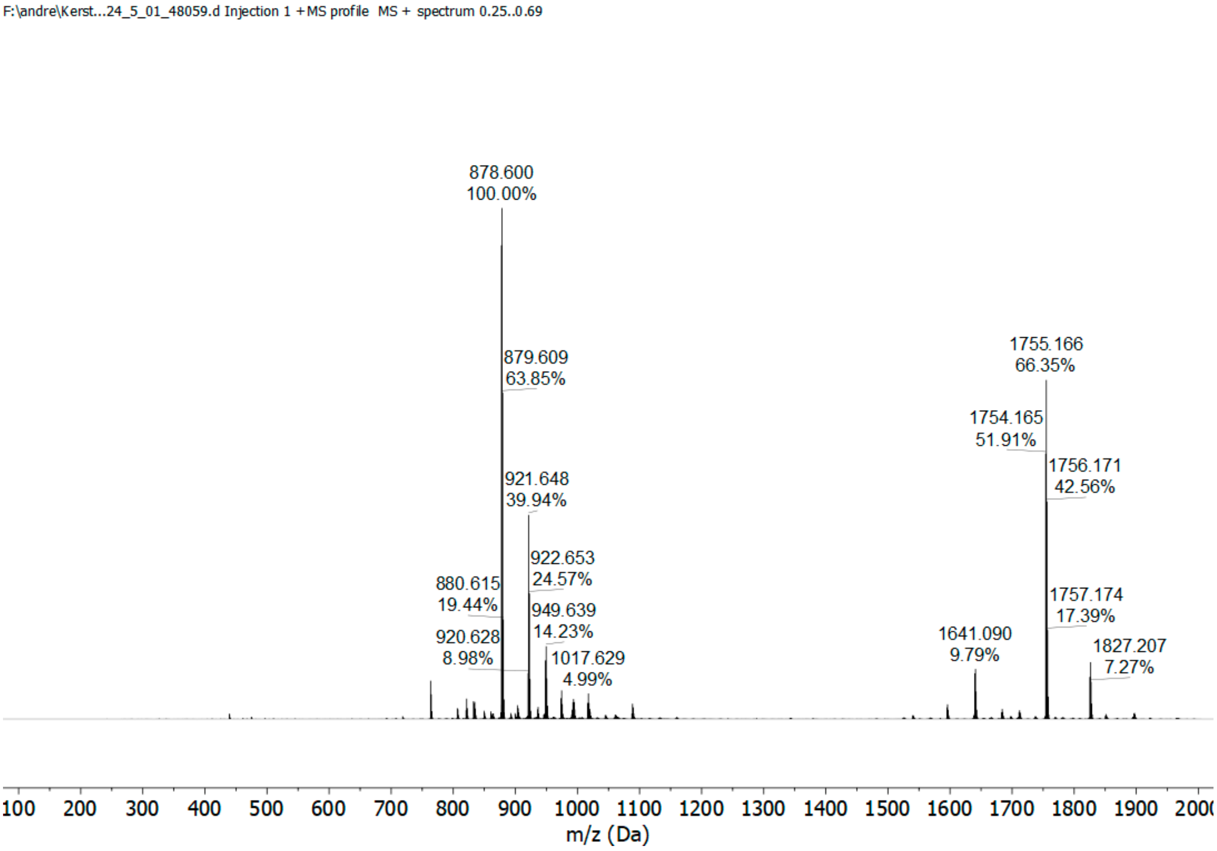

Figure S50: Expended region of the ESI(+)-MS of Di-Ala-Calix[4]arene (**5**).

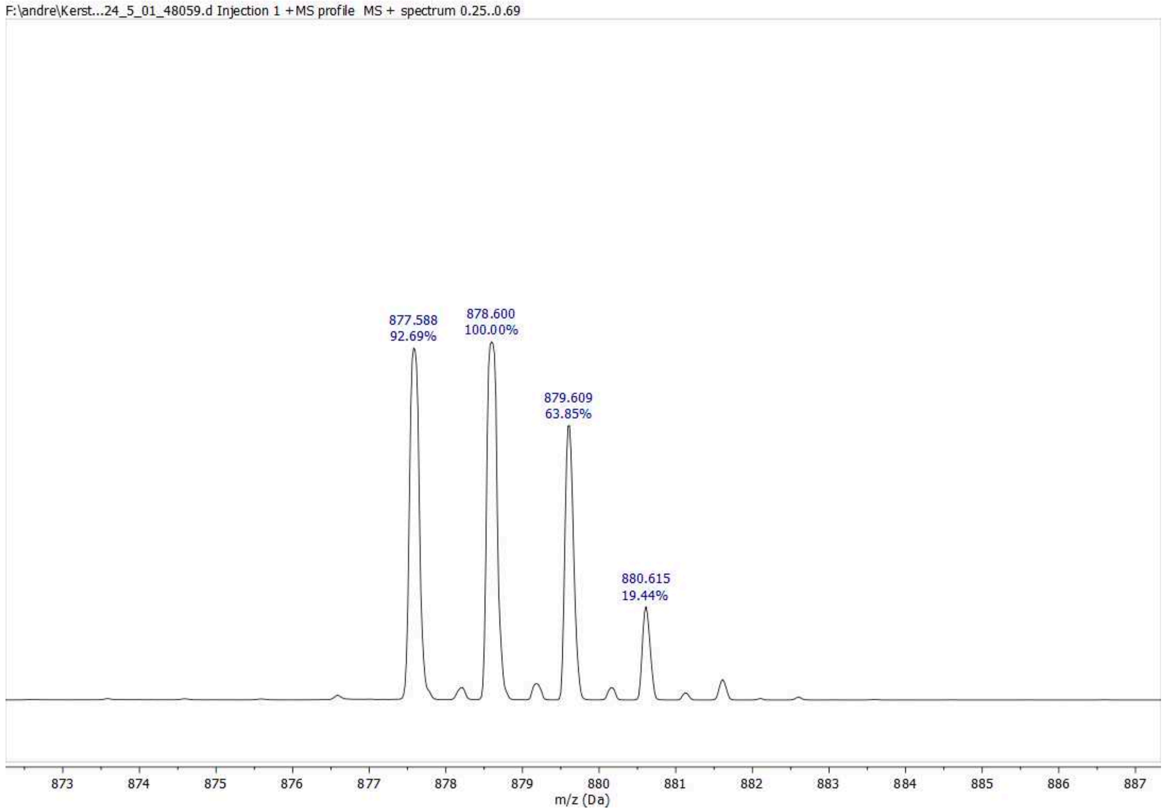

Figure S51: Expended region of the ESI(+)-MS of Di-Ala-Calix[4]arene (**5**).

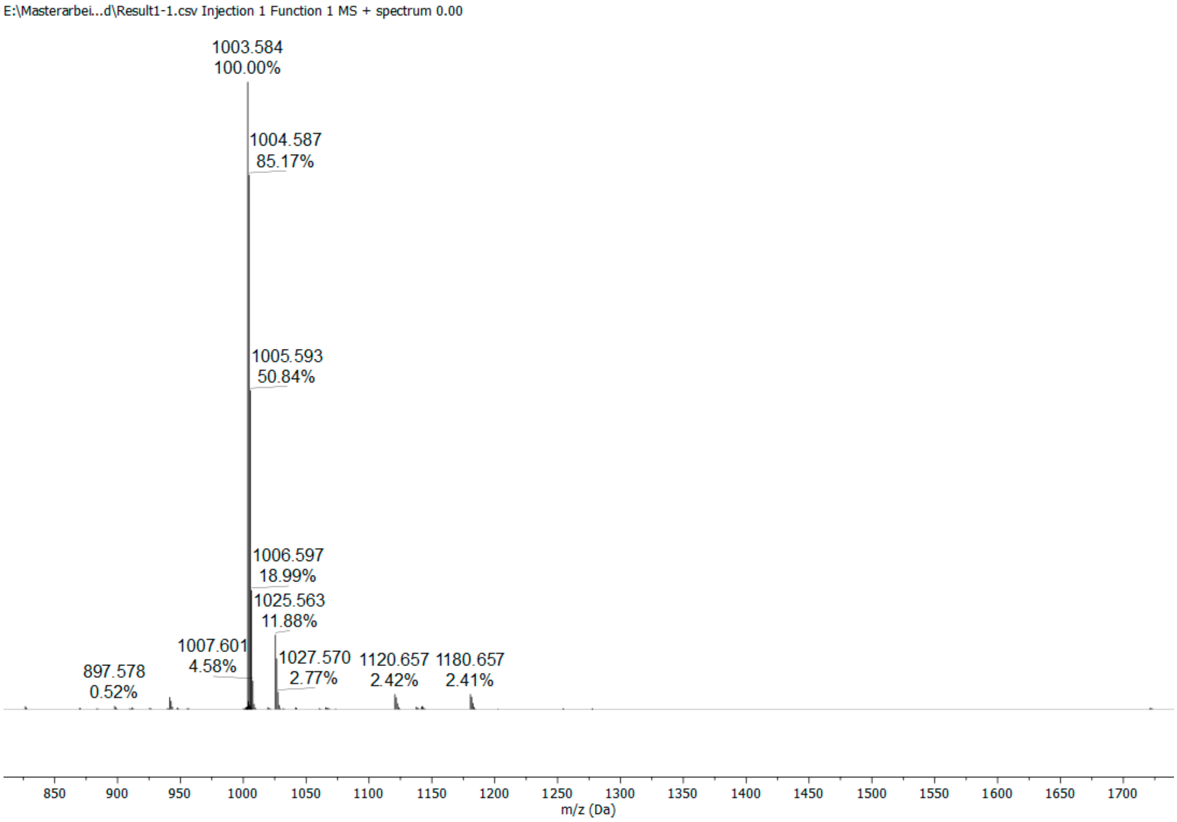

Figure S52: ESI(+)-MS of H_4L1.

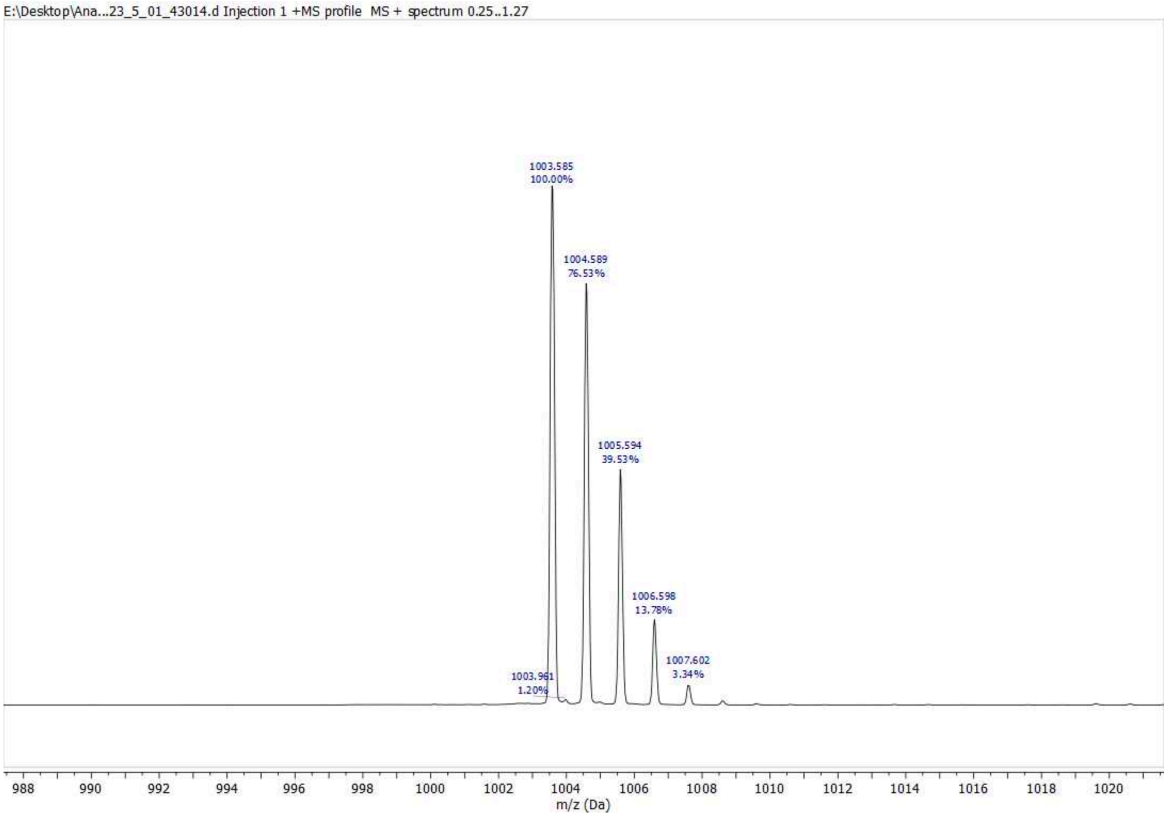

Figure S53: Expended region of the ESI(+)-MS of H_4L1.

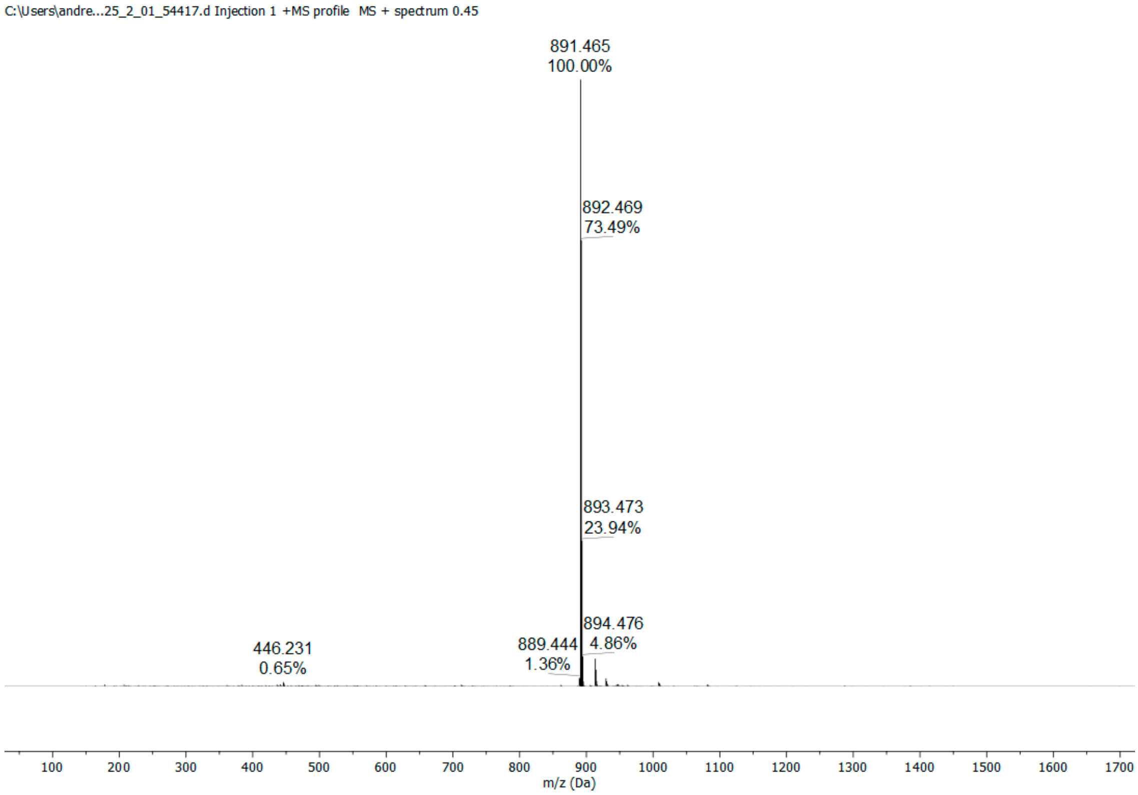

Figure S54: ESI(+)-MS of H_4L2.

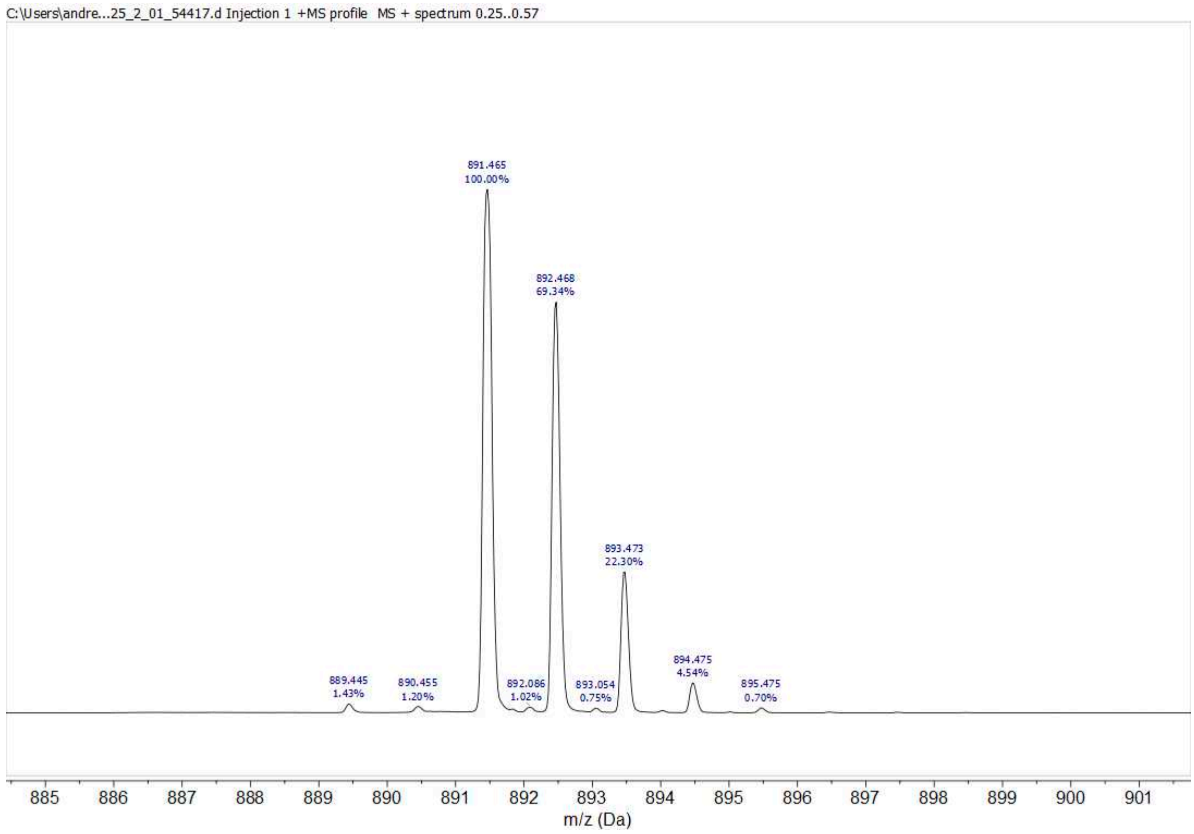

Figure S56 Expended region of the ESI(+)-MS of H_4L2.

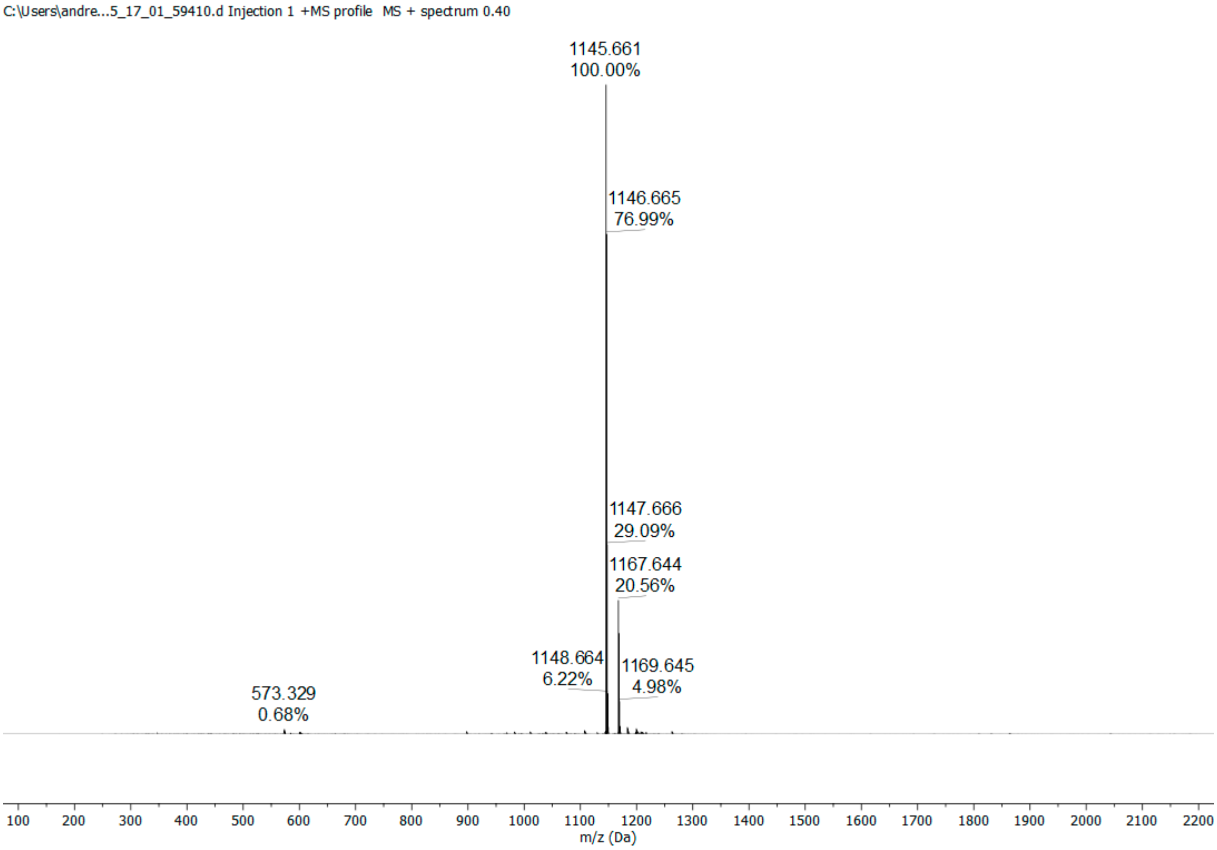

Figure S55: ESI(+)-MS of H_4L3.

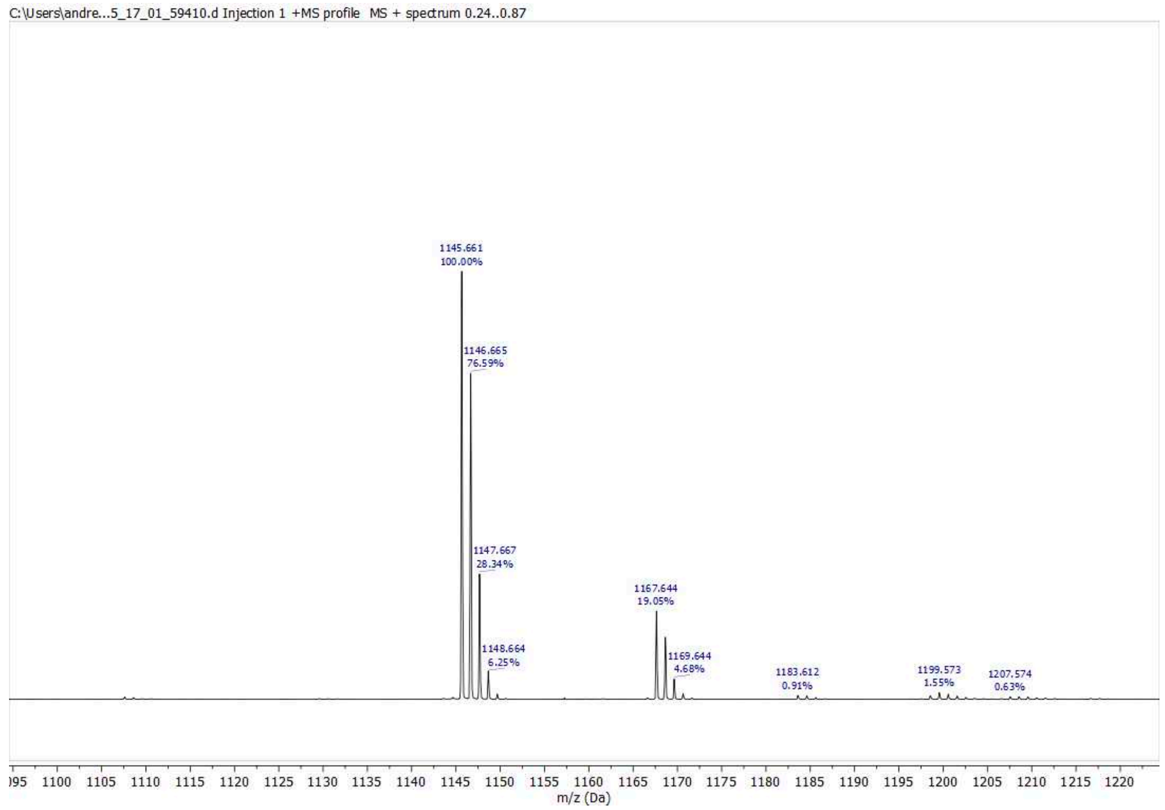

Figure S56: Expended region of the ESI(+)-MS of H_4L3.

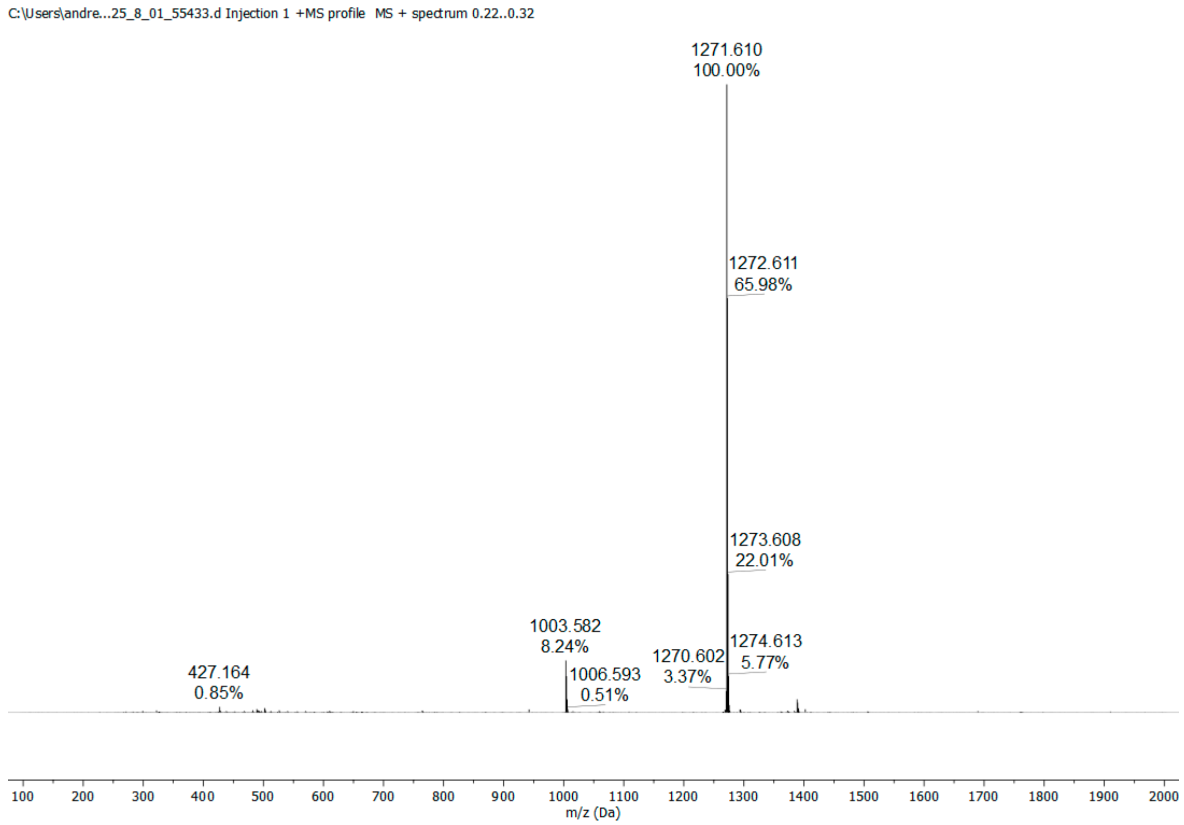

Figure S57: ESI(+)-MS of $[(UO_2)(H_2L1)]$ (**6**).

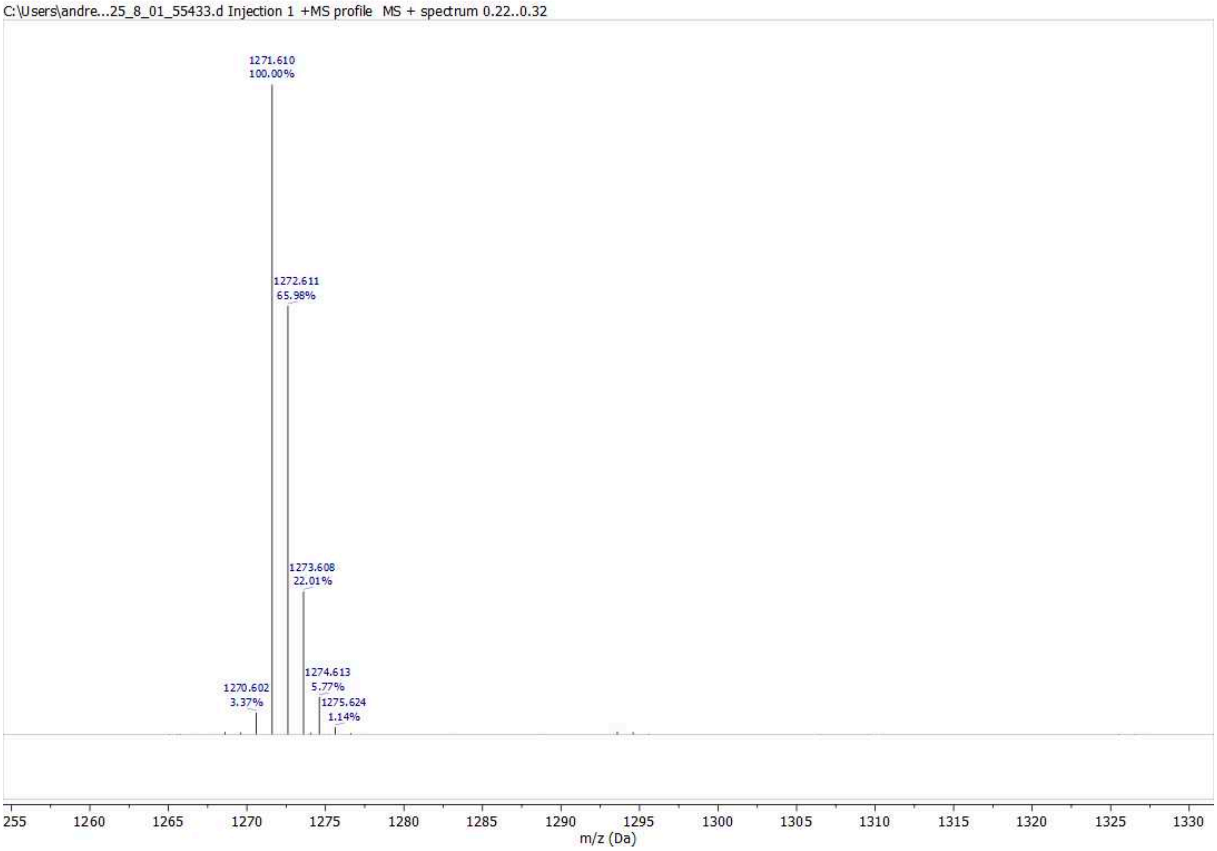

Figure S60: Expended region of the ESI(+)-MS of [$(UO_2)(H_2L1)$] (**6**).

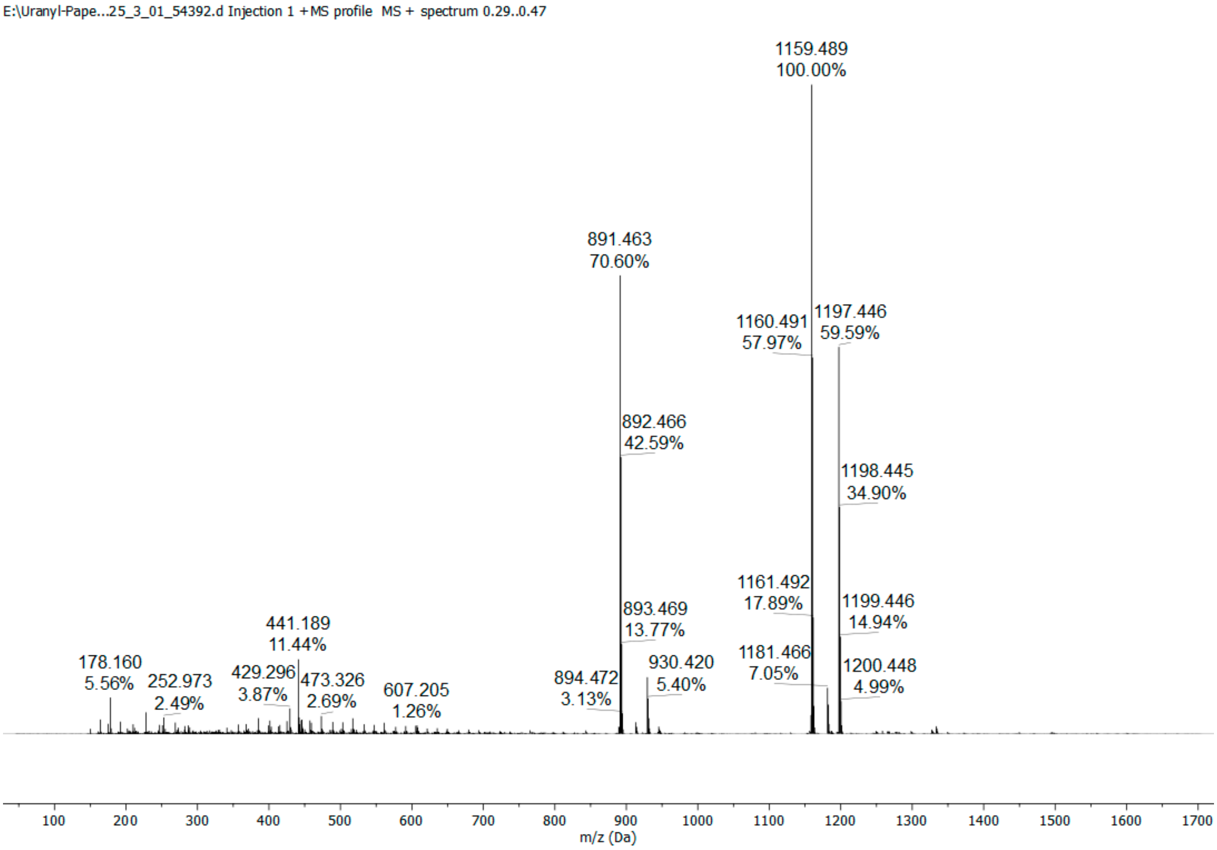

Figure S61: ESI(+)-MS of [$(UO_2)(H_2L2)$] (**7**).

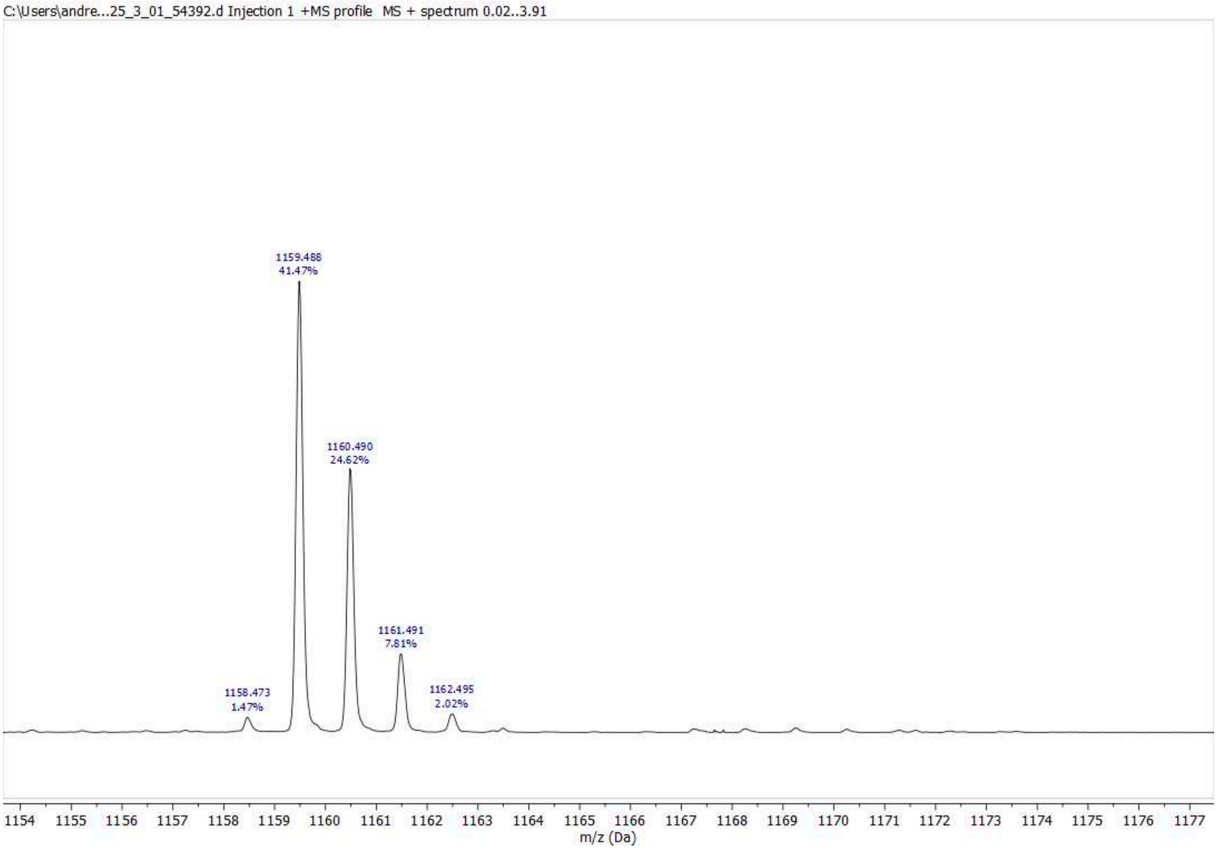

Figure S62: Expended region of the ESI(+)-MS of [(UO_2)(H_2L2)] (**7**).

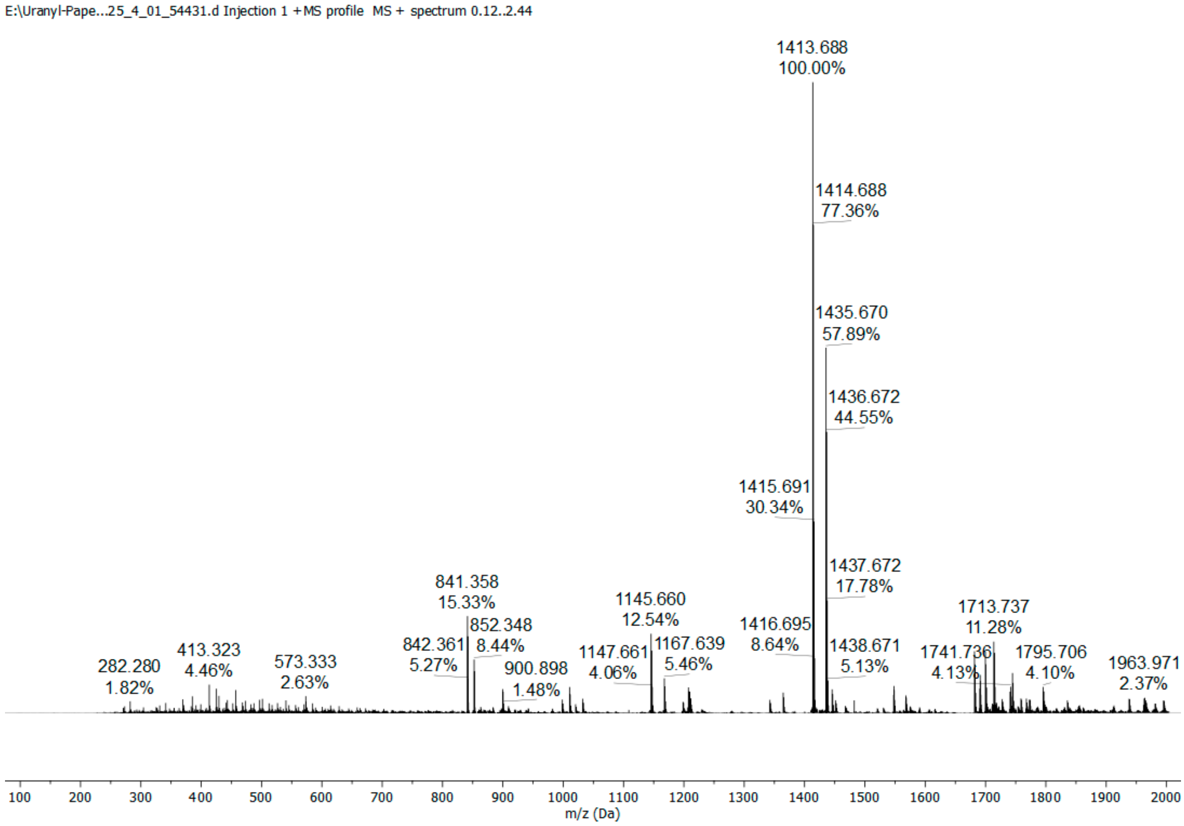

Figure S63: ESI(+)-MS of [$(UO_2)_2$(H_2L3)$(MeO)_2$] (**8**).

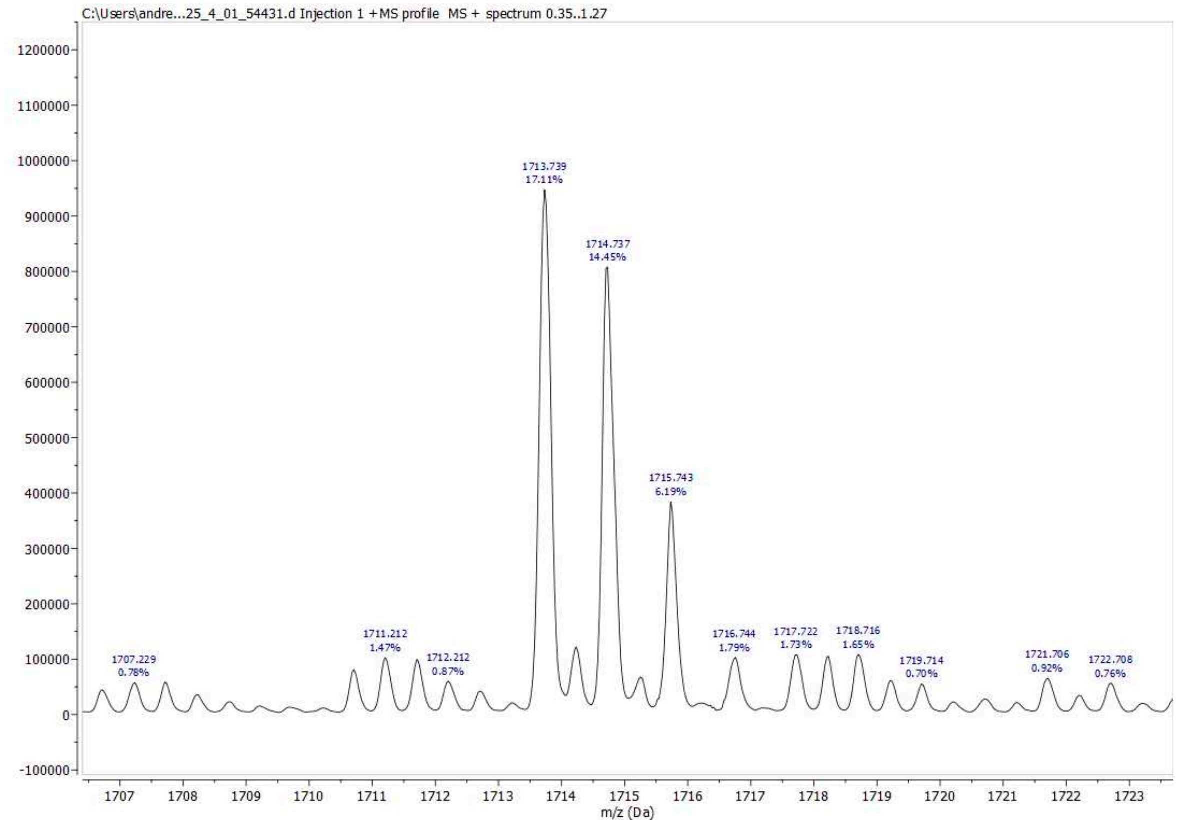

Figure S64: Excerpt of the ESI(+)-MS of $[(UO_2)_2(H_2L3)(MeO)_2]$ (**8**).

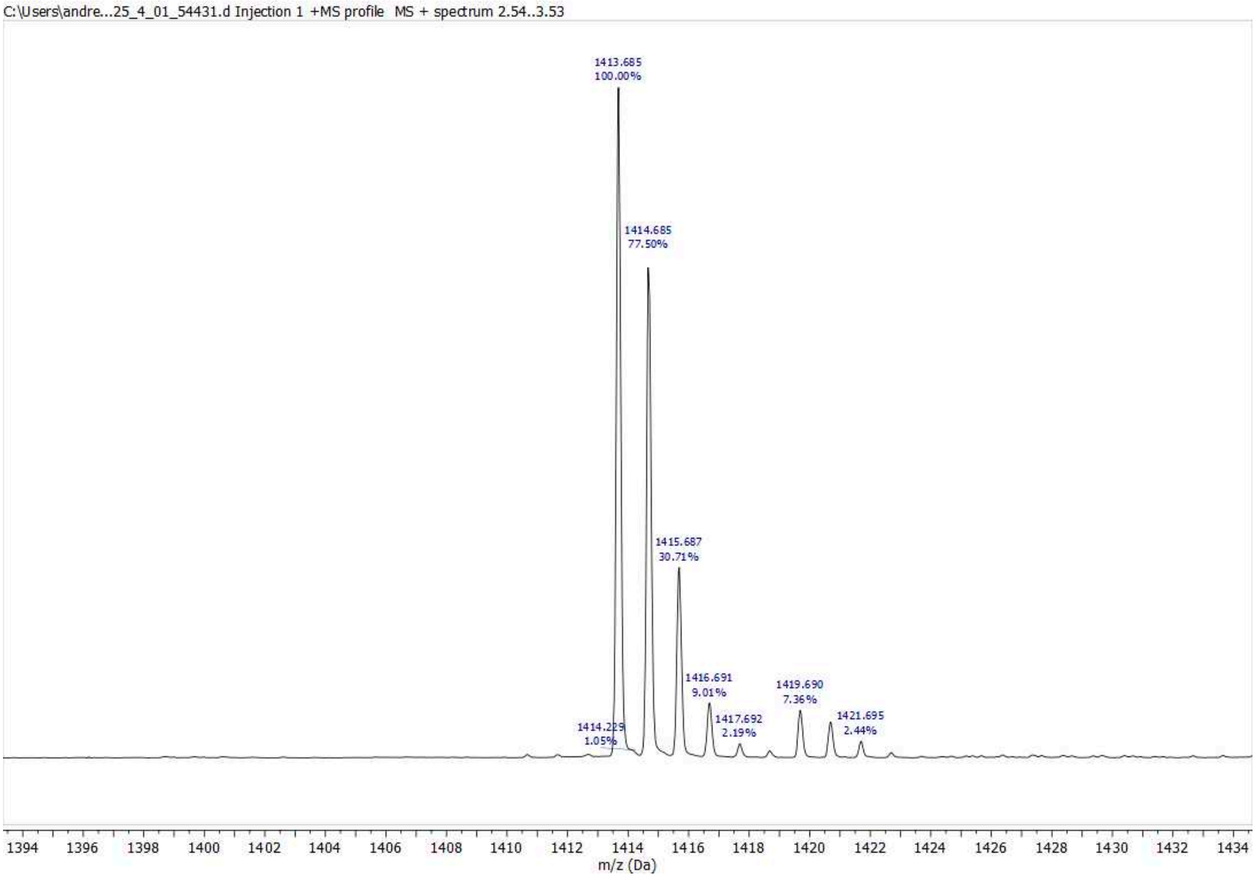

Figure S65: Excerpt of the ESI(+)-MS of $[(UO_2)_2(H_2L3)(MeO)_2]$ (**8**).

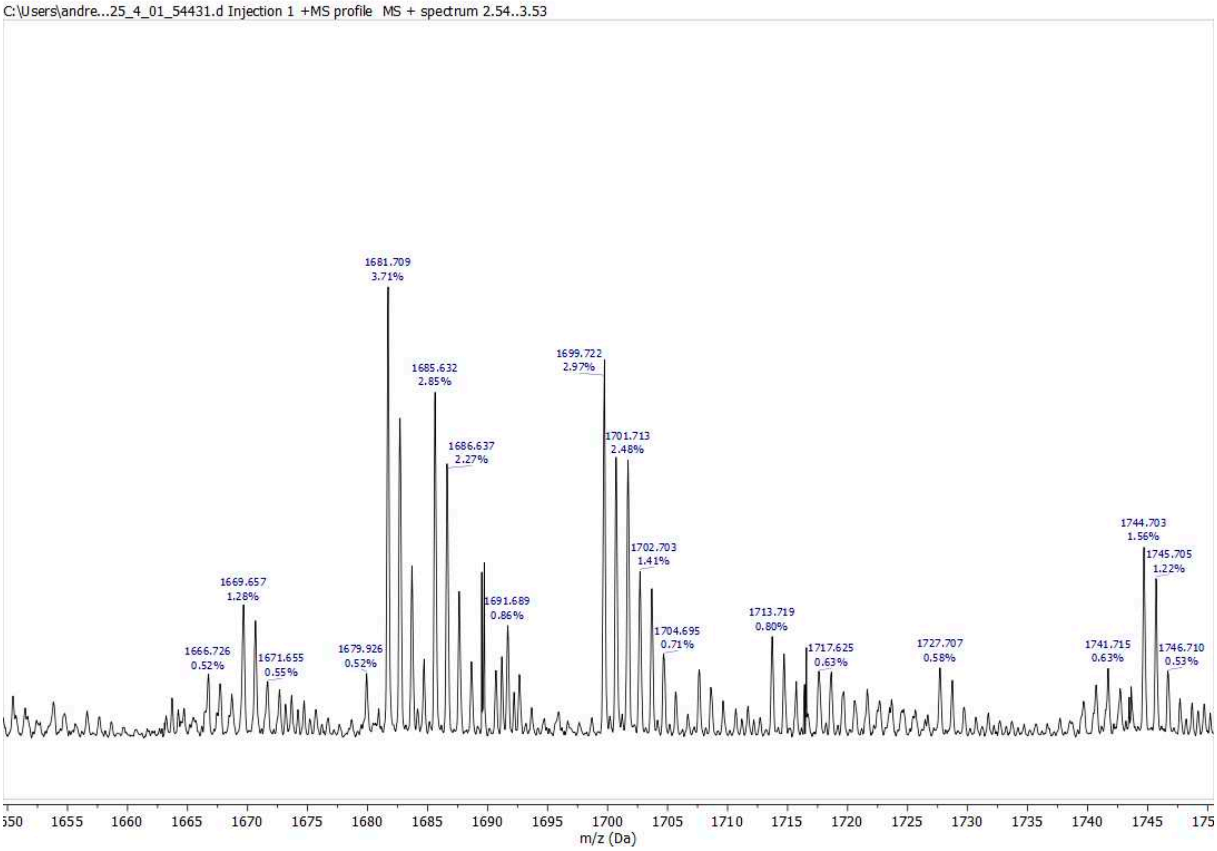

Figure S66: Excerpt of the ESI(+)-MS of [$(UO_2)_2(H_2L3)(MeO)_2$] (**8**).

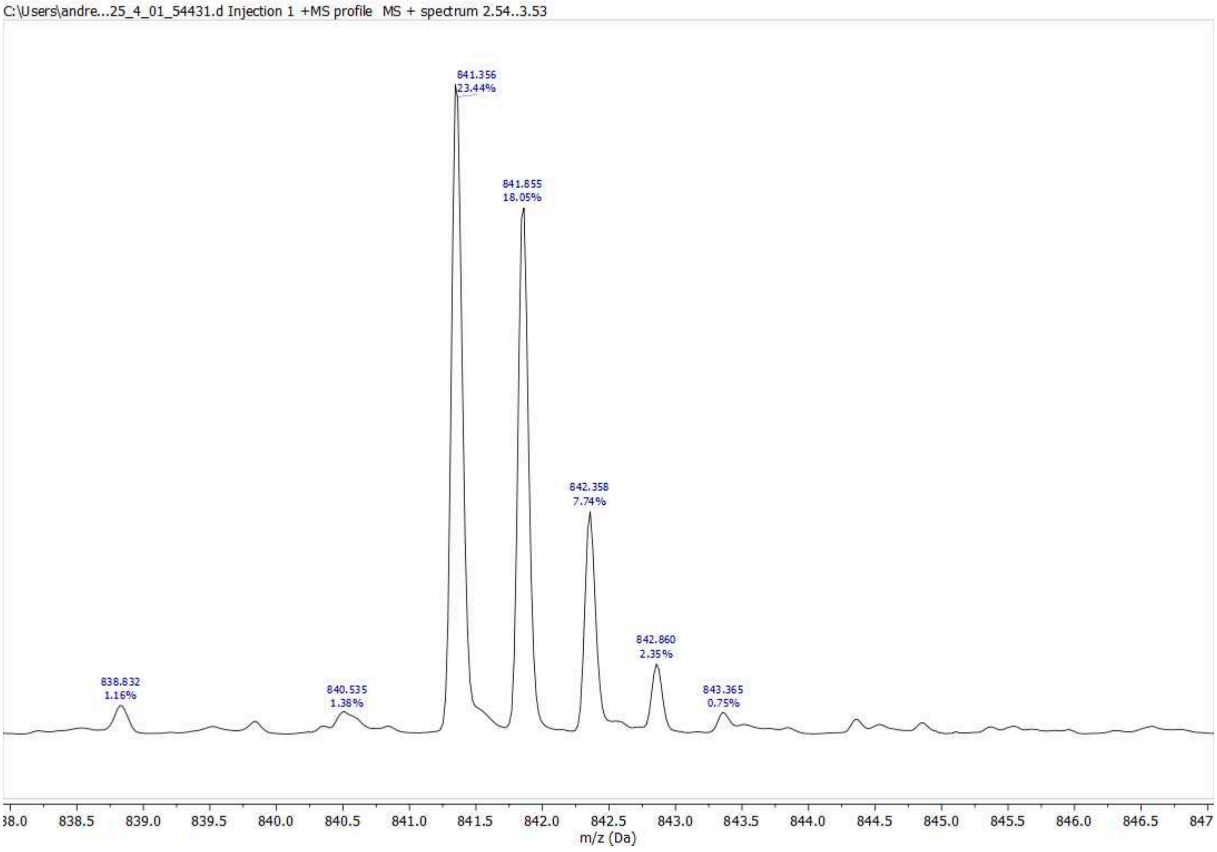

Figure S67: Excerpt of the ESI(+)-MS of [$(UO_2)_2(H_2L3)(MeO)_2$] (**8**).

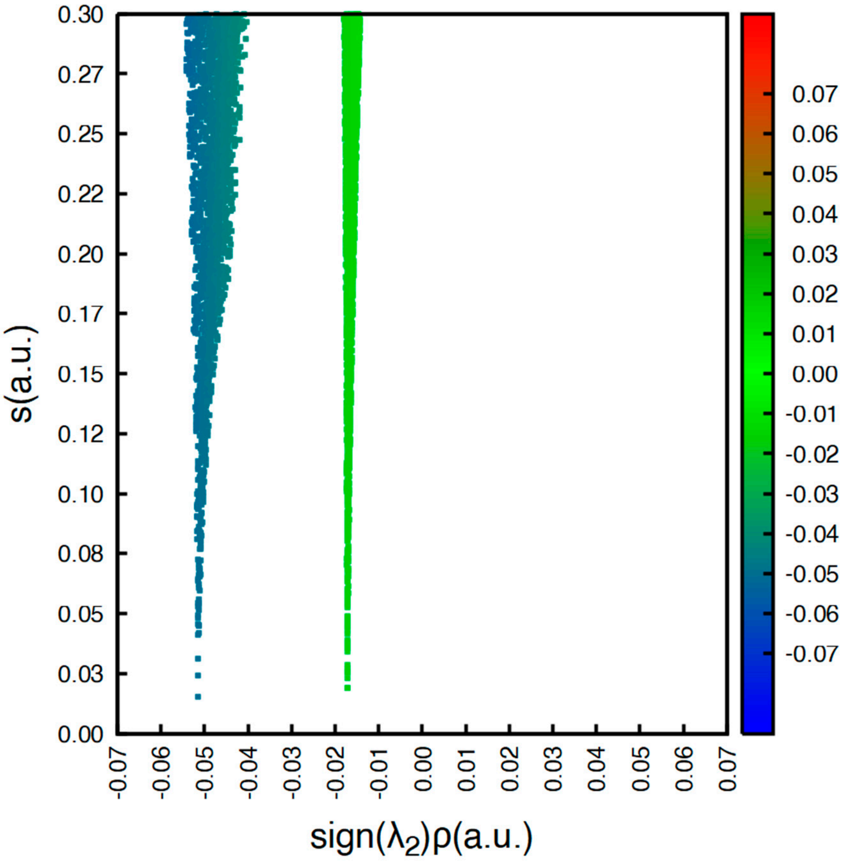

Figure S68: The resulting fingerprint plot for the calculated NCI plot of [$(UO_2)(H_2L1)$] (**6**).

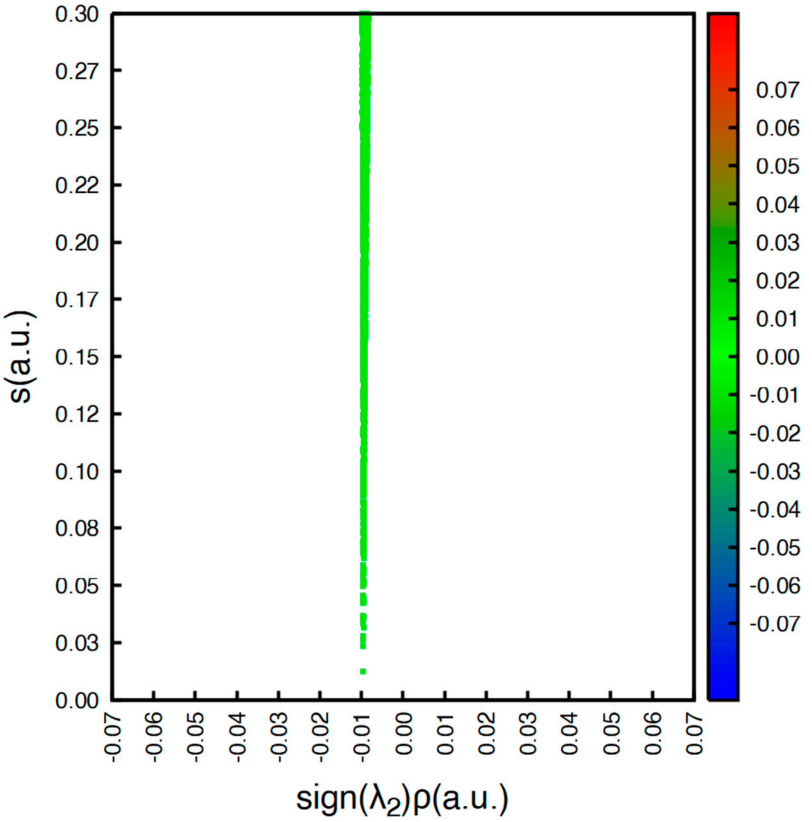

Figure S69: Fingerprint plot for the calculated NCI plot for [$(UO_2)_2(H_2L3)(MeO)_2$] (**8**).

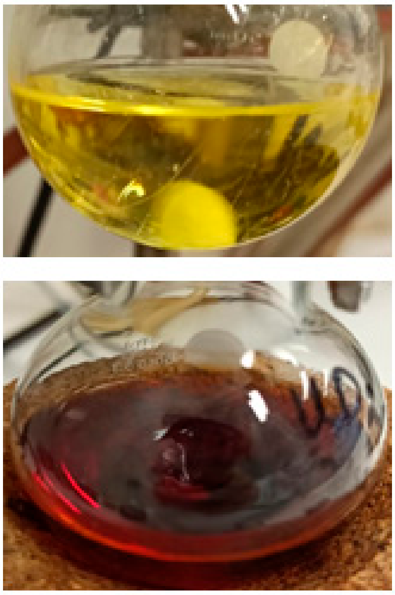

Figure S70: Color change upon $UO_2(NO_3)_2$ * 6 H_2O addition to a solution of the Ligand H_4L2 in DCM / MeOH with 4.4 eq. NEt_3.

3. Single Crystal X-Ray crystallography

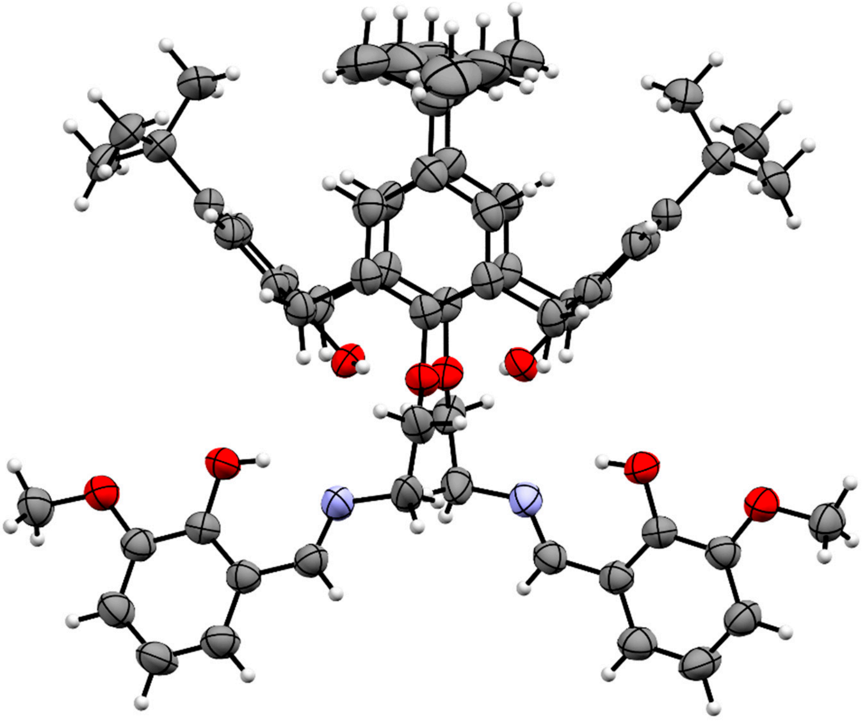

Figure S71: ORTEP representation of H_4L1. Thermal ellipsoids drawn at 50% probability. Color code: C, grey; H, white; N, light blue; O, red. Solvent molecules have been omitted for clarity.

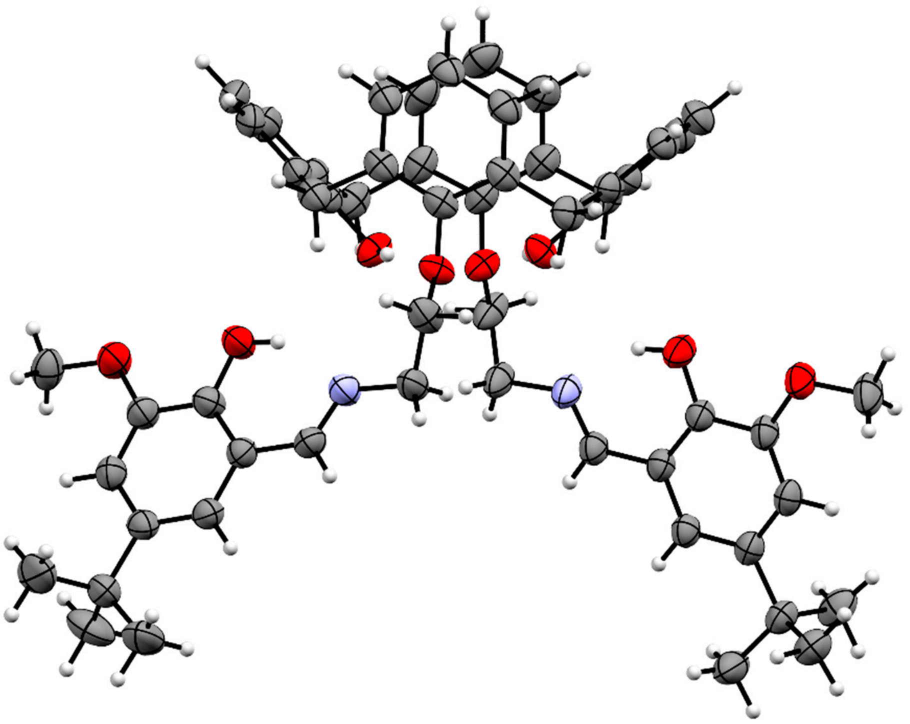

Figure S72: ORTEP representation of H_4L2. Thermal ellipsoids drawn at 50% probability. Color code: C, grey; H, white; N, light blue; O, red. Solvent molecules have been omitted for clarity.

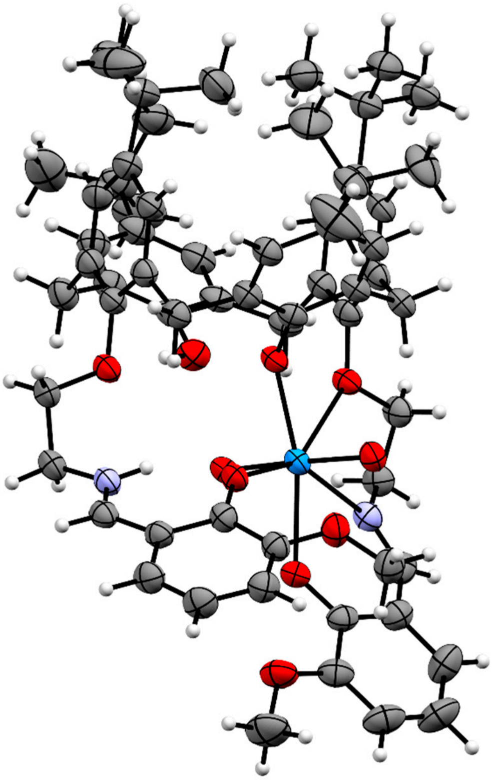

Figure S73: ORTEP representation of $[(UO_2)(H_2L1)]$ (**6**). Thermal ellipsoids drawn at 50% probability. Color code: C, grey; H, white; N, light blue; O, red; U. deep blue. Solvent molecules have been omitted for clarity.

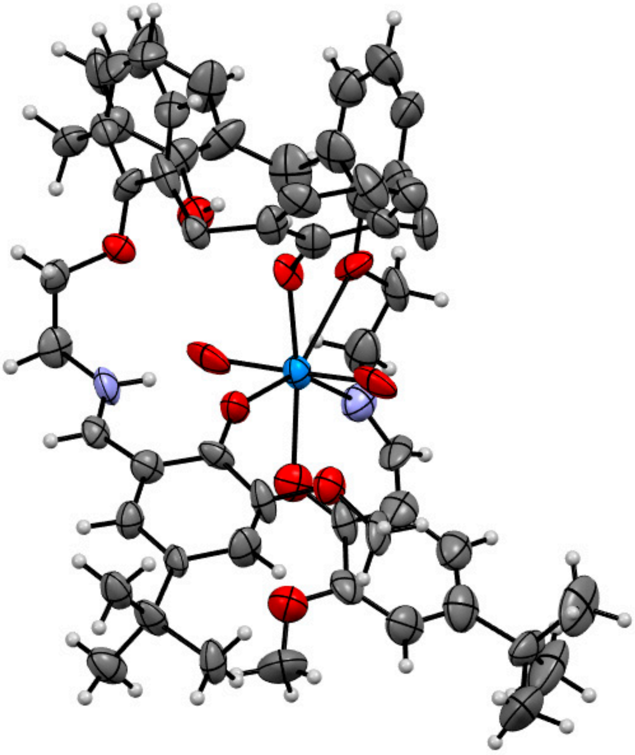

Figure S74: ORTEP representation of [$(UO_2)(H_2L2)$] (**7**). Thermal ellipsoids drawn at 50% probability. Color code: C, grey; H, white; N, light blue; O, red; U. deep blue. Solvent molecules have been omitted for clarity.

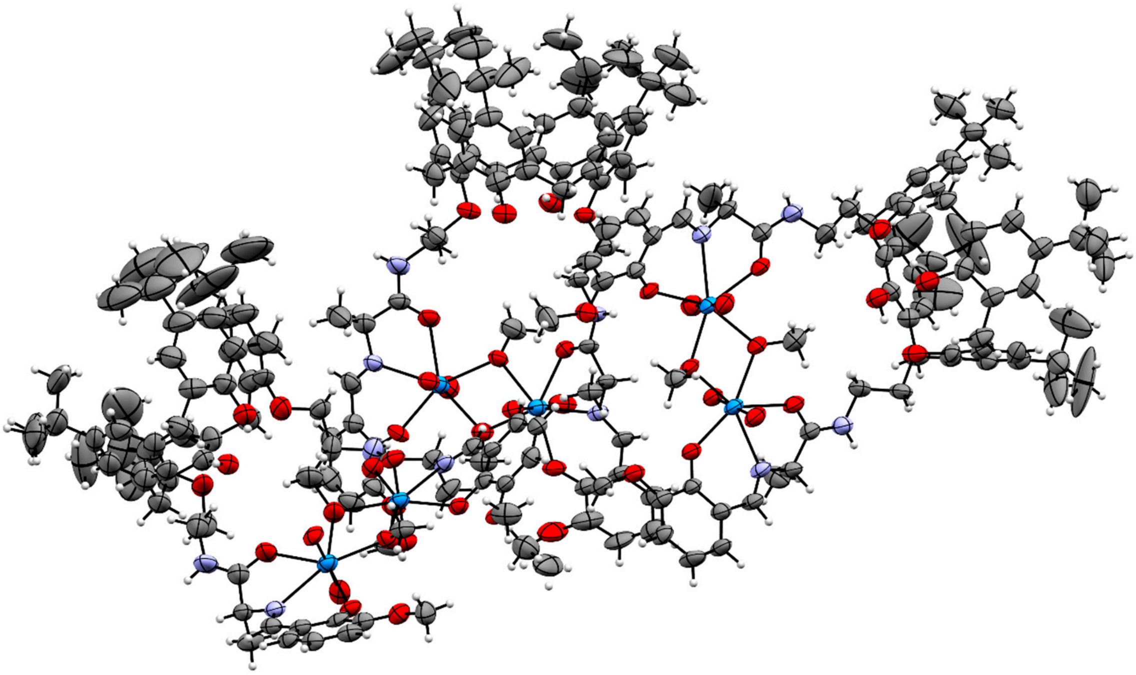

Figure S75: ORTEP representation of [$(UO_2)_2(H_2L3)(MeO)_2$] (**8**). Thermal ellipsoids drawn at 50% probability. Color code: C, grey; H, white; N, light blue; O, red; U. deep blue. Solvent molecules have been omitted for clarity.

Table S1: Crystal data and structure refinement for the reported structures.

Identification code	sv-1991, H_4L1	sv-2303_3d, H_4L2	sv-2895, **6**	sv-2286_3d, **7**	sv-2414, **8**
CCDC	2533613	2533614	2533616	2533615	2533617
Chemical Formula	$C_{64}H_{78}N_2O_8$	$C_{56}H_{62}N_2O_8$<br>$\cdot C_2H_4O$<br>$\cdot CH_4O$	$C_{64}H_{76}N_2O_{10}U$<br>$\cdot 2.75[CH_2Cl_2]$<br>$\cdot CH_4O$	$C_{56}H_{59}N_2O_{10}U$<br>$\cdot 0.6[CH_2Cl_2]$<br>$\cdot 0.4[CH_2Cl_2]$	$C_{72}H_{92}N_4O_{16}U_2$<br>$\cdot CH_4O \cdot 13[CH_4O]$
M_r	1003.28	967.17	1536.88	1244.01	2194.14
Crystal System, space group	Monoclinic, *C*2/*c*	Triclinic, *P*-1	Monoclinic, *I*2/*a*	Monoclinic, *C*2/*c*	Triclinic, *P*-1
Temperature (K)	180	180	130	180	130
a, b, c (Å)	26.119 (11),<br>21.304 (14),<br>10.372 (5)	10.4894 (4),<br>14.4854 (6),<br>18.5497 (8)	44.915 (1),<br>10.4442 (3),<br>31.5971 (7)	44.560,<br>11.200,<br>43.798	21.841 (3),<br>23.009 (4),<br>32.982 (5)
α, β, γ (°)	90,<br>93.80 (4),<br>90	95.217 (3),<br>93.857 (3),<br>103.565 (3)	90,<br>108.680 (2),<br>90	90,<br>97.53,<br>90	89.618 (12),<br>87.709 (12),<br>84.273 (12)
V (Å^3)	5758 (5)	2717.1 (2)	14041.4 (6)	21669.86	16479 (4)
Z	4	2	8	16	6
Radiation Type	Cu $K\alpha$				
μ (mm^{-1})	0.60	0.64	8.90	9.82	8.80
Crystal size (mm) (radius)	0.14 × 0.10 × 0.08 × 0.20	0.24	0.22 × 0.15 × 0.1 × 0.09	0.25 × 0.17 × 0.11 × 0.08	0.12 × 0.08 × 0.06 × 0.19
Data collection Diffractometer Absorption correction	STOE STADIVARI Absorption correction Multi-scan STOE *X-RED32*, absorption correction by Gaussian integration, analogous to P. Coppens in: F. R. Ahmed (Editor), "Crystallographic Computing", Munksgaard, Copenhagen (1970), 255 - 270. Afterwards scaling of reflection intensities was performed within STOE LANA. J. Koziskova, F. Hahn, J. Richter, J. Kozisek, Acta Chimica Slovaca, vol. 9, no. 2, 2016, pp. 136 - 140. Finally a spherical absorption correction was done within STOE LANA.				
T_{min}, T_{max}	0.444, 0.717	0.337, 0.596	0.011, 0.039	0.017, 0.075	1.000, 1.000
No. of measured, independent and observed [I > 2σ(I)] reflections	25172, 5479, 3897	26214, 9514, 5669	54180, 13887, 12450	70159, 19598, 11412	90671, 56416, 40314
R_{int}	0.032	0.058	0.030	0.070	0.064
$(\sin \theta/\lambda)_{max}$ (Å^{-1})	0.612	0.599	0.621	0.601	0.605
$R[F2 > 2\sigma(F2)]$, $wR(F2)$, S	0.050, 0.155, 1.05	0.065, 0.212, 1.01	0.043, 0.120, 1.06	0.114, 0.349, 1.21	0.055, 0.155, 0.98
No. of reflections	5479	9514	13887	19598	56416
No. of parameters	374	705	838	1248	2659
No. of restraints	0	29	44	0	260
H-Atom treatment	H-atom parameters constrained $w = 1/[\sigma^2(F_o^2) + (0.0811P)^2 + 1.8053P]$ where $P = (F_o^2 + 2F_c^2)/3$	H-atom parameters constrained $w = 1/[\sigma^2(F_o^2) + (0.125P)^2]$ where $P = (F_o^2 + 2F_c^2)/3$	H-atom parameters constrained $w = 1/[\sigma^2(F_o^2) + (0.0825P)^2 + 33.1375P]$ where $P = (F_o^2 + 2F_c^2)/3$	H-atom parameters constrained $w = 1/[\sigma^2(F_o^2) + (0.2P)^2]$ where $P = (F_o^2 + 2F_c^2)/3$	H-atom parameters constrained $w = 1/[\sigma^2(F_o{}^2) + (0.0943P)^2]$ where $P = (F_o^2 + 2F_c^2)/3$
$\Delta\rho_{max}, \Delta\rho_{min}$ (e Å^{-3})	0.19, −0.18	0.57, −0.24	3.91, −0.89	11.92, −1.74	2.92, −1.61

4. Comparison of optimized and experimentally determined structures of 6 and 8

Table S2: xyz coordinates of the crystal structure and the dft optimized structure of **6**.

		XRD			OPT		
No	Label	X	Y	Z	X	Y	Z
1	C	27.2485	2.3771	7.2338	27.1222	2.5061	7.1473
2	C	27.1979	1.5875	5.9407	27.0676	1.7559	5.8421
3	H	26.4469	1.9156	5.3857	26.2553	2.1204	5.2168
4	H	28.0371	1.733	5.4355	27.9981	1.9299	5.2952
5	C	27.0216	0.117	6.1967	26.9272	0.2793	6.0958
6	C	28.0436	-0.799	5.9222	27.9705	-0.5916	5.8106
7	H	28.8392	-0.4793	5.5125	28.849	-0.179	5.3275
8	C	27.9499	-2.1484	6.2194	27.9194	-1.9449	6.1189
9	C	26.7978	-2.5609	6.8905	26.7768	-2.3944	6.7754
10	H	26.7295	-3.4694	7.1602	26.7017	-3.439	7.0654
11	C	25.7419	-1.6982	7.1823	25.7214	-1.5582	7.0956
12	C	24.5757	-2.163	8.0213	24.5846	-2.0397	7.9535
13	H	24.5116	-3.1507	7.9913	24.5213	-3.1307	7.8912
14	H	23.7328	-1.7862	7.6641	23.6459	-1.6252	7.5929
15	C	24.7837	-1.6961	9.4734	24.8128	-1.6282	9.3906
16	C	25.9805	-1.9604	10.1181	25.9882	-1.9865	10.0309
17	H	26.6193	-2.5125	9.6823	26.69	-2.5986	9.4768
18	C	26.2881	-1.4517	11.3816	26.3109	-1.5598	11.3146
19	C	25.3361	-0.6559	11.9949	25.4156	-0.7136	11.9537
20	H	25.5247	-0.295	12.8531	25.6451	-0.3082	12.9315
21	C	24.1026	-0.3655	11.3948	24.2149	-0.3388	11.3619
22	C	23.1445	0.6235	12.0473	23.3086	0.6504	12.0569
23	H	22.2885	0.6343	11.55	22.3192	0.6488	11.5927
24	H	22.9567	0.3398	12.9771	23.1666	0.3438	13.0958
25	C	23.7453	2.0136	12.053	23.8789	2.0437	12.0427
26	C	24.1352	2.6131	13.2578	24.3263	2.6346	13.2215
27	H	23.9637	2.155	14.0722	24.2002	2.0713	14.1387
28	C	24.7649	3.8529	13.3072	24.9129	3.8903	13.2542
29	C	25.0199	4.4847	12.088	25.0659	4.5462	12.0331
30	H	25.456	5.329	12.0927	25.5462	5.5195	12.0013
31	C	24.6592	3.9249	10.8685	24.6489	3.9926	10.8383
32	C	25.0702	4.537	9.5518	24.9676	4.6129	9.5079
33	H	25.3299	5.4814	9.6929	25.2198	5.6686	9.6475
34	H	24.3036	4.5191	8.9253	24.0995	4.5538	8.852
35	C	26.2436	3.7693	8.9442	26.1332	3.8757	8.8842
36	C	27.4343	3.6795	9.6721	27.2885	3.7167	9.6295

37	H	27.4905	4.1295	10.5072	27.3157	4.1685	10.6141
38	C	28.539	2.962	9.2288	28.3708	2.9678	9.1851
39	C	28.4104	2.3029	8.0178	28.2516	2.3586	7.9476
40	H	29.1383	1.779	7.7048	29.0457	1.726	7.5701
41	C	26.1869	3.1259	7.7008	26.0756	3.2814	7.6244
42	C	24.001	2.6977	10.8596	24.0207	2.7431	10.8473
43	C	23.8462	-0.917	10.1391	23.9192	-0.8306	10.0973
44	C	25.828	-0.3541	6.7884	25.7653	-0.2066	6.7118
45	C	25.043	4.5923	6.2233	24.9272	4.7686	6.2203
46	H	25.5688	4.5297	5.3867	25.5228	4.7345	5.3003
47	H	25.4688	5.2694	6.8065	25.3685	5.5078	6.894
48	C	23.6255	5.0101	5.9057	23.4934	5.1268	5.9237
49	H	23.1048	5.104	6.7425	22.9405	5.1725	6.8684
50	H	23.6265	5.8836	5.4396	23.4603	6.1227	5.4616
51	C	22.5633	4.4001	3.9263	22.409	4.5013	3.9576
52	H	22.7313	5.3095	3.7089	22.5094	5.5592	3.6827
53	C	21.8234	3.6314	2.9582	21.7171	3.7016	2.9876
54	C	21.2151	2.4043	3.2815	21.2156	2.4215	3.3121
55	C	20.2975	1.8403	2.3554	20.44	1.7521	2.3209
56	C	20.0859	2.4387	1.1309	20.2324	2.3227	1.0849
57	H	19.4987	2.0309	0.5057	19.6528	1.797	0.3366
58	C	20.7187	3.6294	0.7962	20.7495	3.5897	0.7803
59	H	20.5659	4.0273	-0.0514	20.5678	4.0213	-0.1959
60	C	21.5727	4.2362	1.7071	21.4639	4.2722	1.7268
61	H	21.9901	5.0609	1.4871	21.8526	5.2629	1.5147
62	C	18.6961	0.1243	1.9336	19.1139	-0.1272	1.8168
63	H	19.0672	0.0216	1.0327	19.6347	-0.397	0.8896
64	H	17.9125	0.712	1.8994	18.2317	0.4723	1.5638
65	H	18.4311	-0.7534	2.28	18.8038	-1.0387	2.3245
66	C	21.5726	-1.5186	9.8415	21.6407	-1.2954	9.8348
67	H	21.2713	-1.3796	10.7744	21.3743	-1.1998	10.8954
68	H	21.8965	-2.4496	9.7528	21.916	-2.34	9.6456
69	C	20.43	-1.2742	8.8864	20.464	-0.8968	8.9721
70	H	19.6968	-1.9119	9.0744	19.5883	-1.493	9.239
71	H	20.0805	-0.3563	9.0137	20.2335	0.16	9.1305
72	C	20.3952	-2.2737	6.6723	20.2615	-1.9903	6.7963
73	H	19.7448	-2.8924	6.9818	19.4627	-2.6051	7.2079
74	C	20.7743	-2.3291	5.2933	20.6672	-2.2045	5.4692
75	C	21.7766	-1.4643	4.7692	21.7376	-1.433	4.9278
76	C	21.9645	-1.4925	3.3566	22.0705	-1.6483	3.552
77	C	21.2507	-2.3531	2.5625	21.4049	-2.597	2.8198
78	H	21.422	-2.3752	1.6285	21.6689	-2.7555	1.7816
79	C	20.2809	-3.1938	3.0995	20.3685	-3.3711	3.3871
80	H	19.7906	-3.7785	2.534	19.8708	-4.1148	2.7775
81	C	20.0371	-3.174	4.4357	19.9989	-3.1755	4.6818
82	H	19.3624	-3.7361	4.7977	19.1963	-3.7534	5.1273

83	C	22.9748	-0.3812	1.5227	23.3256	-0.8337	1.7349
84	H	23.3097	-1.1914	1.0844	23.7276	-1.7957	1.3973
85	H	23.5847	0.3642	1.3403	24.0738	-0.0571	1.5917
86	H	22.0839	-0.1666	1.1759	22.4298	-0.5877	1.1538
87	C	29.836	2.963	10.0544	29.6087	2.8425	10.062
88	C	29.5373	2.7771	11.5597	29.203	2.353	11.4556
89	H	29.1112	1.9062	11.7029	28.6814	1.3961	11.3941
90	H	28.9366	3.4888	11.8638	28.5379	3.0574	11.9579
91	H	30.3753	2.8179	12.0663	30.089	2.2234	12.0837
92	C	30.5398	4.3176	9.8897	30.2722	4.2175	10.1857
93	H	31.37	4.3191	10.4109	31.1611	4.1582	10.8208
94	H	29.9511	5.0321	10.2121	29.5907	4.9488	10.6259
95	H	30.7466	4.4657	8.9443	30.5768	4.5911	9.2049
96	C	30.8259	1.8747	9.6174	30.6244	1.8638	9.4827
97	H	31.6187	1.9035	10.1924	31.4807	1.7813	10.1566
98	H	31.092	2.0295	8.6865	30.9998	2.197	8.512
99	H	30.3994	0.9954	9.6925	30.1988	0.8653	9.3581
100	C	29.0374	-3.1698	5.8698	29.0491	-2.9162	5.8033
101	C	29.598	-3.7735	7.1449	29.5806	-3.5229	7.1063
102	H	30.3911	-4.3086	6.933	30.3958	-4.2236	6.9013
103	H	28.9198	-4.3466	7.5613	28.8004	-4.0668	7.6432
104	H	29.8441	-3.0563	7.7658	29.9603	-2.7408	7.7685
105	C	30.1602	-2.5526	5.0556	30.2121	-2.2351	5.09
106	H	30.5949	-1.8476	5.5785	30.6547	-1.4464	5.7034
107	H	29.7931	-2.168	4.2312	29.9001	-1.7952	4.1396
108	H	30.8176	-3.2431	4.8276	30.9938	-2.969	4.8779
109	C	28.4342	-4.2999	5.0526	28.5241	-4.0376	4.9016
110	H	27.9916	-3.9293	4.2601	28.1499	-3.6308	3.9591
111	H	27.7786	-4.7832	5.5967	27.7055	-4.5825	5.3762
112	H	29.1438	-4.915	4.772	29.3194	-4.7545	4.6752
113	C	27.6959	-1.6794	11.9551	27.6082	-2.0325	11.9583
114	C	27.8747	-1.0423	13.2901	27.8848	-1.3241	13.2804
115	H	27.7089	-0.0788	13.218	27.9559	-0.2413	13.1525
116	H	28.79	-1.1932	13.6056	28.8346	-1.6747	13.6915
117	H	27.2412	-1.4346	13.928	27.1097	-1.5316	14.0223
118	C	28.6854	-1.0152	10.9733	28.7869	-1.7736	11.0156
119	H	28.6526	-1.4802	10.1127	28.6906	-2.3223	10.0777
120	H	29.5929	-1.0671	11.3399	29.7216	-2.0867	11.489
121	H	28.4378	-0.0749	10.8476	28.8663	-0.7131	10.771
122	C	28.0295	-3.1688	12.006	27.5019	-3.5374	12.2252
123	H	27.463	-3.6087	12.6735	26.6725	-3.7545	12.903
124	H	28.9716	-3.2846	12.2491	28.4236	-3.91	12.6814
125	H	27.8679	-3.5692	11.1258	27.3336	-4.0924	11.2997
126	C	25.2284	4.4743	14.6409	25.416	4.5415	14.5356
127	C	24.6043	3.8215	15.8493	25.1234	3.691	15.7668
128	H	24.7593	2.855	15.8183	25.6216	2.7196	15.7165

129	H	23.6375	3.9958	15.8525	24.0513	3.521	15.8936
130	H	25.0024	4.191	16.665	25.4874	4.2021	16.6614
131	C	24.8642	5.9762	14.6939	24.7389	5.9024	14.7224
132	H	25.227	6.3719	15.5134	25.0989	6.385	15.6356
133	H	23.8895	6.0766	14.6858	23.6549	5.7887	14.7988
134	H	25.2458	6.4324	13.9143	24.9455	6.5729	13.886
135	C	26.7431	4.3176	14.7388	26.9323	4.7399	14.4392
136	H	27.0579	4.7035	15.5822	27.3151	5.2215	15.3438
137	H	27.1682	4.7838	13.9877	27.201	5.3679	13.587
138	H	26.9763	3.3668	14.7061	27.4415	3.7802	14.3211
139	N	23.018	3.9855	5.0496	22.8882	4.1238	5.0833
140	N	20.881	-1.4382	7.514	20.775	-1.0904	7.5825
141	H	21.5396	-0.9237	7.2399	21.5237	-0.5287	7.1567
142	O	25.0326	3.3025	6.8944	24.9445	3.4855	6.8378
143	O	24.7894	0.4648	7.0012	24.7391	0.585	6.9699
144	O	22.6273	-0.5964	9.5213	22.7232	-0.4516	9.5057
145	O	23.6305	2.2382	9.6239	23.6092	2.2977	9.6498
146	H	23.3423	1.4516	9.6927	23.1813	1.4306	9.6681
147	O	21.3957	1.8256	4.457	21.3856	1.8785	4.4665
148	O	19.6878	0.6998	2.7972	19.9562	0.5509	2.7088
149	O	22.471	-0.6632	5.5423	22.3763	-0.5897	5.6354
150	O	22.8979	-0.5995	2.9298	23.044	-0.8466	3.11
151	O	22.2038	1.7181	7.1683	22.1408	1.776	7.1975
152	O	24.3403	1.4403	4.3417	24.2216	1.6147	4.354
153	U	23.2884	1.5246	5.771	23.2062	1.655	5.7898

Table S3: xyz coordinates of the crystal structure and the dft calculated structure of **8**.

		XRD			Calc		
No	Label	X	Y	Z	X	Y	Z
1	U	1.9934	11.4975	23.4116	1.9934	11.4976	23.4116
2	U	4.0386	9.8187	26.1383	4.0386	9.8187	26.1383
3	O	3.6828	11.8954	22.8082	3.6828	11.8954	22.8082
4	O	2.9033	9.4747	24.1959	2.9033	9.4747	24.1959
5	O	1.8179	13.8515	23.9811	1.8179	13.8515	23.9811
6	O	1.4556	10.3034	21.6188	1.4556	10.3034	21.6188
7	O	1.9962	17.9954	26.5839	1.9962	17.9954	26.5839
8	O	2.7171	11.6948	25.6691	2.717	11.6948	25.669
9	O	5.4847	10.6706	25.3507	5.4847	10.6706	25.3507
10	O	5.0947	7.949	25.5372	5.0947	7.949	25.5372
11	O	4.3245	11.2205	28.0748	4.3245	11.2205	28.0748
12	O	4.9893	5.8817	23.8697	4.9893	5.8817	23.8697
13	O	4.3078	15.3906	29.3304	4.3078	15.3906	29.3304
14	O	4.8896	17.2302	27.2377	4.8896	17.2303	27.2377

15	H	4.3415	16.9655	27.8168	4.3415	16.9656	27.8168
16	O	1.5579	16.5315	28.757	1.5579	16.5315	28.757
17	H	2.3145	16.8869	28.669	2.3145	16.8869	28.669
18	N	1.9467	16.0663	23.5006	1.9467	16.0663	23.5006
19	H	1.8632	16.6949	22.8896	1.8632	16.6949	22.8896
20	O	2.7248	8.9326	26.9543	2.7248	8.9326	26.9543
21	O	0.271	11.1707	24.0279	0.271	11.1707	24.0279
22	N	1.1004	13.0575	21.6155	1.1004	13.0575	21.6155
23	C	1.5075	14.4887	21.7078	1.5075	14.4887	21.7078
24	H	0.7771	15.0716	21.3497	0.7771	15.0716	21.3497
25	C	0.3295	12.8143	20.5807	0.3295	12.8143	20.5807
26	H	0.0648	13.5401	20.029	0.0648	13.5401	20.029
27	C	4.9849	10.9783	29.0898	4.9849	10.9783	29.0898
28	C	1.5895	17.7354	25.2505	1.5895	17.7354	25.2505
29	H	1.8627	18.4763	24.6523	1.8627	18.4763	24.6523
30	H	0.6069	17.6323	25.2005	0.6069	17.6322	25.2005
31	N	5.0085	11.8143	30.1016	5.0085	11.8143	30.1016
32	H	5.5121	11.6265	30.7983	5.5121	11.6265	30.7983
33	C	2.7811	14.6879	20.8806	2.7811	14.6879	20.8806
34	H	3.4459	14.0162	21.14	3.4459	14.0161	21.14
35	H	3.14	15.584	21.0436	3.14	15.584	21.0436
36	H	2.5698	14.5875	19.9288	2.5698	14.5875	19.9288
37	N	5.432	8.8259	28.1308	5.432	8.8259	28.1308
38	C	5.8698	9.7627	29.1426	5.8698	9.7627	29.1426
39	H	5.7688	9.3341	30.0392	5.7688	9.3341	30.0392
40	C	1.1119	16.6613	31.0573	1.1119	16.6613	31.0573
41	C	3.5304	16.1771	31.4725	3.5304	16.1771	31.4725
42	C	1.7489	14.8154	23.1644	1.7489	14.8154	23.1644
43	C	0.0336	18.2462	29.6006	0.0336	18.2462	29.6007
44	C	1.9226	19.3609	26.9477	1.9226	19.3609	26.9477
45	C	-0.1408	11.4581	20.2479	-0.1408	11.4581	20.2479
46	C	4.532	16.1012	30.4871	4.532	16.1012	30.4871
47	C	7.1647	19.3898	29.1887	7.1647	19.3898	29.1887
48	H	7.794	19.3271	29.898	7.794	19.3271	29.898
49	C	5.5364	18.3579	27.6958	5.5363	18.3579	27.6958
50	C	6.8927	20.6634	28.5823	6.8927	20.6634	28.5823
51	C	0.8395	19.8231	27.709	0.8395	19.8231	27.709
52	C	-0.1984	18.8629	28.1967	-0.1984	18.8629	28.1967
53	H	-0.2681	18.1236	27.5436	-0.2681	18.1237	27.5436
54	H	-1.0711	19.331	28.2081	-1.0711	19.331	28.2081
55	C	2.1677	15.579	31.2814	2.1677	15.579	31.2814
56	H	1.9287	15.0449	32.08	1.9287	15.0449	32.08
57	H	2.1833	14.9691	30.5003	2.1833	14.9691	30.5003
58	C	5.295	19.5787	27.0598	5.295	19.5787	27.0598
59	C	5.1207	6.6774	26.048	5.1207	6.6774	26.048
60	C	0.8707	17.1592	29.7753	0.8707	17.1592	29.7753

61	C	5.2712	6.4957	27.4355	5.2711	6.4957	27.4355
62	C	-0.4157	18.2916	31.9767	-0.4157	18.2916	31.9767
63	C	2.8297	21.5631	26.8489	2.8297	21.5631	26.8489
64	H	3.5008	22.1718	26.5641	3.5008	22.1718	26.5641
65	C	-1.1462	8.9879	19.4306	-1.1462	8.9879	19.4306
66	H	-1.5503	8.1556	19.2166	-1.5503	8.1556	19.2166
67	C	5.2914	5.151	27.9858	5.2914	5.151	27.9858
68	H	5.4178	5.0045	28.9164	5.4178	5.0045	28.9164
69	C	5.0417	5.5533	25.1945	5.0417	5.5533	25.1945
70	C	6.4913	18.2141	28.7306	6.4913	18.2141	28.7306
71	C	4.218	13.0575	30.1016	4.218	13.0575	30.1016
72	H	4.0634	13.3632	31.031	4.0634	13.3632	31.031
73	H	3.3401	12.9047	29.6717	3.3401	12.9047	29.6717
74	C	-1.1182	11.4051	19.2559	-1.1182	11.4051	19.2559
75	H	-1.4417	12.2059	18.8606	-1.4417	12.2059	18.8606
76	C	5.5726	7.5925	28.3516	5.5726	7.5925	28.3516
77	H	5.9108	7.3489	29.2046	5.9108	7.3489	29.2046
78	C	0.482	17.2389	32.1613	0.482	17.2389	32.1613
79	H	0.6649	16.9167	33.0349	0.6649	16.9167	33.0349
80	C	-0.6227	18.7826	30.6948	-0.6227	18.7826	30.6948
81	H	-1.2269	19.5036	30.5672	-1.2269	19.5036	30.5672
82	C	5.7303	16.7815	30.619	5.7303	16.7815	30.619
83	C	1.7403	22.0594	27.531	1.7403	22.0594	27.531
84	C	0.8157	21.1518	27.9891	0.8157	21.1517	27.9891
85	H	0.1128	21.4762	28.5413	0.1128	21.4762	28.5413
86	C	5.9865	17.5035	31.7823	5.9865	17.5035	31.7823
87	H	6.8029	17.9769	31.8865	6.8029	17.9769	31.8865
88	C	5.0144	17.5131	32.7973	5.0144	17.5131	32.7973
89	C	6.0334	20.7018	27.5277	6.0334	20.7018	27.5277
90	H	5.9179	21.5298	27.0782	5.9179	21.5298	27.0782
91	C	3.8258	16.8716	32.6457	3.8258	16.8716	32.6457
92	H	3.1859	16.8948	33.3479	3.1859	16.8948	33.3479
93	C	2.9926	20.1886	26.5556	2.9926	20.1886	26.5556
94	C	-1.6129	10.1837	18.8473	-1.6129	10.1837	18.8473
95	H	-2.2726	10.147	18.1636	-2.2726	10.147	18.1636
96	C	5.0253	4.2841	25.7317	5.0253	4.2841	25.7317
97	H	4.9481	3.5338	25.1538	4.9481	3.5338	25.1538
98	C	4.9948	14.0925	29.3403	4.9948	14.0925	29.3403
99	H	5.8886	14.1994	29.7516	5.8886	14.1994	29.7516
100	H	5.123	13.7841	28.408	5.123	13.7841	28.408
101	C	0.4177	10.2919	20.7587	0.4177	10.2919	20.7587
102	C	4.2832	19.6971	25.9788	4.2832	19.6971	25.9788
103	H	4.1458	18.8144	25.552	4.1458	18.8144	25.552
104	H	4.6044	20.329	25.289	4.6044	20.329	25.289
105	C	4.9903	4.7733	22.9008	4.9903	4.7733	22.9008
106	H	4.9109	5.1314	21.9931	4.9109	5.1314	21.9931

107	H	4.2346	4.1772	23.0854	4.2346	4.1772	23.0854
108	H	5.8288	4.27	22.9813	5.8288	4.27	22.9813
109	C	2.2941	16.4271	24.8485	2.2941	16.4271	24.8485
110	H	3.2749	16.5428	24.9178	3.2749	16.5428	24.9178
111	H	2.025	15.702	25.4661	2.025	15.702	25.4661
112	O	0.4535	7.8975	20.8872	0.4535	7.8975	20.8872
113	C	5.1157	4.0772	27.0993	5.1157	4.0772	27.0993
114	H	5.06	3.1945	27.4446	5.0599	3.1945	27.4446
115	C	-1.1459	18.881	33.2192	-1.1459	18.881	33.2192
116	C	7.299	10.1858	28.9745	7.299	10.1858	28.9745
117	H	7.4724	10.3902	28.032	7.4724	10.3902	28.032
118	H	7.8913	9.462	29.2665	7.8913	9.462	29.2665
119	H	7.4696	10.9852	29.5176	7.4696	10.9852	29.5176
120	C	1.9113	12.2949	26.6314	1.9113	12.2949	26.6314
121	H	2.3253	13.1302	26.9308	2.3253	13.1302	26.9308
122	H	1.8097	11.6883	27.3956	1.8097	11.6883	27.3956
123	H	1.0301	12.4849	26.2473	1.0301	12.4849	26.2473
124	C	6.5415	19.2631	33.9969	6.5415	19.2631	33.9969
125	H	7.2224	18.8282	33.4427	7.2224	18.8282	33.4427
126	H	6.9119	19.452	34.8833	6.9119	19.452	34.8833
127	H	6.2608	20.1007	33.5733	6.2607	20.1007	33.5733
128	C	6.7382	16.8883	29.4952	6.7382	16.8883	29.4952
129	H	6.64	16.1192	28.8784	6.64	16.1192	28.8784
130	H	7.6573	16.8747	29.8641	7.6573	16.8747	29.8641
131	C	2.2796	8.1895	23.8927	2.2796	8.1895	23.8927
132	H	1.8451	8.2364	23.0162	1.8451	8.2364	23.0162
133	H	1.6092	7.9815	24.5784	1.6092	7.9815	24.5784
134	H	2.9633	7.4882	23.8827	2.9633	7.4882	23.8827
135	C	7.6625	21.8669	29.1426	7.6625	21.8669	29.1426
136	C	0.5043	23.9101	28.6911	0.5043	23.9101	28.6911
137	H	-0.3322	23.9257	28.1781	-0.3322	23.9257	28.1781
138	H	0.6447	24.7853	29.1096	0.6447	24.7854	29.1096
139	H	0.4498	23.2207	29.3833	0.4499	23.2207	29.3833
140	C	-0.0387	19.4766	34.1848	-0.0387	19.4766	34.1848
141	H	0.5811	18.7652	34.45	0.5811	18.7651	34.4499
142	H	-0.4673	19.8512	34.9815	-0.4673	19.8512	34.9815
143	H	0.4564	20.1825	33.7174	0.4564	20.1825	33.7174
144	C	4.0408	19.2186	34.4418	4.0408	19.2186	34.4418
145	H	3.9934	19.9403	33.7821	3.9934	19.9403	33.7821
146	H	4.1231	19.6027	35.341	4.1231	19.6027	35.341
147	H	3.2262	18.6759	34.3936	3.2262	18.6759	34.3936
148	C	-1.8074	17.8185	33.9837	-1.8074	17.8185	33.9837
149	H	-2.3475	17.2662	33.3811	-2.3475	17.2662	33.3811
150	H	-2.3865	18.2187	34.6665	-2.3865	18.2187	34.6665
151	H	-1.1302	17.2585	34.42	-1.1302	17.2585	34.42
152	C	7.356	23.0873	28.3549	7.356	23.0873	28.3549

153	H	7.3375	22.8624	27.4014	7.3375	22.8624	27.4014
154	H	8.0463	23.7625	28.5187	8.0463	23.7625	28.5188
155	H	6.4826	23.4391	28.6251	6.4826	23.4392	28.6251
156	C	9.2713	21.5484	29.047	9.2713	21.5484	29.047
157	H	9.467	20.7126	29.5216	9.467	20.7127	29.5216
158	H	9.7738	22.2821	29.4592	9.7738	22.2821	29.4592
159	H	9.531	21.4599	28.1079	9.531	21.4599	28.1079
160	C	5.2983	18.3088	34.1419	5.2983	18.3088	34.1419
161	C	2.952	24.097	28.3681	2.952	24.097	28.3681
162	H	3.0139	25.0694	28.2608	3.0139	25.0694	28.2608
163	H	3.7139	23.6707	27.922	3.7138	23.6707	27.922
164	H	2.9668	23.8731	29.3227	2.9668	23.8731	29.3227
165	C	1.4131	24.2497	26.4106	1.4131	24.2497	26.4106
166	H	0.4645	24.165	26.1822	0.4645	24.165	26.1822
167	H	1.9552	23.8026	25.7269	1.9552	23.8026	25.7269
168	H	1.6572	25.1969	26.4536	1.6572	25.1969	26.4536
169	C	7.3298	22.0928	30.5893	7.3298	22.0928	30.5893
170	H	7.3637	21.2396	31.0711	7.3637	21.2396	31.0711
171	H	6.4304	22.4741	30.6637	6.4304	22.4741	30.6637
172	H	7.9804	22.7136	30.9815	7.9804	22.7136	30.9815
173	C	5.7136	17.1028	35.1602	5.7136	17.1028	35.1602
174	H	5.1457	16.3234	34.9931	5.1457	16.3234	34.9931
175	H	5.5933	17.4026	36.0867	5.5933	17.4026	36.0867
176	H	6.6526	16.8618	35.016	6.6526	16.8618	35.016
177	C	-2.0793	19.9698	32.8698	-2.0793	19.9698	32.8698
178	H	-1.6356	20.5985	32.2646	-1.6356	20.5985	32.2646
179	H	-2.353	20.4377	33.6861	-2.353	20.4377	33.6861
180	H	-2.8698	19.593	32.4289	-2.8697	19.593	32.4289
181	C	-0.1072	9.0453	20.3138	-0.1072	9.0452	20.3138
182	C	1.666	23.6031	27.7551	1.666	23.6031	27.7551
183	C	0.1859	6.5643	20.5939	0.1859	6.5643	20.5939
184	H	0.4982	6.3599	19.6894	0.4983	6.3599	19.6894
185	H	-0.7792	6.4061	20.6521	-0.7792	6.4062	20.6521
186	H	0.6504	5.9901	21.2389	0.6503	5.9901	21.2389

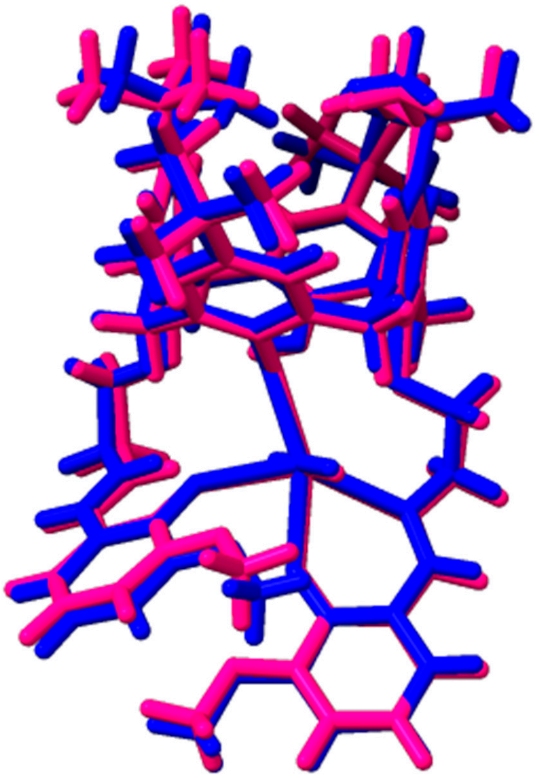

Figure S76: Overlayed calculated (pink) and experimentally determined (blue) molecular structure of complex **6**.

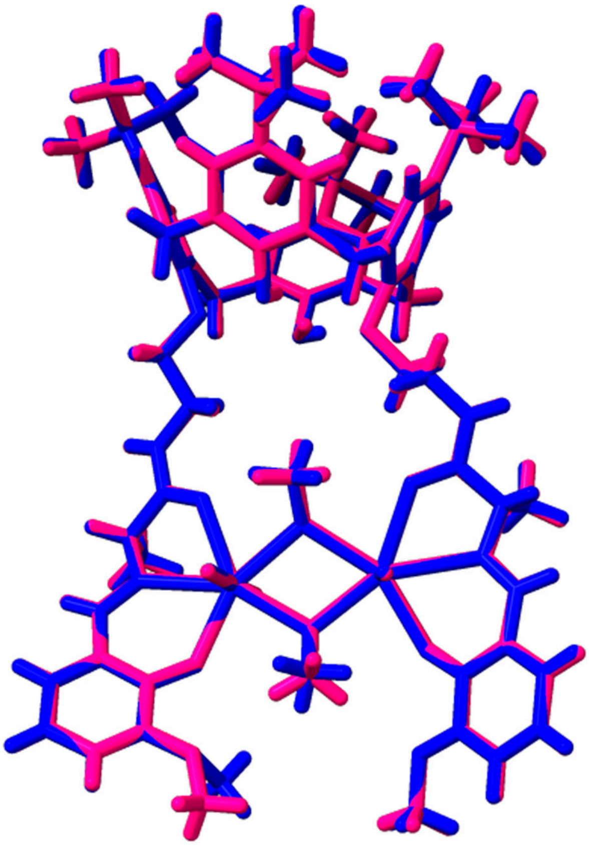

Figure S77: Overlayed calculated (pink) and experimentally determined (blue) molecular structure of complex **8**.

References

1 M. Green, J. Berman, *Preparation of pentafluorophenyl esters of FMOC protected amino acids with pentafluorophenyl trifluoroacetate, Tetrahedron Lett.,* **1990**, *31*, 5851-5852.

2 F. Michel, F. Thomas, S. Hamman, E. Saint-Aman, C. Bucher, J.-L. Pierre, *Galactose oxidase models: solution chemistry, and phenoxyl radical generation mediated by the copper status*, *Chem. - Eur. J.,* **2004**, *10*, 4115–4125.
